# Prognostic RNA-splicing archetypes in breast cancer identified by extended pre-training of histopathology foundation models

**DOI:** 10.1038/s41467-026-75217-z

**Published:** 2026-07-07

**Authors:** Lisa Fournier, Garance Haefliger, Albin Vernhes, Vincent Jung, Lena Loye, Valentine Du Bois, Intidhar Labidi-Galy, Pascal Frossard, Igor Letovanec, Cédric Vincent-Cuaz, Raphaëlle Luisier

**Affiliations:** 1 Idiap Research Institute, Martigny, Switzerland; 2 Center of Translational Research in Onco-Hematology, Faculty of Medicine, University of Geneva, Geneva, Switzerland; 3 Swiss Institute of Bioinformatics, Lausanne, Switzerland; 4 Signal Processing Laboratory (LTS4), School of Engineering, Ecole Polytechnique Fédérale de Lausanne (EPFL), Lausanne, Switzerland; 5 Department for BioMedical Research, University of Bern, Bern, Switzerland; 6 Department of Digital Medicine, University of Bern, Bern, Switzerland; 7 Department of Oncology, Hôpitaux Universitaires de Genève, Geneva, Switzerland; 8 Faculty of Medicine, Université de Genève, Geneva, Switzerland; 9 Department of Histopathology, Central Institute, Valais Hospital, Sion, Switzerland; 10 Institute of Pathology, Centre Hospitalier Universitaire Vaudois CHUV, Lausanne, Switzerland

**Keywords:** Tumour heterogeneity, Image processing

## Abstract

Recently, histopathology foundation models (hFM) have rapidly advanced in size and complexity, achieving excellent performance in cancer diagnosis and biomarker discovery. Here, we specialise pre-trained hFMs to invasive tumour tissue and present three key contributions. First, we systematically evaluate the biological concepts encoded in hFM representations across multiple biological scales. Second, we demonstrate that informed extended pre-training transforms generalist models into tumour-specialised ones encoding richer semantic information, enabling discovery of recurrent tumour archetypes with consistent morphological and molecular identities across patients. Third, we identify dominant tumour archetypes with aberrant gene-expression programs coexisting within tumours and recurring across heterogeneous epithelial cancers, including HER2-positive and triple-negative breast cancer. Crucially, these archetypes exhibit prognostic value, with RNA splicing-associated archetypes consistently predicting poorer outcomes. Our work shows that tumour-specialised hFMs unlock rich molecular and morphological information from routine H&E slides, providing computationally efficient and biologically informed solutions for biological discovery and patient stratification.

## Abstract (web summary)

Histopathology foundation models (hFMs) are able to extract molecular-level information directly from hematoxylin and eosin (H&E)-stained images. Here, the authors transform hFMs into tumor-specialized models via extended pre-training, enabling identification of prognostic RNA splicing-associated archetypes in breast cancer from routine H&E slides.

## Introduction

**Fig. 1:**
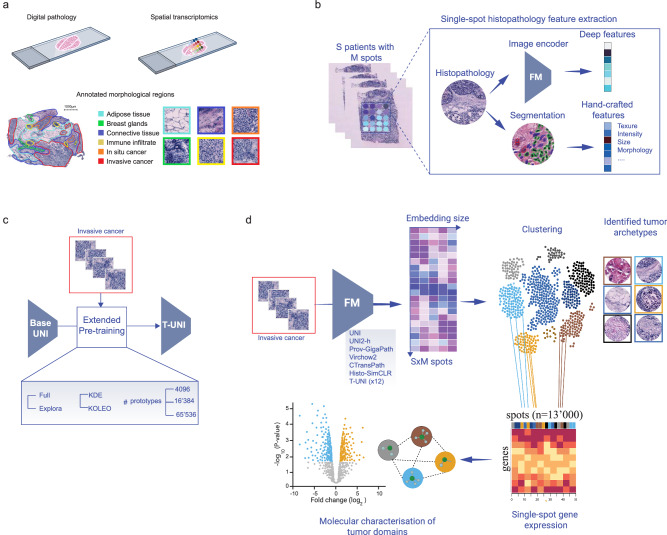
Overview of the strategy. **a** Schema of the publicly available datasets of breast tumors used in this study^36,37^, comprising H&E images, spatial transcriptomics data, and expert pathological annotations categorizing tissues into six distinct types: adipose tissue, breast glands, connective tissue, immune infiltrate, in situ cancer, and invasive cancer, from 7 HER2 patients and 92 TNBC patients. **b** From the H&E image patches, deep features are extracted using histopathology foundation models. In parallel, nuclei segmentation and classification are performed using CellViT^26^, and patch-level handcrafted features are computed to reflect nuclei composition, texture, colour and morphology as well as extracellular and whole patch colours and textures. **c** Extended pre-training of UNI was conducted using only invasive cancer patches, yielding tumour-expert T-UNI models. These models differ in their extended pre-training strategies, which include either ExPloRa or full retraining. Additionally, they vary in their regularisation losses, utilising either KDE or KoLeo, and in their number of learned prototypes set to 4,096, 16,384, or 65,536. **d** Embeddings of invasive cancer patches are extracted from the baseline hFMs and T-UNI models. Clustering using UMAP^38^-kmeans is performed to identify tumour archetypes in the image embedding spaces, which are then molecularly characterised by integrating ST data. Tumour archetypes are characterised by biological pathway enrichment analysis following marker gene extraction from differential gene expression analysis. Illustration partly created in BioRender. Fournier, L. (2026) https://BioRender.com/gpp3amj.

Intratumor heterogeneity (ITH) is a hallmark of cancer^1^ and a major driver of therapy resistance, particularly in heterogeneous malignancies such as breast cancer^2^. Accurately quantifying and characterising ITH is essential for improving patient stratification and predicting therapeutic outcomes. ITH has been extensively studied at the molecular level, particularly through single-cell RNA sequencing (scRNA-seq)^3,4^, revealing the coexistence of distinct tumour cell subpopulations within the same tumour, each defined by unique molecular identities linked to tumour invasion and therapy resistance^5–7^. However, how these distinct molecular programs spatially co-exist within tumours, forming regions defined by unique molecular profiles, tissue organisation, and composition, often referred to as “archetypes”, remains poorly understood. Recent advances in spatial transcriptomics (ST) and multi-omics technologies have begun to unravel the complexity of tumour ecosystems by enabling the spatial mapping of molecular programs within tumours^8^ (Fig. 1a). These studies have highlighted how tumour cell populations co-exist within distinct immune microenvironments, underscoring the critical role of spatial context in therapeutic outcomes^9,10^. However, most work to date has focused on tumour-immune interactions and does not address whether, and how, tumour cell subpopulations themselves organise into distinct tumour archetypes with potential clinical relevance. While molecular subtypes already guide preclinical models and treatment decisions in many cancers^10^, a scalable method to map the abundance and spatial organisation of tumour archetypes could be transformative for designing more rational combination therapies.

ITH extends beyond molecular variation to include diversity in cell shape, composition and organisation^11–15^. Importantly, many of these features are reflected in hematoxylin and eosin (H&E)-stained histopathology images, which remain a cornerstone of diagnostic pathology thanks to their rich morphological detail, affordability, and ease of use, making them a powerful and scalable modality compared to more complex techniques like spatial transcriptomics (ST). For example, breast cancer grading is based on multiple morphological criteria, including the degree of nuclear atypia, architectural patterns, and mitotic activity^16^. The full potential of histopathology to resolve *molecular* ITH, particularly in terms of underlying gene expression programs, remains however largely untapped, often due to the perception that it lacks the molecular resolution provided by techniques like ST^17^. This perception is reinforced by the fact that cancers with profoundly different molecular programs, and consequently distinct clinical behaviours and therapy responses, can appear morphologically indistinguishable to the human observer, not because the relevant visual information is absent, but because human perception is limited in its ability to process the full complexity encoded in histological images, typically focusing on diagnostically salient features^10^. However, recent breakthroughs in artificial intelligence (AI), particularly large-scale self-supervised models designed for histopathology, known as histopathology foundation models (hFMs), which learn the underlying structure of histopathology data directly from the data itself without requiring explicit labels (e.g., by predicting masked or missing regions from surrounding tissue), are now challenging this view. These models demonstrate the ability to extract molecular-level information (gene expression profiles and genetic mutations) directly from routine H&E images^18–23^. These results are consistent with growing evidence that morphological features, such as cell size, nuclear morphology, granularity, and spatial organisation, are closely linked to gene expression programs^24,25^. Collectively, these studies demonstrate that H&E images contain rich biological information, including molecular signals essential for resolving tumour heterogeneity, which can actually be retrieved using hFMs. While hFMs have demonstrated remarkable potential across tasks, from segmentation^26^ and survival prediction^27^ to tumour characterisation^28–32^, it remains unclear which biological concepts they truly capture and whether they can move beyond automating annotation-driven pathology to reveal structures beyond human visual perception. Fully harnessing their potential requires understanding how semantic information, defined here as biologically meaningful representations of tissue architecture, cellular composition, and molecular programs, is encoded within these models. Such insight is essential for guiding targeted modifications to model architectures and learning strategies, ensuring that learned representations are both biologically informative and aligned with discovery-driven objectives.

In this study, we specialise histopathology foundation models to uncover tumour archetypes directly from routine H&E slides, that map to spatially distinct molecular programs and provide a scalable framework for quantifying tumour heterogeneity with direct clinical relevance. Through a systematic comparison of six state-of-the-art pathology models, we provide a comprehensive evaluation of their representational capacity in capturing biologically relevant concepts (Fig. 1b). Next, we show that extended pre-training, which consists of continuing the pre-training phase with additional unlabeled invasive tumour tissue data, substantially enhances semantic representations, transforming generalist models into tumour-specialised ones capable of resolving subtle, recurrent archetypes shared across patients and validated by spatial transcriptomics (Fig. 1c, d). In contrast, conventional models predominantly capture patient-specific effects and fail to disentangle molecularly distinct tumour domains. Using our specialised models, we identify co-existing archetypes within the same tumour that vary in abundance and are specific to invasive carcinoma, revealing a previously unrecognised layer of tumour heterogeneity. Notably, we uncover archetypes associated with aberrant RNA metabolism, including alternative splicing, as a conserved and clinically adverse program in both HER2 -positive and triple-negative breast cancer. Together with immune-enriched archetypes, these features show strong prognostic value and appear generalizable to other heterogeneous epithelial cancers. Altogether, our study demonstrates that histopathology AI can unlock rich molecular and morphological information encoded in H&E images, offering new avenues for patient stratification and therapeutic targeting, all from widely accessible histopathology slides and low computational resources.

## Results

### Interpreting and quantifying biological representations learned by histopathology foundation models (hFM)

In recent years, histopathology foundation models (hFMs) have become powerful tools, enabling significant advances in automating tasks, including segmentation^26^, whole slide image (WSI) classification, and survival prediction^27^. By leveraging vast amounts of H&E data, these models learn to extract, without relying on predefined labels, rich vector representations in the form of numerical feature vectors, also referred to as *embeddings*, from histopathology images related to complex patterns. Benchmarking efforts have primarily focused on standard tasks such as segmentation or whole-slide image (WSI) classification^18,19,33–35^. However, these evaluations primarily emphasise maximising performance, with limited attention to the biological relevance and interpretability of the learned representations. In particular, hFMs produce high-dimensional representations, yet it remains unclear which semantic signals they encode or how choices in pretraining, regularisation, and model or data scale influence this information. To address this, we first conducted an analysis to characterize and quantify the semantic content encoded by six widely used hFMs (Histo-SimCLR^35^, CTransPath^34^, Virchow2^33^, Prov-GigaPath^19^, UNI^18^, and its updated version UNI2-h; Supplementary Data 1), using two publicly available breast cancer datasets corresponding to clinically important subtypes (HER2-positive and triple-negative breast cancer, TNBC^36,37^; Fig. 1a, Supplementary Fig. 1. These data-sets include H&E-stained image data, expert annotations of major tissue domains (breast glands, connective tissue, adipose tissue, immune infiltrate, invasive cancer, in situ cancer), and spatial transcriptomic data from 7 HER2-positive and 92 TNBC patients, respectively. In total, the cohort comprises 30 ST slides from HER2-positive tumours and 94 from TNBC tumours (Supplementary Fig. 1). After image pre-processing, we extracted patch-level vector representations from each model, as well as segmentation-based handcrafted features (Methods), to enable downstream interpretability analyses focused on understanding feature relevance across several biological scales (Fig. 1; Supplementary Data 1, 2).

**Fig. 2:**
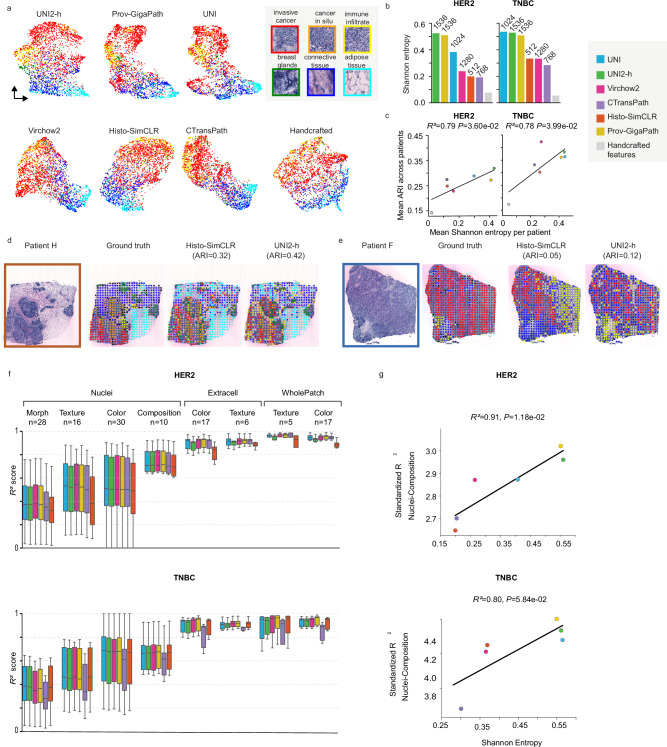
Assessing semantic richness in histopathology representations with entropy. **a** UMAP visualizations^38^ comparing patch embeddings of the HER2 dataset (${n}_{{patches}}=103023$) extracted from the 6 baseline hFMs and the patches’ handcrafted features, colored by tissue type as annotated by the pathologist. **b** Bar graphs of the Shannon Entropies (SE) associated with all patch representations from each hFM, as well as handcrafted features. The embedding size is indicated at the top for each model. Left: HER2 dataset; Right: TNBC dataset. **c** Scatter plot showing, for each model, the correlation between the average across patients of SE and ARI for tissue types. ${R}^{2}$ indicates the Pearson correlation coefficient and P the associated two-sided p-value for the null hypothesis of zero correlation. The line marks the linear regression fit. Left: on the HER2 dataset; Right: on the TNBC dataset. **d** Whole slide images (WSI) with ground truth spots annotations and spots clustering (UMAP k-means, k=6) based on SimCLR and UNI2 patches embeddings for patient H from the HER2 dataset. **e** Same as **d** for patient F from the HER2 dataset (UMAP k-means, k=3). **f** Boxplot showing the distribution of ${R}^{2}$ scores from linear regression models predicting handcrafted features based on patch embeddings from different hFMs. Results are grouped by feature category: nuclear morphology, nuclear texture, nuclear colour, nuclear composition, extracellular colour, extracellular texture, and colour and texture of the entire patch. The number of features per category ${n}$ is indicated under each plot. Boxplots display the median, the lower and upper quartiles, the minimum and maximum values. Upper: HER2 dataset; Lower: TNBC dataset. **g** Scatter plot of the correlation between the Nuclei-Composition category standardised R² and SE for the different hFMs on each dataset. ${R}^{2}$, P and the line marks are set similarly to those in **c**. Top: HER2 dataset; Bottom: TNBC dataset. Source data are provided as a Source Data file.

Visual inspection of the Uniform Manifold Approximation and Projection (UMAP) embeddings^38^ of patch-level representations revealed that major tissue components, such as adipose tissue, cancer regions, and immune cell infiltrates, naturally form clusters (Fig. 2a, Supplementary Fig. 2a). Accordingly, despite differences in scale and training, hFMs capture similar global patch properties, likely driven by low-level visual cues such as color and nuclear density. Notably, some models group tissue types more consistently, suggesting closer alignment with biologically meaningful distinctions. To quantify this, we evaluated each model’s performance to identify major tissue components using the Adjusted Rand Index (ARI) against ground-truth annotations and related these scores to the complexity of the representation space, measured by Shannon entropy (SE) (Methods). SE was computed from the variance ratios explained by the principal components of each hFM’s representations, modelled as a probability distribution (Methods). A high SE indicates that information is more evenly spread across the *N* dimensions of the representation space rather than being concentrated in a few. We hypothesised that models utilising the full capacity of the representation space would exhibit increased semantic richness. As shown in Fig. 2b, SE varies substantially across models and correlates to some extent with the representation dimensionality and model size, with UNI2-h (0.6 billion parameters) and Prov-GigaPath (1.1 billion parameters) exhibiting the highest SE on average across both datasets. Few exceptions are observable, such as UNI ranking first on TNBC and third on HER2, suggesting that smaller models can proportionally capture more concepts relevant to distinguishing cancerous breast tissues than certain bigger models (Supplementary Fig. 2b). Strikingly, model performance in tissue-type clustering is positively correlated with representation complexity, as measured by SE, across both linear and non-linear analyses (Fig. 2c, Supplementary Fig. 2c, d), supporting SE as an indicator of semantic richness. At the patient level, models achieved higher ARI scores on slides containing diverse tissue compositions (e.g. HER2 patient H), whereas slides dominated by a few tissue types (e.g. HER2 patient F) consistently posed greater challenges, highlighting limitations in low-variability samples (Fig. 2d–e, Supplementary Fig. 2e).

To understand the sources of variations in tissue-type clustering performance across models, we next evaluated which low-level histomorphological features are best represented in their embeddings. We thus tested how accurately their representations linearly predict 131 handcrafted descriptors of colour, texture, shape, and cell composition with a focus on nuclear and extracellular matrix characteristics (see Supplementary Data 2 for the full feature list. Biological relevance of the handcrafted features was confirmed by their ability to discriminate tissue types (Supplementary Fig. 2f). As shown in Fig. 2f, most models performed comparably in predicting handcrafted features, except Histo-SimCLR on the HER2 dataset and CTransPath on the TNBC dataset. Overall, models more strongly encoded texture- and colour-related features than morphological ones, and showed greater representation of extracellular and whole-patch descriptors, indicating a bias toward broader tissue context rather than individual cellular structures. Crucially, higher representation complexity (SE) was specifically associated with improved encoding of nuclear composition features, including nuclei counts and the density of inflammatory and tumour cells (Fig. 2g, Supplementary Fig. 2g). This suggests that the superior tissue discrimination achieved by high-SE models is driven by an enhanced capacity to capture semantically meaningful nuclear organisation. Consistent with observations in natural images^39,40^, these results indicate a progression from low-level visual cues toward higher-level semantic features, here reflecting nuclear composition and cellular density.

Together, these results indicate that SE is a reliable metric for assessing the biological relevance of learned representations, as demonstrated by its significant correlation with the model’s ability to accurately distinguish tissue types based on biologically meaningful differences. Furthermore, the increase in SE, and consequently, semantic richness, is specifically linked to nuclear density and composition.

### Extended pre-training enhances tumour-specific representations while reducing patient-specific bias

**Fig. 3:**
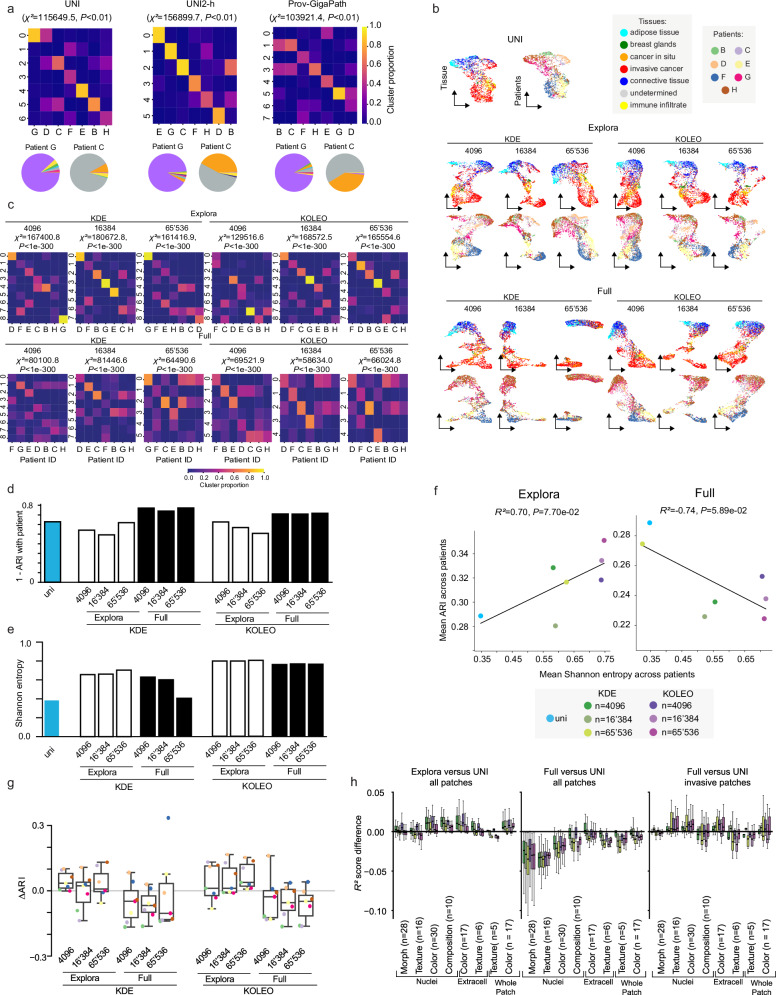
Extended pre-training exclusively with invasive cancer tissue enhances semantic representation on HER2 dataset. **a** Heatmap of patient spot distributions across predicted HER2 tumour archetypes, identified by clustering invasive cancer patches using embeddings from UNI, UNI2-h and Prov-GigaPath. Columns represent patients, and row colour intensity represents the proportion of spots assigned to each cluster. P-values from Pearson’s chi-square test of independence are reported. **b** UMAP visualizations^38^ comparing the HER2 patch embeddings from UNI and T-UNI models. Upper: coloured by tissue origin. Lower: coloured by patient origin. **c** Heatmap as in) using embeddings from T-UNI models. **d** Bar graphs of the batch effect mitigation score for UNI and T-UNI models on the HER2 dataset, defined as 1-”*ARI with patient”*, on invasive cancer patches. A high batch effect mitigation score indicates a low batch effect. **e** Bar graphs of the SE associated with the embeddings from UNI and T-UNI models on the HER2 dataset. **f** Scatter plot of the correlation between mean SE and mean tissue-type ARI across HER2 patients for UNI, and T-UNI models using either the ExPloRa (left) or the full (right) methods. ${R}^{2}$ indicates the Pearson correlation coefficient and P the associated two-sided p-value for the null hypothesis of zero correlation. **g** Boxplots of ARI score differences between T-UNI models and UNI, computed per HER2 patient. The 7 patients are represented by the data points. ${R}^{2}$ indicates the Pearson correlation coefficient and P the associated two-sided p-value for the null hypothesis of zero correlation. **h** Boxplots of R² score differences between T-UNI models and UNI, from linear regression predictions of handcrafted features, grouped by feature category. Linear regression using all patch embeddings for T-UNI models either trained with the ExPloRa (left) or the full (middle) methods, and using only invasive cancer patch embeddings for T-UNI models trained with full method (right). The number of features per category ${n}$ is indicated under each plot. Boxplots display the median, the lower and upper quartiles, the minimum and maximum values. Source data are provided as a Source Data file.

To assess whether richer biological representations translate into improved resolution of subtle intra-tissue heterogeneity within pathologist-annotated tumour regions, we next clustered invasive tumour patches into archetypes using each model’s embeddings. Surprisingly, archetypes identified by these models showed poor consistency across patients, with many individuals dominated by a single cluster (Fig. 3a, Supplementary Figs. 3a–d), indicating a strong patient-specific effect. Consistently, UMAP embeddings of UNI patch-level representations revealed clear separation by patient (Fig. 3b, top). These results indicate that resolving subtle morphological archetypes while remaining robust to patient-specific artefacts likely requires richer representations that are more specifically adapted to tumour tissue. To achieve higher semantic specificity, we hypothesise that extended pre-training (also referred to as self-supervised fine-tuning), in which the model continues training exclusively on relevant tissues^41,42^ (here, tumour tissue), enables the refinement of representations toward discriminative, tumour-adapted patterns that may not be fully captured during large-scale, generic pre-training. We therefore tested this method with UNI exclusively, as the training parameters for UNI2-h or Prov-GigaPath were not available at the time of the study. Additionally, owing to its smaller model size (Supplementary Data 1), UNI requires substantially fewer computational resources than the other models, making it a natural choice for this first study. Given the lack of established standards for extended pre-training, we systematically explored multiple strategies and hyperparameters (Methods, Supplementary Data 3). In brief, we compared “full training”, which updates all model weights, with ExPLoRA^41^, a partial weight update approach designed to better preserve prior knowledge. Additionally, we tested two regularisations (KDE^33^ and KoLeo^43,44^, see Methods) promoting better representation spread. Finally, models were trained with varying numbers of learned “prototypes”, representing feature centroids in the learned representation space and updated dynamically during training. Notably, we performed extended pre-training separately for the HER2 and TNBC datasets, as their associated tumour types exhibit distinct biological and morphological characteristics. Therefore, we experimentally found it most appropriate to specialise models separately for each subtype, to minimise technical confounding and ensure that learned features are truly cancer-specific. Consequently, all downstream analyses are performed independently on the two datasets, allowing us to assess the robustness and reproducibility of the findings across tumour types while avoiding batch-driven artefacts. This strategy yielded 12 tumour-specific models for the two breast cancer subtypes, hereafter referred to as T-UNI (Fig. 1c; Supplementary Data 3).

Repeating the clustering of invasive tumour patches using the new representations from all T-UNI models, we found that extended pre-training facilitates the identification of tumour archetypes that recur across patients (Fig. 1c). We next quantitatively assessed whether extended pre-training mitigates the *batch effects* observed for UNI, UNI2-h, and Prov-GigaPath, where clustering was dominated by patches originating from individual patients (Fig. 3a, b, Supplementary Fig. 3a–d). To this end, we defined a *batch effect mitigation score* as 1-ARI, computed using patient labels instead of tissue labels on patches from invasive cancer. As shown in Fig. 3d and Supplementary Fig. 3e, extended pre-training with the *full* strategy consistently results in lower alignment with patient identity across both datasets, suggesting improved robustness to batch effects and a shift toward patterns that may represent more universal tumour archetypes.

While reduced alignment with patient identity suggests improved robustness to batch effects, it is important to determine whether this reduction reflects suppression of technical and patient-specific artefacts rather than a loss of meaningful biological signal. In this regard, extended pre-training did not substantially alter the global biological organisation of the representations, which remained primarily structured by tissue type across training procedures (Fig. 3b). Notably, full training resulted in a clearer separation between invasive tumour tissue patches and other tissue types compared to the UNI baseline and models trained with ExPLoRa. The effect was particularly pronounced with KoLeo regularisation, which scatters representations more aggressively than KDE regularisation (Fig. 3b, lower). We further observed that ExPLoRA simultaneously improved SE and tissue type discrimination measured by ARI, demonstrating that extended pre-training with this method refines representations whilst keeping prior model knowledge of all tissue types (Fig. 3e–g; Supplementary Fig. 3f–h). In contrast, the full training setup decreases ARI scores while increasing SE, suggesting that it causes the model to lose previously encoded biological concepts essential for accurate tissue classification (Fig. 3e–g; Supplementary Fig. 3f–h). Full training with KoLeo was largely insensitive to the number of prototypes, whereas increasing prototype numbers in KDE progressively reduced SE and impaired the model’s ability to distinguish tissue types (Fig. 3e, Supplementary Fig. 3f). This suggests that excessive smoothing of representations across the model’s embedding space conflicts with the formation of clusters driven by prototypes and is detrimental to models’ ability to learn biologically meaningful distinctions.

We next asked whether the observed gains in semantic richness and reduced batch effects, particularly with the full strategy, correspond to improved encoding of specific, interpretable handcrafted features. ExPLoRa-based models showed enhanced encoding of features related to cell colour, composition, and texture, albeit at the expense of whole-patch texture descriptors (Fig. 3h, Supplementary Fig. 3i). In contrast, the full pre-training strategy resulted in a global reduction in handcrafted feature encoding across all patches, consistent with the observed decrease in tissue-type classification performance (Fig. 3f, g, Supplementary Fig. 3g, h). Notably, this trend reversed when restricting the analysis to invasive cancer patches, indicating that model specialisation enhances encoding of cancer-specific features while deprioritising features primarily relevant to other tissue types.

In summary, these analyses indicate that UNI, UNI2-h, and Prov-GigaPath models tend to prioritise global tissue characteristics rather than capturing finer distinctions between tumour regions. This is compounded by patient-specific effects that impair the detection of subtle archetypes within tumours. Crucially, we demonstrate that while full extended pre-training on invasive cancer patches reduces the model’s ability to distinguish general tissue types (e.g., breast glands, immune cells) and broad handcrafted features, it significantly enhances the model’s capacity to learn tumour-specific concepts and effectively mitigates patient-related batch effects. The consistently superior batch-effect mitigation score and Shannon entropy achieved by the KoLeo-regularised full pre-training provide strong support for prioritising this strategy over others for robust tumour archetype identification.

### Extended pretraining reveals recurrent molecular tumour archetypes imperceptible to routine histopathology

**Fig. 4:**
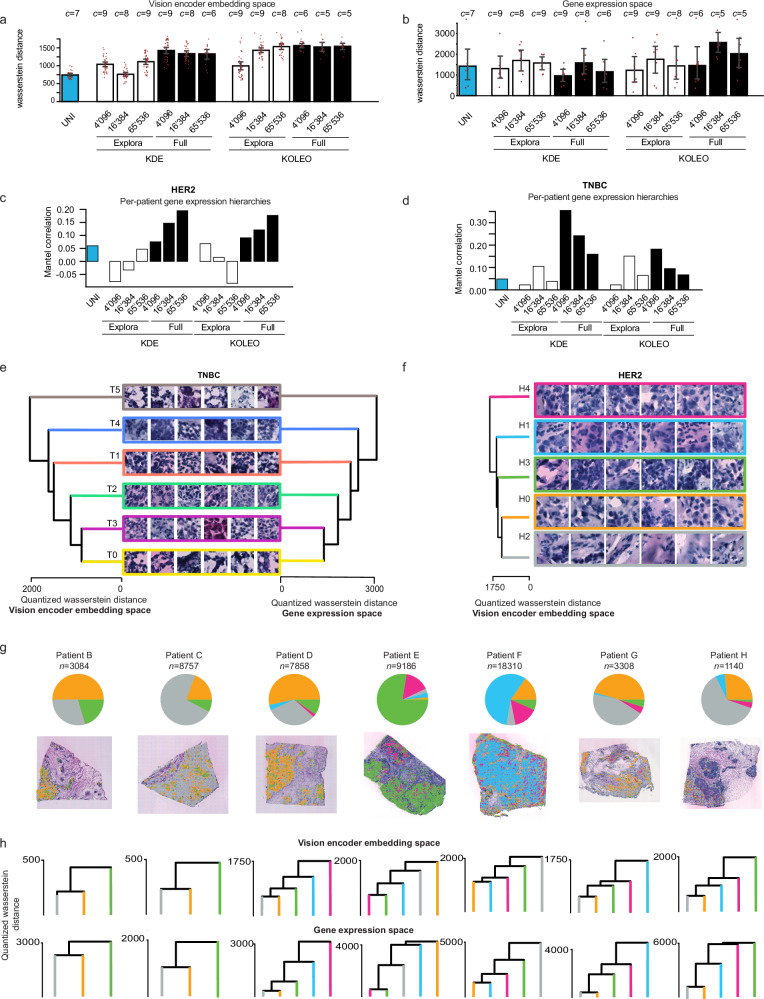
Extended pretraining enables the identification of molecularly defined tumour sub-archetypes. **a** Bar graphs of the average normalised quantised Wasserstein distances in the UNI and T-UNI embedding spaces between all HER2 tumour archetype pairs identified via unsupervised clustering of invasive cancer patches. The number of tumour archetypes c identified is indicated at the top for each model. It is selected to maximise the silhouette score while maintaining a high batch-effect mitigation score (Methods). Bar height represents the mean (±95% CI) of pairwise quantised Wasserstein distance between clusters. **b** Distributions of the median quantised Wasserstein distances between HER2 tumour archetype pairs in the gene embedding space, computed per patient. Archetypes are identified via unsupervised clustering of invasive cancer patches in the UNI and T-UNI embedding spaces. The number of tumour archetypes c identified is indicated at the top for each model. Bar height represents the mean (±95% CI) across patients of the per-patient median pairwise quantised Wasserstein distance between clusters. **c** Mean Mantel correlations of gene expression hierarchies across patient pairs for each T-UNI model in the HER2 dataset. **d** Same as **c** for the TNBC dataset. **e** Hierarchical clustering of TNBC tumour archetypes identified with the best TNBC T-UNI model (full retraining, KoLeo loss, 4 K prototypes). Clustering was performed using complete linkage on quantised Wasserstein distances between archetypes. Left: clustering in the T-UNI embedding space. Right: clustering in the gene expression space. **f** Same as (**e**) but for the HER2 tumour archetypes and using the best HER2 T-UNI model (full retraining, KoLeo loss, 16 K prototypes). Clustering is performed in the T-UNI embedding space. **g** Pie chart showing the relative abundance of the 5 HER2 tumour archetypes identified with the same T-UNI model. Upper: Relative abundance of morphological tumour archetypes per patient. Lower: Invasive cancer patches colored according to their tumour archetype in patients’ tumour sections. **h** Patient-wise hierarchical clustering of tumour archetypes identified for the HER2 dataset with the same T-UNI model. Clustering was performed using complete linkage on quantised Wasserstein distances between archetypes. Upper: clustering in the T-UNI embedding space. Lower: clustering in the gene expression space. Source data are provided as a Source Data file.

Having demonstrated that extended pre-training reduces patient-specific effects while enabling the identification of tumour archetypes that recur across patients, we next sought to determine which of the resulting models best captures biologically meaningful intra-tumoral tissue states. At this stage, the identified archetypes are defined purely by histopathological features, and their biological relevance remains uncertain. We therefore hypothesised that models capable of disentangling morphologically distinct tissues within the same tumour that also correspond to coherent and differentiable molecular profiles are more likely to reflect genuine biological heterogeneity rather than technical artefacts. To test this hypothesis, we leveraged spatial transcriptomics data matched to the corresponding H&E tumor patches, extracted gene expression profiles at the individual spot level, and grouped spots by archetype to obtain archetype-specific gene expression distributions (Fig. 1). We next quantified the separation between archetypes by computing pairwise distances between their distributions in two complementary spaces: (i) the vision embedding space learned by the deep models and (ii) the gene expression space derived from the spatial transcriptomic profiles of spots assigned to each archetype. In both cases, distances were computed using the Wasserstein distance (Methods), which captures differences between distributions while accounting for all spots rather than relying on summary statistics. Using this approach, we found that archetypes derived from T-UNI models were consistently more separated in the vision space (Fig. 4a and Supplementary Fig. 4a), whereas separation in the gene expression space was largely comparable to that observed for the UNI baseline (Fig. 4b and Supplementary Fig. 4b). However, in both HER2 and TNBC datasets, one or two T-UNI variants, in particular those trained in full settings, exhibited increased molecular distances between clusters. The models achieving the highest silhouette scores in the vision space while effectively mitigating batch effect (Supplementary Fig. 4c, d) are the full KoLeo models, using 16k prototypes for HER2 and 4k prototypes for TNBC. For these two models, the average molecular distance between clusters in the gene expression space exceeds the one obtained with UNI (Fig. 4b and Supplementary Fig. 4b).

Given the inherent variability and technical noise present in clinical data, we reasoned that biologically meaningful tumour archetypes should exhibit consistent molecular relationships across patients. Specifically, if hierarchical clustering of archetypes based on their gene expression profiles yields similar structures when computed independently for each patient, this would indicate that the model captures shared biological organisation rather than patient-specific artefacts or random variation. To assess this, we quantified the consistency across patients of the hierarchical clustering structures derived from archetype-level gene expression profiles. We found that models trained with the full T-UNI KoLeo strategy (16 K prototypes for HER2 and 4 K for TNBC) not only achieved the strongest batch-effect mitigation (Fig. 3c, d, Supplementary Fig. 3e), but also produced substantially more consistent molecular clustering structures across patients compared to UNI (Fig. 4c, d). Moreover, in TNBC, hierarchical clustering of the six archetypes in the gene expression space, computed across all 92 patients, perfectly recapitulated the structure observed in the image embedding space (Fig. 4e). Based on these results, T-UNI full KoLeo (16 K prototypes for HER2 and 4k for TNBC) were selected for further investigation of tumour heterogeneity in breast cancer.

As discussed above, reproducibility of tissue archetypes across patients is a key indicator of model robustness. In the HER2 cohort, tumour archetypes identified by T-UNI (full KoLeo, 16 K prototypes; Fig. 4f) were consistently represented across patients (Fig. 4g, *top*), in contrast to those derived from UNI, UNI2-h, and Prov-GigaPath (Supplementary Figs. 3a–c). Visual inspection of hierarchical clustering based on gene expression profiles further confirmed the previously observed consistency of archetype relationships across patients, as well as their correspondence with the organization in the image embedding space (Fig. 4h). Notably, two HER2 archetypes, H0 (orange) and H2 (gray), which exhibit the highest morphological and structural similarity in the image space (Fig. 4f), were consistently identified across all patients (Fig. 4g, top). Consistent with the image-based results, these two archetypes also displayed the closest molecular profiles in most patients (Fig. 4h, bottom). Despite their morphological similarity, H0 and H2 showed distinct spatial organisation within tumours: H0 was broadly distributed throughout the tumour mass, whereas H2 formed localised, high-density regions within invasive cancer areas (Fig. 4g, bottom).

Together, these results highlight the superior robustness of KoLeo-based full extended pre-training over baseline models in identifying biologically meaningful tumour archetypes, and demonstrate that the proposed approach resolves subtle intra-tumoral tissue states that recur across patients, exhibit distinct molecular identities, and occupy specific spatial niches within tumours.

### RNA-splicing tumour archetypes are recurrently detected in HER2 and TNBC invasive breast cancers

**Fig. 5:**
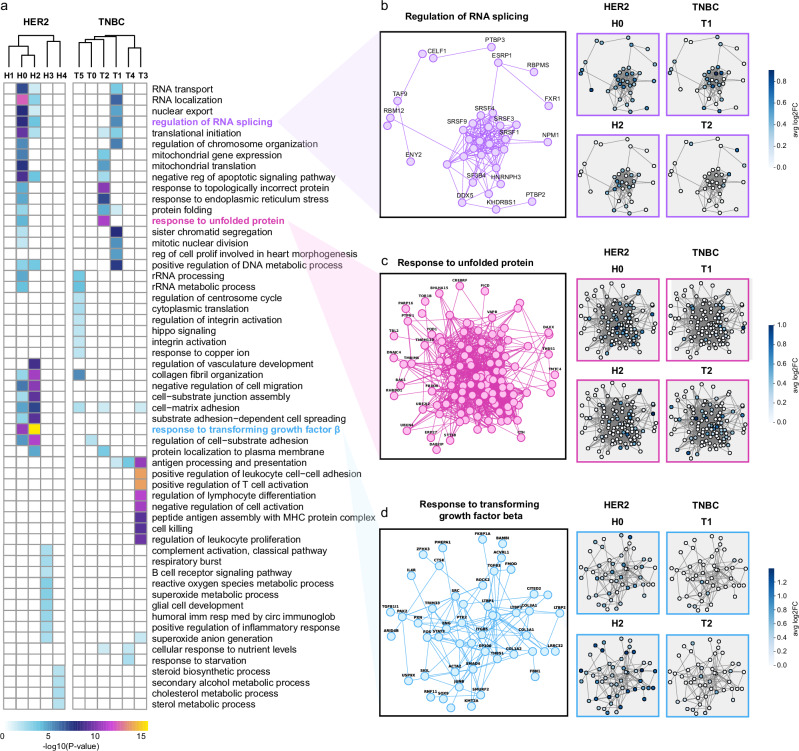
Histopathology-derived tumour archetypes reveal distinct molecular pathways in invasive breast cancer. **a** Heatmap of the biological pathway enrichment analysis of differentially expressed genes (using MAST^84^; log2FC > 0.25 and *p*-value < 0.01) between the tumour archetypes identified with the best T-UNI for each dataset (full training strategy, KoLeo loss, 16 K prototypes for HER2 and 4096 prototypes for TNBC). The -log10 p-value is reported for the top 10 significantly enriched pathways in each tumour archetype, provided that each pathway contains at least 5 of the identified marker genes. Redundant pathways have been curated manually. Hierarchical clustering is performed using 1-pearson correlation as the distance metric, and the complete method from *hclust* function in the *stats*^91^ R package. Pathway enrichment was assessed using g:Profiler (gprofiler2) with a one-sided hypergeometric test against the GO Biological Process database, with family-wise error rate controlled via a permutation-based threshold. **b** Protein–protein interaction (PPI) network of RNA splicing regulation. Left: reference PPI network for the RNA splicing pathway, shown without differential expression colouring. Right: the same network shown for four tumor archetypes, archetypes H0 and H2 from HER2 dataset and archetypes T1 and T2 from TNBC dataset, with node colours indicating the average log₂ fold change of each gene expression in the corresponding tumour archetype relative to all other archetypes from the same dataset. Nodes represent proteins, edges represent experimentally determined protein-protein interactions annotated in the STRING database^92^. **c** Same as **b** for the response to unfolded protein signalling pathway. **d** Same as **b** for the response to transforming growth factor beta signalling pathway. Source data are provided as a Source Data file.

Having identified robust tumour archetypes with distinct spatial and molecular profiles, we next examined their specific molecular identities using differential gene expression (DGE) analysis. As a baseline validation, pathway enrichment across pathologist-annotated tissue types recovered expected biology, for example, complement activation in immune tissue and vascular regulation in connective tissue (Supplementary Fig. 5a). Beyond these expected patterns, the analysis also revealed molecular programs distinguishing in situ from invasive cancer: DNA repair and chromosomal organization pathways were enriched in in situ lesions, whereas invasive tumours were marked by pronounced dysregulation of RNA splicing. TNBC invasive tumours additionally exhibited metabolic alterations, consistent with previous reports^45^. Notably, rather than representing attenuated or spatially diffuse versions of the global pathway signatures obtained when all invasive tumor regions are analysed as a single homogeneous group (Supplementary Fig. 5a), each archetype was associated with distinct and spatially localized molecular programs in both HER2 and TNBC (Fig. 5a). In contrast, clusters derived from UNI showed similar pathway enrichments across multiple archetypes, indicating limited ability to resolve subtle yet biologically meaningful variation within invasive tissue (Supplementary Fig. 5b). For example, TGF-β signaling, an established driver of proliferation, invasion, angiogenesis and metastasis^46,47^, was primarily enriched in cluster H2 of the HER2 subtype, a low-abundance archetype recurrent across all patients. Its spatial proximity to connective tissue (Supplementary Fig. 5a) and its enrichment for extracellular matrix-related pathways suggest that H2 may capture a compartment undergoing epithelial-to-mesenchymal transition (EMT). Likewise, metabolic activation observed in TNBC invasive cancer was similarly restricted to a specific archetype (T2). RNA-splicing dysregulation, a hallmark uniquely associated with invasive disease across both subtypes, showed a markedly stronger enrichment in a single archetype in each dataset (H0 in HER2 and T1 in TNBC). Protein-protein interaction analyses for marker genes associated with RNA splicing (clusters H0 in HER2 and T1 in TNBC), TGF-β response (cluster H2 in HER2), and immune antigen presentation (cluster T3 in TNBC) further confirmed the preferential activation of these pathways in each subtype, while highlighting RNA splicing as a shared aberrant program in advanced HER2 and TNBC (Fig. 5b–d). The spatial specificity of these molecular pathways within invasive tumours was further confirmed by visualising the expression of representative genes on HER2 slides, TGFB1I1 for TGF-β signalling, and ENY2 for RNA splicing, which displayed distinct spatial activation patterns, reinforcing that these pathways are linked to separate tumour regions (Supplementary Fig. 5c).

Together, these findings demonstrate that invasive breast cancers comprise multiple co-existing, molecularly distinct tissue compartments that are undetectable by routine histopathological assessment. Our extended pre-training strategy enables the identification of more subtle tumour archetypes than UNI and reveals specific biological pathways that localise to defined spatial domains within tumours. Notably, RNA splicing emerges as a previously unrecognised molecular signature common to both HER2 and TNBC invasive cancers, suggesting a convergent program associated with advanced disease.

### RNA splicing as a prognostic biomarker in breast cancer with potential to generalise across epithelial tumours

**Fig. 6:**
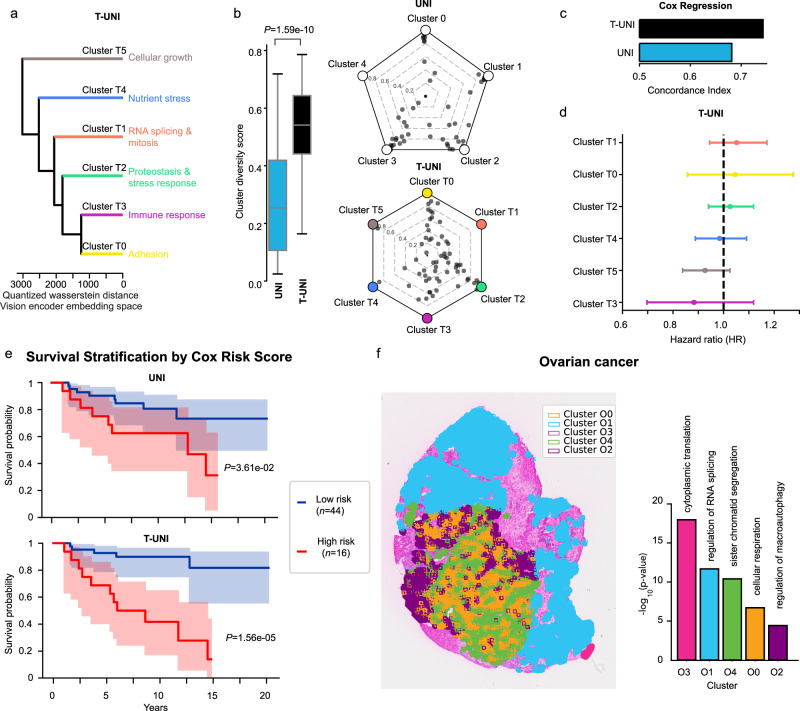
RNA splicing as a prognostic biomarker in breast cancer with potential to generalize across epithelial tumors. **a** Hierarchical clustering of TNBC tumour archetypes identified by the T-UNI model (full retraining, KoLeo loss, 4 K prototypes), using complete linkage and quantised Wasserstein distance computed in the T-UNI embedding space. Keywords summarise the biological pathways enriched in each archetype. **b** Left: Boxplots showing the distribution of archetype diversity scores across patients for UNI (5 archetypes) and T-UNI (6 archetypes; full retraining, KoLeo loss, 4 K prototypes). For each patient with more than 100 invasive cancer spots (UNI: *n* = 69, T-UNI: *n* = 62), cluster diversity was quantified using normalised Shannon entropy of the cluster-proportion distribution (range 0–1, with 1 indicating equal representation of all archetypes and 0 dominance by a single archetype). Boxplots show the median (centre line), interquartile range (IQR; box bounds, 25th–75th percentiles), 1.5×IQR (whiskers). Differences between UNI and T-UNI were assessed using a two-sided Mann-Whitney U test. Right**:** Simplex plots illustrating patient-level archetype compositions for UNI (upper) and T-UNI (lower). Each vertex represents an archetype and each point a patient (UNI: *n* = 69, T-UNI: *n* = 62); proximity to the centre indicates balanced representation; proximity to a vertex indicates single-archetype enrichment. **c** Bar plot of the concordance indexes of Cox proportional hazards models fitted using archetype proportions from UNI and T-UNI (full retraining, KoLeo loss, 4 K prototypes). **d** Forest plot showing hazard ratios and 95% confidence intervals per tumour archetype, derived from Cox model fitted using T-UNI cluster proportions. *n* = 60 patients, after exclusion of patients with fewer than 100 invasive cancer patches in either models (UNI and T-UNI). **e** Survival stratification using Cox-model risk scores. Kaplan–Meier curves (centre) show patient survival stratified into high- and low-risk groups based on risk scores derived from Cox models fitted with UNI or T-UNI cluster proportions; shaded bands represent 95% confidence intervals calculated using Greenwood’s formula. The high-risk group includes the 16 patients with the highest predicted risk scores, matching the number of observed deaths in the dataset. *P*-values were computed using a log-rank test. **f** Preliminary results on high-grade serous ovarian carcinoma. Left: tumour archetypes identified by UNI, right: top-enriched pathway per cluster. Source data are provided as a Source Data file.

TNBC is particularly challenging to treat because of its well-documented heterogeneity^48,49^. The tumor archetypes identified from H&E, that correspond to six clearly separated categories of biological pathways, offer a principled way to characterize this diversity with the potential to be widely applied given the standard use of such data in the clinic (Fig. 6a). To quantify patient-level tumor heterogeneity, we first computed the normalized Shannon entropy (SE) of each patient’s archetype-proportion distribution, with higher SE indicating that the tumor is composed of a broader mixture of archetypes (Fig. 6b, Supplementary Fig. 6a). T-UNI showed significantly greater heterogeneity across six archetypes (Fig. 6b, lower) than the five archetypes identified using UNI, which tends to assign each tumour almost exclusively to a single archetype (Fig. 6b, upper).

To assess the clinical relevance of the improved detection of tumour heterogeneity and the resulting archetypes, we next evaluated whether these features enable improved stratification of patients by survival risk. Cox models (Methods) fitted with archetype proportions derived from T-UNI achieved a higher concordance index (C-index) than those derived from UNI, indicating that the former provides more clinically meaningful information for predicting time to death (Fig. 6c, Supplementary Fig. 6b). Moreover, consistent with previous reports showing that immune-rich or TIL-high TNBC tumors exhibit improved survival and treatment response^50–52^, our immune-associated archetype (T3) was linked to better prognosis (Fig. 6d). In contrast, the archetype characterized by alternative-splicing activity (T1) contributed the second-largest effect in the model and was associated with poorer prognosis. Prior studies have demonstrated that alternative-splicing signatures can stratify breast cancer patients into distinct risk groups, with high-risk splicing programs predicting reduced survival^53,54^. Our findings extend this observation by showing that such splicing signatures can be inferred directly from digital histopathology and that their abundance is significantly associated with overall survival. Together with immune-related archetypes, these features emerge as robust prognostic factors. Kaplan-Meier analysis (Methods) further confirmed the clinical relevance and prognostic value of the H&E-derived archetypes: the six archetypes identified by T-UNI showed markedly stronger separation of survival curves (*P* < 0.001) than the five archetypes obtained with UNI (*P* < 0.05; Fig. 6e, Supplementary Fig. 6c).

Having shown that alternative-splicing signatures can be inferred directly from histopathology in two aggressive and heterogeneous subtypes of breast cancer, we next asked whether this capability generalises to other highly heterogeneous epithelial malignancies. We therefore examined high-grade serous ovarian carcinoma (HGSOC), a disease characterised by pronounced molecular and morphological diversity^55–57^, to determine whether AS-associated archetypes could similarly be recovered from H&E images. To this end, we generated spatial transcriptomics data from one HGSOC sample collected from a patient with disease resistant to therapy enrolled in the Nab-PIPAC trial (NCT04000906). Although the limited tissue area restricted our ability to perform extended pre-training on this sample, the UNI base model nevertheless recovered five preliminary tissue archetypes with distinct molecular profiles, predominantly in the largest tumor (Fig. 6f). Notably, one archetype was characterized by alternative-splicing activity and localized to spatially homogeneous regions of the tissue, whereas archetypes associated with DNA replication and cellular respiration were more interspersed and co-localized within a shared tumor domain.

Together, these results provide the evidence that aberrant RNA metabolism, including alternative splicing, is linked to spatially constrained, morphologically defined tumour domains that can be directly inferred from routine histopathology. These molecularly informed archetypes carry strong prognostic value, particularly when considered alongside immune-enriched archetypes, and the ability to recover such signatures appears generalizable to other heterogeneous epithelial cancers.

## Discussion

Tumour heterogeneity remains one of the central challenges in cancer biology and a major barrier to effective therapy. While histopathology slides contain rich morphological cues that reflect underlying cellular and molecular states, the extent to which this information can be decoded to reveal previously unrecognised biological structure remains largely unexplored. At the same time, histopathology foundation models (hFMs) have expanded rapidly in scale and performance, achieving state-of-the-art results on annotation-driven tasks such as segmentation, diagnosis, biomarker prediction, treatment response estimation, and outcome prognosis^18,23,58–60^. Yet these benchmarks largely evaluate capabilities that pathologists or standard genomic assays can already perform, raising the question of whether such models can move beyond automated workflow replication to uncover new biological insights, including the latent organisation of tumour ecosystems.

Our work addresses this gap through an in-depth investigation of heterogeneous epithelial cancers, focusing on two invasive breast cancer subtypes (HER2+ and TNBC) and complemented by a preliminary analysis of ovarian tumours. We first benchmarked state-of-the-art hFMs across biological scales, revealing that their representations are dominated by nuclei composition and global texture and colour properties. The strong sensitivity to staining variability likely contributes to patient-specific effects we observed, underscoring the critical need to explicitly address and control colour heterogeneity in future model development. We also introduce a semanticity measure based on Shannon entropy, which enables unsupervised model selection and correlates strongly with models’ ability to discriminate subtle tissue features. Nevertheless, we found that baseline hFMs tend to encode global tissue context at the expense of resolving spatially distinct intratumoral regions with unique molecular profiles, an essential capability for quantifying ITH and improving patient stratification.

To address this limitation, we investigated extended pre-training strategies that refine model specialisation on tumour tissue. A global update of model weights with KoLeo regularisation provided the best balance between tissue clustering, batch-effect robustness, and biological feature encoding. These refined representations enabled the discovery of tumour archetypes corresponding to spatially discrete molecular programs, consistent with the notion that underlying molecular alterations manifest as reproducible morphological and cellular patterns^28–32^. Notably, molecular pathways previously implicated in aggressive breast cancer^61–64^, including alternative splicing^53,62^, TGF-β signaling^65,66^, and ER stress^67,68^, occupied distinct spatial domains within the same tumour and were most accurately resolved by our specialised hFMs. These archetypes therefore offer a new framework for characterising tumour heterogeneity, revealing morphological manifestations of key oncogenic programs.

Importantly, these archetypes also exhibited strong clinical relevance. Alternative splicing-enriched archetypes were consistently associated with poorer survival, whereas immune-associated archetypes conferred favourable outcomes. Although validation in larger, independent cohorts will be required before clinical translation, the present results demonstrate that the identified archetypes achieve high-precision prognostic performance. Preliminary analyses in ovarian cancer, another highly heterogeneous epithelial malignancy, suggest that alternative-splicing archetypes can also be identified in these tumours, motivating future studies on their potential utility for patient stratification and therapeutic decision-making. Given the emergence of RNA-based therapeutics as promising strategies in complex diseases, including cancer^69–72^, the ability to detect spatially localised activation of alternative splicing from routine histopathology may help identify patients who could benefit from splicing-targeted or RNA-modulatory therapies.

Our methodological contributions span both computational biology and machine learning and open several avenues for future work. Extending these analyses to the single-cell level may reveal finer biological semantics encoded by token-level representations. Increasing cohort size and applying extended pre-training to multimodal pathology models, including immunohistochemistry or multiplexed imaging, may uncover richer archetypes. Furthermore, evaluating how computationally defined tumour archetypes relate to therapy response and microenvironmental interactions across larger cohorts and cancer types will be essential to fully assess their clinical potential. Additionally, our findings have important implications for spatial transcriptomics (ST). Existing ST analysis pipelines rarely integrate histopathology, and those that do typically rely on spatial adjacency for denoising or manual region annotation by pathologists. These approaches risk overlooking biologically meaningful but visually subtle tumour subtypes. Here, we introduce a histopathology-driven, unsupervised method that automatically identifies tumour archetypes directly from the data, without relying on predefined annotations.

**Fig. 7:**
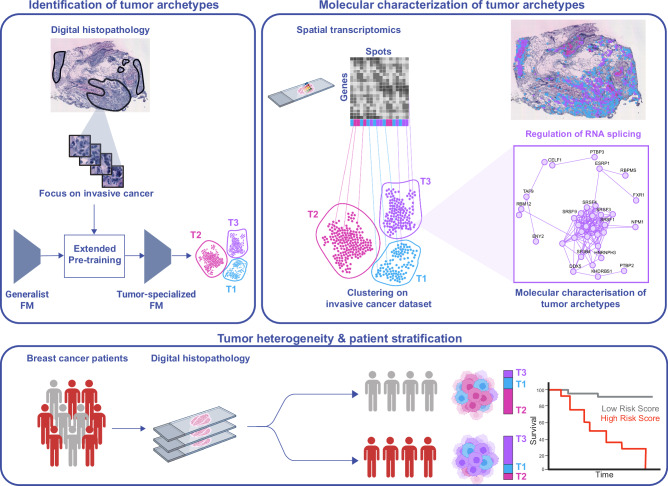
Graphical summary of the strategy and results. Schematic overview of the histopathology-driven, unsupervised framework for tumour archetype discovery, integrating extended pre-training of foundation models, spatial clustering of invasive cancer patches, molecular characterisation of image-derived archetypes, and downstream association with patient survival outcomes. Illustration partly created in BioRender. Fournier, L. (2026) v99ols8 https://BioRender.com/.

In conclusion, our results demonstrate that histopathology foundation models can be specialized in a computationally efficient manner, even with limited resources, to uncover clinically meaningful tumor heterogeneity from standard-of-care histopathology (Fig. 7). By revealing molecularly distinct tissue domains that are invisible to routine visual assessment, this approach accelerates large-scale cancer research and opens new avenues for precision diagnosis, risk stratification, and treatment selection using data already generated in routine clinical practice.

## Methods

### Ethics statement

The ovarian cancer sample was collected from a patient enrolled in the Nab-PIPAC trial (NCT04000906). The study was approved by local ethical committee (CCER 2024-01382) and was conducted in accordance with the Principles of Good Clinical Practice and the Declaration of Helsinki. The patient provided written informed consent.

### Datasets

#### Breast cancer datasets

We used two publicly available datasets. The HER2-positive breast cancer dataset^36^ originally comprised 8 patients with a total of 36 whole-slide images (WSIs) acquired at a resolution of 1 µm/pixel. One patient (patient A) was excluded from the analysis because the corresponding images showed suspected technical artefacts (Supplementary Fig. 7a). The final HER2 dataset therefore included 7 patients and 30 WSIs. The triple-negative breast cancer (TNBC) dataset^37^ originally includes 281 high-quality WSIs from 94 primary samples corresponding to 92 patients, acquired at 0.33–0.4 µm/pixel (Supplementary Fig. 1). For both datasets, one slide per patient was annotated by expert pathologists. In the TNBC dataset, only these annotated slides were used, yielding a total of 94 annotated WSIs.

#### High-grade serous ovarian carcinoma dataset

One high-grade serous ovarian carcinoma sample was profiled with the Visium HD 3’end spatial transcriptomics kit. The sample was collected from a patient enrolled in the Nab-PIPAC trial (NCT04000906). The sample was collected after the patient was enrolled in the trial, immediately prior to the start of the PIPAC procedure and therefore before the initiation of treatment. The patient was diagnosed with heavily pretreated platinum-resistant ovarian cancer prior to the enrolment in the trial. Fresh-frozen tissue blocks were sectioned at 10 µm on a cryostat, and sections were placed on Visium HD 3’end slides following the manufacturer’s instructions of the Visium HD 3’end Tissue Preparation Handbook (CG000804). The Visium HD assay is compatible with H&E staining and requires slide thawing, washing, and equilibration in the appropriate buffers before staining and imaging. After H&E staining and imaging, probe hybridisation, probe ligation, slide preparation, probe release, extension, library construction, and sequencing were performed according to the Visium HD 3’end Spatial Gene Expression Reagent Kits User Guide (CG000805). Sequencing was carried out on an Illumina NovaSeq 6000 using paired-end reads (43 cycles Read 1, 10 cycles i7, 10 cycles i5, 50 cycles Read 2). Raw sequencing data were processed with Space Ranger v3.0 to map FASTQ files to the human reference genome, detect the tissue section, align sequencing data to the microscope image, and generate gene–barcode matrices for downstream analysis. The resulting H&E WSI was acquired at a resolution of 0.25 µm/pixel.

### Full patches datasets

#### Breast cancer patches datasets

Given the theoretical spatial transcriptomics (ST) spot diameter of ~100 µm and the imaging resolution of each breast cancer dataset, the corresponding square spot patches measure 100 × 100 pixels for HER2 and 253 × 253 pixels for TNBC. We therefore extracted patches of these sizes to match ST spot dimensions. All possible patches were retained for the HER2 dataset, while for the TNBC dataset, we randomly sampled up to 900 patches per slide due to the larger dataset size. In total, the final HER2 dataset contains 103,023 patches and the TNBC dataset contains 186,554 patches. Among the 103,023 HER2 patches, 11,582 correspond to spots with matched RNA-sequencing measurements, and 3139 of these are labelled. In the TNBC dataset, 101,954 of the 186,554 patches correspond to spots with RNA-sequencing data, of which 44,485 are labelled.

#### HGSOC patches dataset

Spatial transcriptomics data were generated using Visium HD 3′ with 2 µm neighbouring bins. To match the 100 µm spot diameter used in the breast cancer datasets, 50 × 50 neighbouring bins were aggregated into a single spot. Given the image resolution of 0.25 µm/pixel, we extracted 400 × 400 pixel image patches corresponding to each aggregated spot. Due to the limited tumour size, overlapping patches were permitted. The final dataset comprises 66,280 image patches with matched spatial transcriptomics profiles.

#### Patches annotations

The HER2 WSIs were annotated by a pathologist into six distinct histological regions: cancer in situ, invasive cancer, adipose tissue, connective tissue, breast glands, and immune infiltrate. In contrast, the TNBC WSIs were annotated into 15 histomorphological categories, three of which were further refined at the single-cell level using a machine-learning–based approach. This resulted in near–pixel-level annotations, requiring aggregation to patch-level labels to enable comparison with the HER2 dataset. Annotations were obtained from pre-annotated RDS files deposited by the authors providing pixel-level tissue classifications. Spots containing ≥50% background signal, defined as pixels labelled “Nothing,” “Artefacts,” or “Hole (whitespace)”, were excluded. For all remaining spots, pixel counts across tissue categories were converted to proportional representations. To harmonize the two datasets, the fine-grained TNBC annotations were collapsed into six broader categories corresponding to the HER2 scheme: (1) invasive cancer (“Tumor”, “Tumor region”), (2) cancer in situ (“in situ”), (3) immune infiltrate (“High TIL stroma”, “Lymphocyte”, “Lymphoid nodule”), (4) connective tissue (“Nerve”, “Acellular stroma”, “Stroma cell”, “Low TIL stroma”, “Vessels”), (5) breast glands (“Lactiferous duct”), and (6) adipose tissue (“Fat tissue”). Necrosis and heterologous elements were reassigned to an “undetermined” category. A confidence-based labelling procedure was then applied: each spot received a definitive label only if its dominant tissue category accounted for more than 75% of its pixel composition; otherwise, it was labelled as “undetermined.” In total, out of the 101,954 spot patches, 44,485 are labelled with high confidence, and the other 57,469 were left undetermined. The WSI of HGSOC has been annotated using CellViT^26^. CellViT was first applied to perform cell segmentation and cell-type classification. For each image patch, the proportion of each annotated cell type was then calculated. To identify patches containing invasive carcinoma, we analysed the distribution of tumour-cell proportions across all patches. This distribution was modelled using a bimodal Gaussian mixture, representing background/non-tumour patches and tumour-rich patches. Patches associated with the tumour-rich (foreground) Gaussian component were selected. A cutoff corresponding to the 95th percentile of this Gaussian component was applied, and only patches exceeding this threshold were retained as invasive cancer patches.

#### Patches preprocessing

During preprocessing, raw image patches extracted at a native resolution of 100 × 100 pixels for HER2, 253 × 253 pixels for TNBC and 400 × 400 pixels for the HGSOC were up- or down-sampled to 224 × 224 pixels via bilinear interpolation to conform with the nominal input dimensionality expected by standard pretrained hierarchical feature models (hFMs). Prior to model inference, pixel intensities were normalised using the ImageNet normaliser as requested by the hFM models. We also evaluated the impact of applying stain normalisation prior to feature extraction, using the Macenko^73^ method. While this procedure modestly reduced batch effects, it also led to a loss of biological signal, reflected by decreases in both the Shannon entropy of explained variance and the ARI scores. Consequently, stain normalisation was omitted from the final analysis.

### Invasive cancer patches datasets

To further investigate intratumor heterogeneity, we first extracted invasive cancer patches from the full datasets by selecting all patches falling within the corresponding annotated invasive cancer regions. Because only a small number of slides were annotated in the HER2 dataset, and many patches remained unlabeled in the TNBC dataset, we expanded the subset of invasive cancer patches using a k-nearest neighbours (k-NN) classifier trained on the labelled spot patches. We applied k-NN to all unlabeled patches in the full dataset and retained those classified as invasive cancer for downstream analysis. The optimal value of k was selected as the one maximising the median F1 score across patients, based on a grid search with k ranging from 3 to 21 in increments of 2. Although median F1 scores were comparable across hFMs (Supplementary Fig. 7b), we used predictions derived from Histo-SimCLR representations for the HER2 dataset (median F1 score = 0.82; median accuracy = 0.84; k = 7). The final HER2 invasive cancer dataset comprised 54,311 patches, including 5,683 spatial transcriptomics spots. For the TNBC dataset, we used predictions derived from UNI representations (median F1 score = 0.98; median accuracy = 0.98; k = 3), resulting in a final TNBC invasive cancer dataset of 52,320 patches, of which 28,521 corresponded to spots. These invasive cancer patch subsets were subsequently used to retrain the T-UNI models.

### Shannon entropy of explained variance

To evaluate the distribution of variance among the SVD singular vectors of the precomputed image representations, the normalised Shannon entropy ${SE}$ of the explained variance vector is computed as follows:

$${SE}\,=-\frac{{\sum }_{i=1}^{n}{r}_{i}\,\log ({r}_{i})}{log (n)}$$

where ${r}_{i}$ represents the proportion of the total variance explained by the i-th singular vector, and n is the total number of singular vectors. A low SE indicates that a few components account for most of the variance in the data. In contrast, a high SE suggests that the variance is spread across multiple dimensions.

### Handcrafted feature extraction

Nuclei segmentation was performed using CellViT^26^. Features related to the morphology and texture of the nuclei have been extracted using scMTOP^74^ and correspond to the “Nuclei-Morph” and “Nuclei-Texture” features, respectively. We have adapted scMTOP to add colour descriptors of the nuclei, referred to as “Nuclei-Color” features, namely mean, skewness, kurtosis and entropy of each RGB colour channel, as well as intensity. Finally, statistics on cell-type composition have been computed from cell labels produced by CellViT and are referred to as “Nuclei-Composition”. Features related to the extracellular matrix colour (“ExtraCell-Color” features) and texture (“ExtraCell-Texture”) have been computed using scikit-image^75^. Similarly, texture and colour features at the entire patch level have been extracted and are referred to as “WholePatch-Texture” and “WholePatch-Color”. The list of all handcrafted features and their descriptions is available in Supplementary Data 2.

### Regression for handcrafted features prediction

To elucidate a part of the semantic information encoded by hFMs, we predicted each extracted handcrafted feature individually from hFM representations using linear regressions. We employed a 5-fold cross-validation approach and reported the mean R² across the five folds for each handcrafted feature. In order to obtain a single metric representing a category of handcrafted features, we computed the standardised ${R}^{2}$ is as ${R}_{{standardized},{Category}}^{2}= {R}_{{mean}}^{2}\sqrt{n-1/}\sqrt{{\sum }_{i=1}^{n}{\left({R}_{i}^{2}-{R}_{{mean}}^{2}\right)}^{2}}$ with ${R}_{{mean}}^{2}=\,\frac{1}{n}{\sum }_{i=1}^{n}{R}_{i}^{2}$, where n is the number of features in the category and ${R}_{i}^{2}$ is the ${R}^{2}$ of the *i-th* feature in the category.

### Clustering of different tissues

Clustering was performed on image representations obtained from various hFMs or from our selected handcrafted features taken as a whole. Performance was measured using Adjusted Rand Index (ARI) scores computed using the original tissue types as true labels (“ARI with tissue type”). Before applying k-means clustering (with *k* fixed to the known number of classes), we computed two-dimensional UMAP embeddings to capture non-linear relationships in the image representations. Following Van Assel et al^76^., we evaluated a range of UMAP hyperparameters, testing *n_neighbors* values of {10, 30, 50, 100, 150, 200, 250, 300, 350, 400} and *min_dist* values of {0.001, 0.1}. For each combination of hyperparameters, we selected the configuration that maximised the Adjusted Rand Index (ARI) with respect to tissue type. Clustering was performed either on the full dataset or on each patient individually, with the UMAP parameter optimisation repeated for each analysis (i.e., for each patient and each model). The UMAP-k-means approach was compared to raw-k-means where k-means is directly computed on the raw image representations, and to SVD5-kmeans, which factors image representations using the first 5 principal components of SVD before performing k-means. UMAP-kmeans was chosen for its superior performance (Supplementary Fig. 2d, Supplementary Data 4).

### Extended pre-training of the UNI foundation model

Extended pre-training of UNI has been performed only on our selected invasive cancer patches to refine their learned representations. Two different methods have been employed and compared: 1) ExPLoRA^41^, a state-of-the-art technique based on LoRa^77^, which performs extended pre-training by allowing updates of a small set of additional parameters added to the first L−1 transformer layers and a full update of the L-th layer; 2) the standard full extended pre-training of the model, where all weights are updated. For each strategy, we compared two regularisation losses (KoLeo and KDE) and the number of prototypes these models depend on. The list of all models is provided in Supplementary Data 3.

#### Regularization losses

DINOv2^78^, the self-supervised learning approach utilised by UNI^79^, employs the Kozachenko–Leonenko (KoLeo)^43,44^ regularisation loss, which aims at maximising differential entropy via a convex regularizer applied independently over each batch sampled during training. It is defined as follows:

1 $${L}_{{keleo}}=-\frac{1}{n}{\sum }_{i=1}^{n}log \left({\rho }_{n,i}\right),\,{{{\rm{with}}}} \, {\rho }_{n,i}={mi}{n}_{j\ne i}d(f({x}_{i})-f({x}_{j}))$$

with n the number of samples, $f({x}_{i})$ the hFM representation of the i-th data point and d is the cosine distance.

By maximising the distances between neighbouring vectors, the KoLeo loss ensures that the learned representations are uniformly distributed within their space. However, Zimmermann et al^33^. argue that while KoLeo has been demonstrated to be effective in natural image pretraining with high sample diversity, when samples get very similar, such as in digital histopathology, the loss can approach infinity. Therefore, they suggest using the kernel density estimator (KDE) with the unnormalized von Mises-Fisher (vMF) kernel, which allows similar images to cluster more smoothly in the embedding space depending on the choice of inner kernel. The KDE loss is defined as follows:

2 $${L}_{{KDE}}=-\frac{1}{n}{\sum }_{i=1}^{n} log {\sum }_{j=1}^{n} exp \left({Kf}{\left({x}_{i}\right)}^{T}f\left({x}_{j}\right)\right)$$

with к a scaling constant and other terms are defined above. Both the KoLeo and KDE losses are tested in our analysis.

#### Prototypes

DINOv2 depends on the online clustering scheme SwAV^80^, which relies on learned prototypes, estimating centroids on the fly within the representation space learned by the model. These prototypes promote the formation of clearly distinct regions, each encoding distinct high-level concepts or patterns identified by the model. Therefore, the number of prototypes influences the level of fine-grained-ness of the clusters. In theory, a greater diversity of training images necessitates a larger number of prototypes to accurately cluster these images. However, evidence suggests that an excessive number of prototypes may lead to significant redundancies among them, a phenomenon termed as *partial prototype collapse*^81^. Additionally, increasing the number of prototypes incurs significantly higher computational costs. While UNI has been trained using 65536 prototypes, we decided to perform the extended pre-training using various prototype numbers: 4096, 16384 and 65536.

#### Hyperparameters selection

Apart from the different losses tested and the various prototype numbers, the same parameters as those chosen in UNI^18^ (available on their github repository), which itself follows previous work in computational pathology, were initially adopted. A small number of targeted modifications were then implemented: first, the warmup phase, typically used to gradually increase the learning rate at the beginning of training, was omitted because UNI had already undergone pretraining. The cosine decay schedule was started at a learning rate of $1\times 1{0}^{-4}$. Secondly, the final layer of the prototype was frozen after a significant increase in training loss was observed during its training (Supplementary Fig. 7c). Finally, the number of epochs was restricted to one, as no further improvement in the total training loss was detected beyond this point and the batch size was set to the largest value allowed by memory constraints (ranging from 16 to 40 for the HER2 dataset and fixed to 25 for the TNBC dataset). For all models, the extended pre-training lasted less than one hour on our commercial RTX 3090 GPU. Hyperparameters related to the regularisation losses were set according to default values in the literature, namely the Koleo regularisation strength is set to 0.1^18,78^, the KDE regularisation strength and its scaling constant are set to 0.05 and 5, respectively^33^. As per the extended pre-training hyperparameters, the default value of 64 is used for the LoRa rank, as described in ExPLoRA^41^.

### Identification of invasive cancer archetypes

Clustering was performed with the UMAP-k-means strategy on a newly defined subset of invasive cancer patches to identify tumour archetypes. In particular, we observed that models resulting from extended pre-training (especially full training) on the first invasive cancer subset become significantly better at distinguishing them from the other tissue types. Hence, the deep features from these refined models are used to get a better curated subset of invasive cancer patches following the same k-NN strategy as above, selecting the optimal value of *k* by maximising the median F1 score across patients, based on a grid search with *k* ranging from 3 to 21 (Supplementary Data 6). Then, hFM representations were embedded using UMAP, evaluating the same range of hyperparameters as in previous experiments (*n_neighbors* ∈ {10, 30, 50, 100, 150, 200, 250, 300, 350, 400} and *min_dist* ∈ {0.001, 0.1}). Because ground-truth labels were not available for this task, hyperparameter selection relied on unsupervised criteria. For each combination of UMAP parameters, we performed UMAP followed by k-means clustering with *k* ranging from 4 to 9. For each value of *k*, the UMAP parameters yielding the highest silhouette score were retained. The optimal number of clusters *k* was then chosen by jointly maximising (1) the silhouette score and (2) a “batch effect mitigation” metric defined as 1 − ARI(patient), where ARI(patient) is the ARI computed using patient identifiers instead of tissue labels. Higher values of this metric indicate reduced batch effects. To balance both objectives simultaneously, we computed the Euclidean distance of each (silhouette, batch-effect-mitigation) pair to the ideal point (1, 1), corresponding to perfectly separated clusters with no patient-specific clustering bias (Supplementary Fig.4a, Supplementary Data 6).

### Molecular characterisation of the image tumour archetypes

#### Filtering

The processed gene count matrices deposited on Zenodo were used^36,37^. Spots with no reads were excluded. For each patient, a pseudo-bulk expression vector was created by aggregating the gene expression vectors from all spots. These vectors were then log2-transformed. The resulting gene read density showed a bimodal distribution: a low-density peak for low-abundance genes or those with spurious mappings, and a high-density peak for reliably expressed genes. To identify these reliably expressed genes in each patient, a two-component Gaussian mixture model was applied to the log2-transformed pseudo-bulk expression vector using the R package mclust^82^ (Supplementary Fig. 7d). For the HER2 dataset, a gene was retained if, in all 7 patients, it had less than a 1% probability of belonging to the low-density component, resulting in a final set of 11,591 genes and 11,554 spots. For the TNBC dataset, a gene was retained if, in at least 10% of patients (i.e., 10 out of 92), it had less than a 1% probability of belonging to the low-density component, yielding a final set of 14,644 genes and 98,833 spots. The spatial transcriptomics (ST) data were then treated as single-cell data and the *NormalizeData* function from Seurat^83^ was used to normalise the data: gene counts for each spot were divided by the total counts of that spot and multiplied by 10,000, followed by log transformation. The resulting filtered expression matrices are referred to as the “gene expression space”.

#### Uncovering tumour archetype-specific biological pathways

To characterise molecularly the tumour archetypes identified by the vision encoders, we extracted the tumour spots and performed a differential gene expression (DGE) analysis between tumour archetypes using the *FindAllMarkers* function from Seurat^83^, with patients specified as latent variables, to identify differentially expressed genes while mitigating the patient batch effect present in the filtered gene expression space (Fig S4c, upper). The MAST^84^ method was used for differential gene expression (DGE) analysis. Up-regulated marker genes for each tumour archetype were identified by selecting genes expressed in at least 25% of the spots for the tested archetypes, with a minimum log2 fold change of 0.25 and a *p*-value of 0.01. Finally, using g:Profiler^85^, we extracted for each tumour archetype the top 10 significantly enriched GO biological pathways^86^ containing at least 5 of the identified marker genes. The exact same analysis has been performed to characterise the annotated tissues. Redundant pathways were manually curated for visualisations (Fig. 5a, Supplementary Fig. 5a, b), while the full uncurated lists are provided in Supplementary Data 7 and 8.

### Quantised Wasserstein distance

We use the Wasserstein distance, also known as the Earth Mover’s Distance, to assess the dissimilarity between tumour archetypes, i.e the singular tumour clusters identified by the previously defined strategies, in both image and molecular representation spaces. This distance, based on Optimal Transport, measures the minimal total distances to be covered to transport each point of a distribution to those of another^87^. Its computation is cubic in the number of samples when both distributions have similar sizes, so given the high number of samples identified in our tumour archetypes, we estimated it using the *quantised* Wasserstein distance. It comes down to first reducing each distribution, by taking their k-means centroids as support and assigning them a relative importance which coincides with cluster proportions. Then, factored distributions were compared using their Wasserstein distances, leveraging the implementation of the Python Optimal Transport library^88^. To improve the estimation of the true Wasserstein distances, a high number of 5000 clusters for each distribution was used.

Remark that different hFM representation spaces can have different volumes but similar discernibility between archetypes or clusters (Fig. 4a). Hence, for comparing such spaces in an unbiased manner, the quantised Wasserstein distance was computed on normalised representations. Specifically, we computed the maximal diameter α of the UNI representation space by finding the maximal distance between the two most distant points, and normalised the other embedding spaces by multiplying them by their maximal diameters and dividing by α.

### Hierarchical clustering

Hierarchical clustering between tumour archetypes is computed in the image or gene expression embeddings. In both cases, it is computed using the complete method and the quantised Wasserstein distance, using the *linkage* method from scipy^89^.

### Survival analysis

We modelled time to event (death) using Cox proportional hazards regression, allowing inclusion of compositional covariates representing tumour archetype proportions. To ensure robust estimation, only patients with at least 100 invasive patches were included in the analysis, leaving a total of 60 patients. To address the compositional nature of these data, cluster proportions were first transformed using the centred log-ratio (CLR) transformation, which removes the constant-sum constraint and mitigates spurious correlations. The CLR transformation computes the natural logarithm of each component divided by the geometric mean of all components in the sample, resulting in data constrained to a hyperplane with zero-sum across components. Because the transformed data are highly collinear, standard Cox regression estimation is unstable due to the singularity of the Hessian matrix. To overcome this, we applied ridge penalisation with a penalty parameter of 0.1, which stabilises coefficient estimates while preserving effect directionality. This approach also improves robustness to imbalances in the data, ensuring that the estimated effects are reliable across varying sample distributions. For each patient, the Cox model generated a risk score summarising the effect of tumour composition on the hazard of death. Patients were then ranked by their risk scores, and the top 16 patients, corresponding to the observed number of deaths, were classified as high-risk, with the remaining patients classified as low-risk. Overall survival for these groups was visualised using Kaplan–Meier curves, and differences were assessed using the log-rank test.

### Statistics and reproducibility

All models were evaluated using the Adjusted Rand Index (ARI) and Shannon entropy. Pearson correlation coefficients and associated two-sided p-values were computed to assess the relationship between ARI scores and Shannon entropy. A broad range of UMAP hyperparameters were explored; final parameters were selected based on maximum ARI score when assessing tissue-type discrimination, or by maximising the silhouette score while minimising batch effects when identifying de novo archetypes. Batch effects were systematically assessed to distinguish biological from technical sources of variation. All patients were included in each dataset-specific analysis, with the exception of one patient (patient A, HER2+ dataset) who was excluded from the analysis due to suspected technical artefact. All other patients were included. Normality of distributions was assessed prior to statistical testing; differences between groups were evaluated using two-sided Mann–Whitney U tests for non-normally distributed data and two-sided Pearson’s chi-square tests of independence for categorical variables. A two-sided *p*-value < 0.05 was considered statistically significant. Survival analyses were performed using Cox proportional hazards models with ridge penalisation (L2, λ = 0.1) to address group imbalance. More details about each individual statistical analyses are provided in the Methods. All analyses were conducted to ensure reproducibility: random seeds were fixed for all stochastic procedures including model retraining and clustering. Statistical analyses were conducted in Python (v3.10). No statistical method was used to predetermine sample size. No data were excluded from analyses except where stated. Randomisation and blinding were not applicable to this computational study.

### Reporting summary

Further information on research design is available in the Nature Portfolio Reporting Summary linked to this article.

## Supplementary information

Supplementary Information

Description of Additional Supplementary Files

Supplementary Data 1-8

Reporting Summary

Transparent Peer Review file

## Source data

Source Data

## Data Availability

Processed breast cancer count matrices derived from raw spatial transcriptomics data, along with the corresponding H&E brightfield images, were obtained from Zenodo for the HER2-positive (https://zenodo.org/records/4751624) and TNBC (10.5281/zenodo.8135721) cohorts. Raw Visium HD 3′ spatial transcriptomics data for the HGSOC sample are not deposited, as patient consent for long-term genomic data deposition, required under Swiss law, was not obtained. The processed gene counts and H&E slide, together with the weights of T-UNI models generated in this study have been deposited on Zenodo under accession code 20209534 (10.5281/zenodo.20209534).  Source data are provided with this paper.

## Code availability

The source code is available on Github at: https://github.com/AI-for-RNA-Biology/TumorArchetype_FM and archived on Zenodo at 10.5281/zenodo.20162010^90^.
