# Supplementary material for: Prognostic RNA-splicing archetypes in breast cancer identified by extended pre-training of histopathology foundation models
Source: Nat Commun. 2026 Jul 7;17:8470. doi: 10.1038/s41467-026-75217-z (PMC13478289; doi:10.1038/s41467-026-75217-z)
Supplement: Supplementary file 1 [file 41467_2026_75217_MOESM1_ESM.pdf]

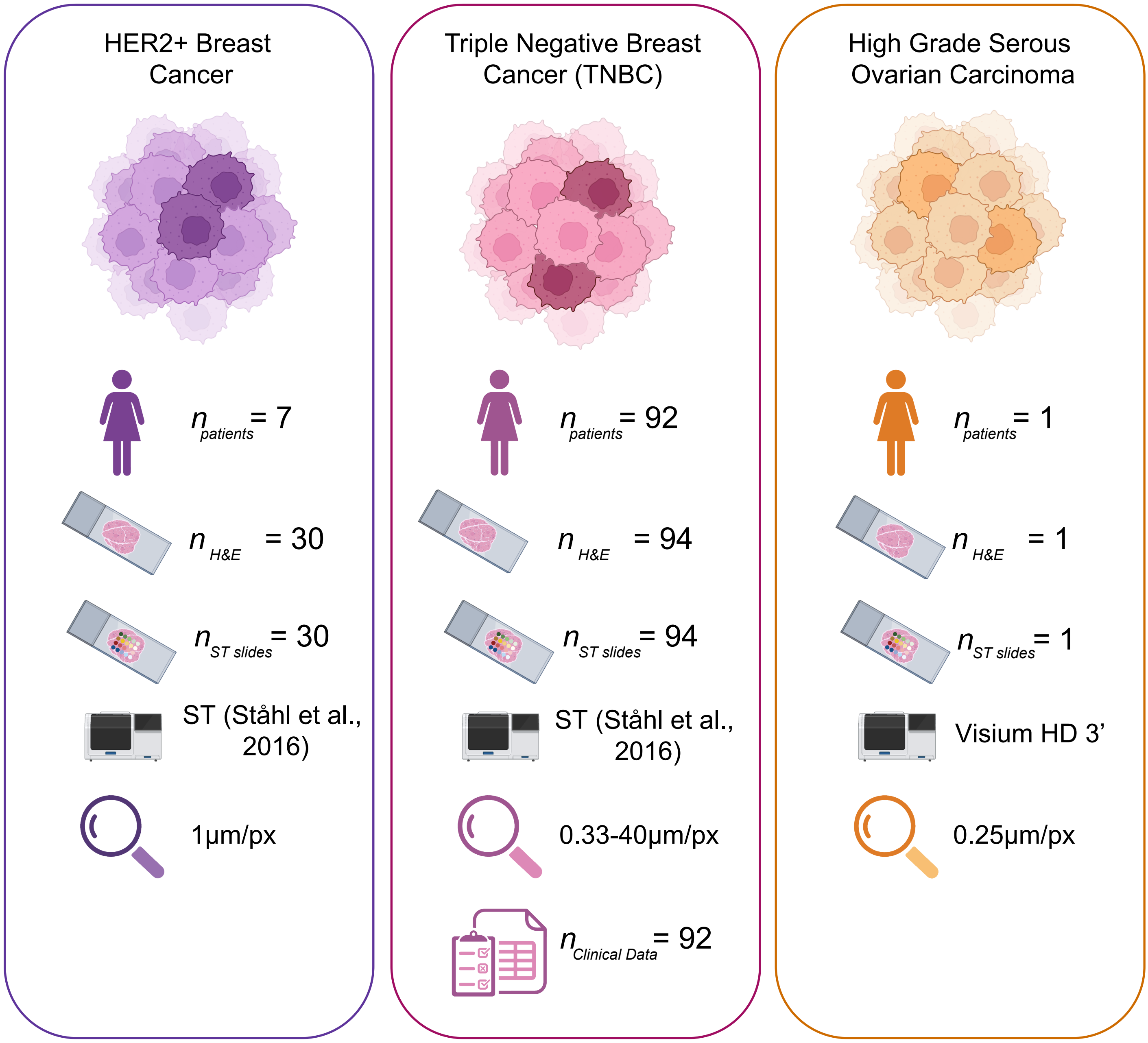

HER2+ Breast Cancer
$n_{patients}$ = 7
$n_{H\&E}$ = 30
$n_{ST\ slides}$ = 30
ST (Ståhl et al., 2016)
1μm/px
Triple Negative Breast Cancer (TNBC)
$n_{patients}$ = 92
$n_{H\&E}$ = 94
$n_{ST\ slides}$ = 94
ST (Ståhl et al., 2016)
0.33-40μm/px
$n_{Clinical\ Data}$ = 92
High Grade Serous Ovarian Carcinoma
$n_{patients}$ = 1
$n_{H\&E}$ = 1
$n_{ST\ slides}$ = 1
Visium HD 3'
0.25μm/px

Supplementary Figure 1 | Overview of the datasets

Schematic overview of the spatial transcriptomics datasets analyzed in this study, including cancer type, number of patients, number of whole-slide images (WSIs), and spatial transcriptomics technology. HER2-positive breast cancer (n = 7) and triple-negative breast cancer (TNBC; n = 92) samples were generated using the original array-based Spatial Transcriptomics (ST) technology[1], whereas the high-grade serous ovarian carcinoma sample was generated using the Visium HD 3′ protocol. Clinical and survival data were available for the TNBC cohort. Illustration partly created in BioRender. Fournier, L. (2026) gl30x3d https://BioRender.com/.

1. Ståhl, P. L. *et al.* Visualization and analysis of gene expression in tissue sections by spatial transcriptomics. *Science* **353**, 78–82 (2016).

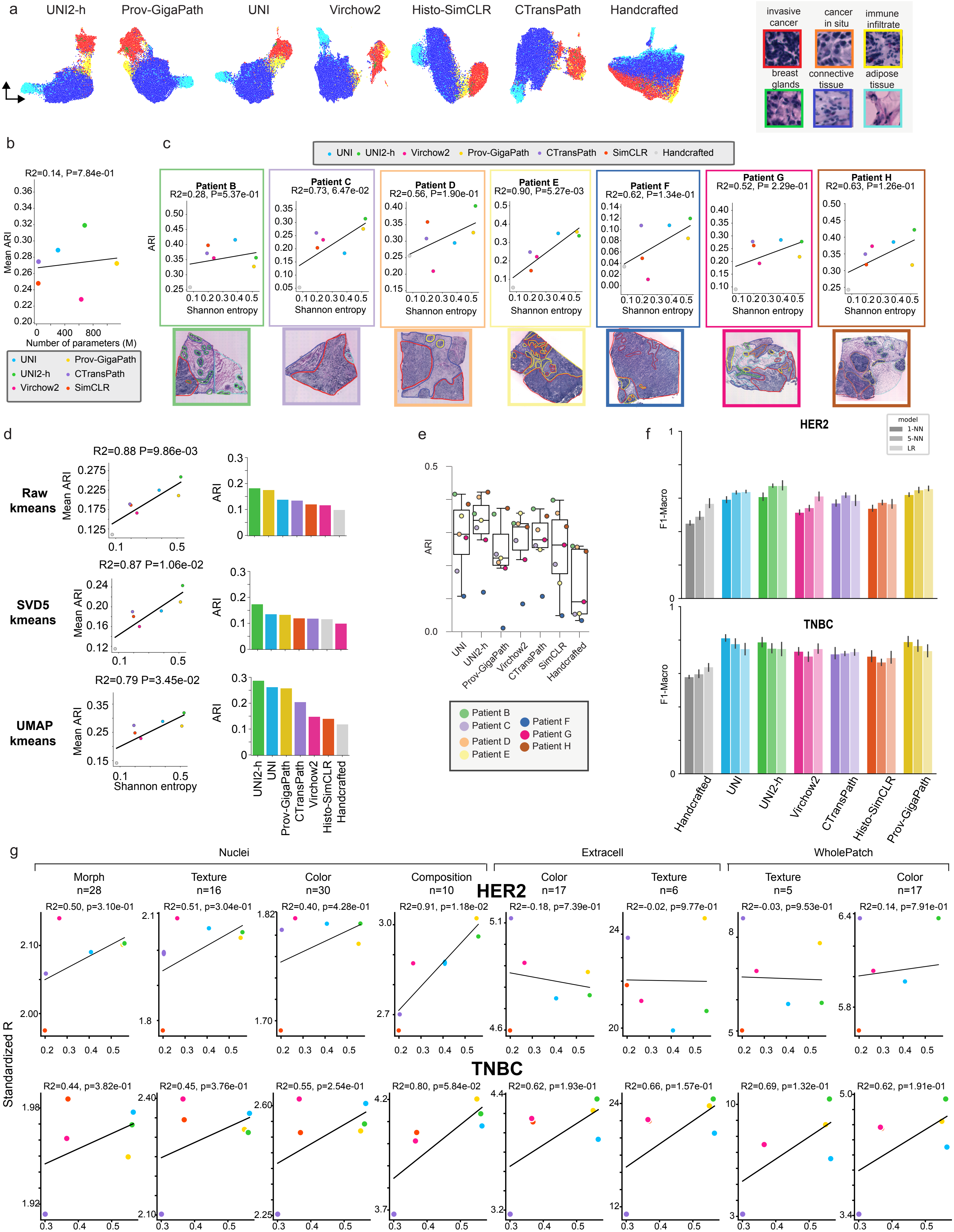

a
UNI2-h
Prov-GigaPath
UNI
Virchow2
Histo-SimCLR
CTransPath
Handcrafted
invasive cancer
cancer in situ
immune infiltrate
breast glands
connective tissue
adipose tissue
b
R2=0.14, P=7.84e-01
Mean ARI
Number of parameters (M)
UNI
UNI2-h
Virchow2
Prov-GigaPath
CTransPath
SimCLR
c
UNI
UNI2-h
Virchow2
Prov-GigaPath
CTransPath
SimCLR
Handcrafted
Patient B
R2=0.28, P=5.37e-01
Patient C
R2=0.73, 6.47e-02
Patient D
R2=0.56, P=1.90e-01
Patient E
R2=0.90, P=5.27e-03
Patient F
R2=0.62, P=1.34e-01
Patient G
R2=0.52, P= 2.29e-01
Patient H
R2=0.63, P=1.26e-01
ARI
Shannon entropy
d
Raw kmeans
R2=0.88 P=9.86e-03
SVD5 kmeans
R2=0.87 P=1.06e-02
UMAP kmeans
R2=0.79 P=3.45e-02
Mean ARI
ARI
Shannon entropy
UNI2-h
UNI
Prov-GigaPath
CTransPath
Virchow2
Histo-SimCLR
Handcrafted
e
ARI
UNI
UNI2-h
Prov-GigaPath
Virchow2
CTransPath
SimCLR
Handcrafted
Patient B
Patient C
Patient D
Patient E
Patient F
Patient G
Patient H
f
HER2
TNBC
model
1-NN
5-NN
LR
F1-Macro
Handcrafted
UNI
UNI2-h
Virchow2
CTransPath
Histo-SimCLR
Prov-GigaPath
g
Nuclei
Extracell
WholePatch
Morph n=28
Texture n=16
Color n=30
Composition n=10
Color n=17
Texture n=6
Texture n=5
Color n=17
HER2
R2=0.50, p=3.10e-01
R2=0.51, p=3.04e-01
R2=0.40, p=4.28e-01
R2=0.91, p=1.18e-02
R2=-0.18, p=7.39e-01
R2=-0.02, p=9.77e-01
R2=-0.03, p=9.53e-01
R2=0.14, p=7.91e-01
TNBC
R2=0.44, p=3.82e-01
R2=0.45, p=3.76e-01
R2=0.55, p=2.54e-01
R2=0.80, p=5.84e-02
R2=0.62, p=1.93e-01
R2=0.66, p=1.57e-01
R2=0.69, p=1.32e-01
R2=0.62, p=1.91e-01
Standardized R

Supplementary Figure 2 | Assessing semantic richness in histopathology representations with entropy

a. UMAP visualizations[38] comparing the patch embeddings of the TNBC dataset ($n_{patches} = 186'554$) from the 6 baseline hFMs and the handcrafted features, colored by tissue type as annotated by the pathologists.
b. Pearson correlation between the number of parameters of the hFMs and the mean tissue-type ARI across patients of the HER2 dataset. R^2 indicates the Pearson correlation coefficient and P the associated two-sided p-value for the null hypothesis of zero correlation.
c. Correlation between SE and tissue-type ARI for each patient of the HER2 dataset for the different hFMs and handcrafted features. R^2 indicates the Pearson correlation coefficient and P the associated two-sided p-value for the null hypothesis of zero correlation. The line marks the linear regression fit. WSIs are shown to illustrate the clustering task.
d. Overall tissue-type ARI score on all HER2 patches for different clustering methods. Upper: k-means is applied on the raw embeddings extracted from the hFMs or handcrafted features. Middle: k-means is applied on the first 5 principal components from singular value decomposition of the raw hFMs embeddings or handcrafted features. Lower: UMAP has been applied to the raw hFMs embeddings and handcrafted features prior to applying k-means. The reported ARI scores are the best scores obtained across all UMAP parameters tested (see *Materials and Methods* section). R^2 indicates the Pearson correlation coefficient and P the associated two-sided p-value for the null hypothesis of zero correlation.
e. Tissue-type ARI scores per HER2 patients for the different hFMs and handcrafted features.
f. Macro F1 scores from 1-NN, 5-NN and logistic-regression classifiers trained on embeddings from all models of the HER2 and TNBC datasets. Tissue-type prediction was evaluated using 5-fold stratified cross-validation with *scikit-learn* default hyperparameters.
g. Scatter plot of the correlation between each handcrafted feature category standardized R^2 (see Methods) and SE for the different hFMs. R^2 indicates the Pearson correlation coefficient and P the associated two-sided p-value for the null hypothesis of zero

correlation. The line represents the linear regression fit. Results are shown for the HER2 and TNBC datasets.

Source data are provided as a Source Data file.

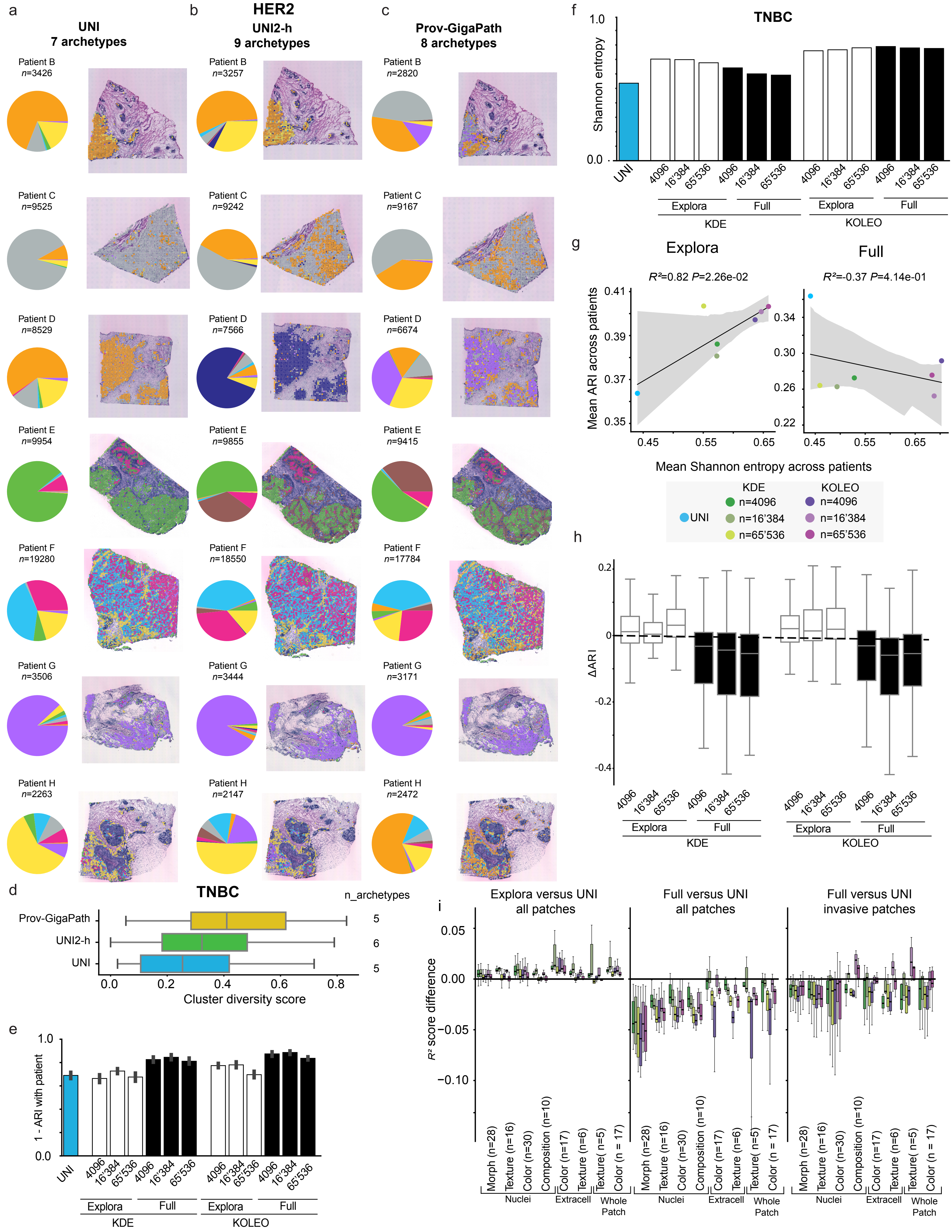

a
b
c
HER2
UNI
7 archetypes
UNI2-h
9 archetypes
Prov-GigaPath
8 archetypes
Patient B
n=3426
n=3257
n=2820
Patient C
n=9525
n=9242
n=9167
Patient D
n=8529
n=7566
n=6674
Patient E
n=9954
n=9855
n=9415
Patient F
n=19280
n=18550
n=17784
Patient G
n=3506
n=3444
n=3171
Patient H
n=2263
n=2147
n=2472
d
TNBC
n_archetypes
Prov-GigaPath
UNI2-h
UNI
5
6
5
0.0
0.2
0.4
0.6
0.8
Cluster diversity score
e
1 - ARI with patient
1.0
0.0
UNI
4096
16'384
65'536
Explora
Full
KDE
KOLEO
f
TNBC
Shannon entropy
g
Explora
Full
R²=0.82 P=2.26e-02
R²=-0.37 P=4.14e-01
Mean ARI across patients
Mean Shannon entropy across patients
KDE
KOLEO
n=4096
n=16'384
n=65'536
h
ΔARI
0.2
0
-0.2
-0.4
i
Explora versus UNI
all patches
Full versus UNI
all patches
Full versus UNI
invasive patches
R² score difference
0.05
0.00
−0.05
−0.10
Morph (n=28)
Texture (n=16)
Color (n=30)
Composition (n=10)
Color (n=17)
Texture (n=6)
Texture(n=5)
Color (n = 17)
Nuclei
Extracell
Whole
Patch

Supplementary Figure 3 | Extended pre-training exclusively with invasive cancer tissue enhances semantic representation on TNBC dataset

a. Pie chart showing the relative abundance of the 7 tumor archetypes identified with UNI on the HER2 dataset. Left: Relative abundance of morphological tumor archetypes per patient. Right: Invasive cancer patches colored according to their tumor archetype in patients' tumor sections.
b. Same as **a** but for UNI2-h.
c. Same as **a** but for Prov-GigaPath.
d. Archetype diversity scores for UNI, UNI2-h, and Prov-GigaPath on the TNBC dataset. For each patient with more than 100 invasive cancer spots (UNI: *n*=69, UNI2-h: *n*=65, Prov-GigaPath: n=68), cluster diversity was quantified using normalised Shannon entropy of the cluster-proportion distribution.
e. Bar graphs of the batch effect mitigation score for UNI and T-UNI models on the TNBC dataset, defined as 1-"*ARI with patient"*, computed using patient labels instead of tissue labels on invasive cancer patches. A high batch effect mitigation score indicates low batch effect.
f. Bar graphs of SE associated with the embeddings from UNI and T-UNI models on the TNBC dataset.
g. Scatter plot of the correlation between mean SE and tissue-type ARI across patients for UNI and T-UNI models using the ExPloRa (left) and full (right) retraining methods on the TNBC dataset. R^2 indicates the Pearson correlation coefficient and P the associated two-sided p-value for the null hypothesis of zero correlation
h. Boxplots of the distribution of ARI score differences per TNBC patient with respect to UNI (baseline) for T-UNI models. ARI per patient quantifies the model's ability to discriminate among tissue types via unsupervised clustering at the patient level.
i. Boxplots of R^2 score differences between T-UNI models and UNI, from linear regression predictions of handcrafted features on the TNBC dataset. Linear regression using all patch embeddings for T-UNI models either trained with the ExPloRa (left) or the full (middle) methods, and using only invasive cancer patch embeddings for T-UNI models trained with full method (right). The number of features per category n is indicated under each plot. Boxplots display the median, the lower and upper quartiles, the minimum and maximum values. Source data are provided as a Source Data file.

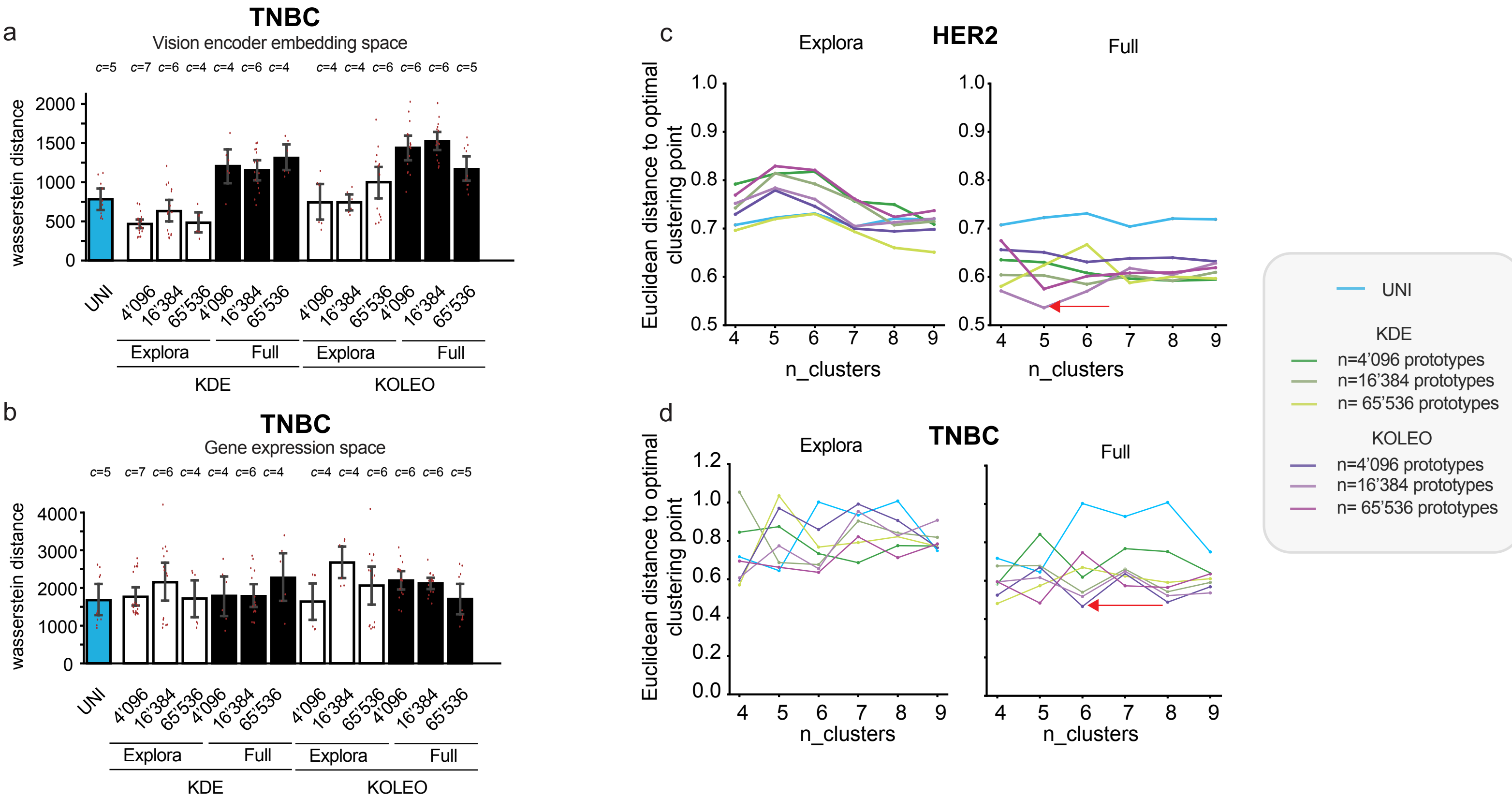
a
TNBC
Vision encoder embedding space
c=5 c=7 c=6 c=4 c=4 c=6 c=4 c=4 c=4 c=6 c=6 c=6 c=5
wasserstein distance
2000
1500
1000
500
0
UNI
4'096
16'384
65'536
Explora
Full
KDE
KOLEO
b
TNBC
Gene expression space
c=5 c=7 c=6 c=4 c=4 c=6 c=4 c=4 c=4 c=6 c=6 c=6 c=5
wasserstein distance
4000
3000
2000
1000
0
c
HER2
Explora
Full
Euclidean distance to optimal clustering point
n_clusters
d
TNBC
Explora
Full
Euclidean distance to optimal clustering point
n_clusters
UNI
KDE
n=4'096 prototypes
n=16'384 prototypes
n= 65'536 prototypes
KOLEO
n=4'096 prototypes
n=16'384 prototypes
n= 65'536 prototypes

Supplementary Figure 4 | Extended pretraining enables the identification of molecularly defined tumour sub-archetypes

a. Bargraphs of the average normalized quantized Wasserstein distances in the UNI and T-UNI embedding spaces between TNBC tumor archetype pairs identified from unsupervised clustering of invasive cancer patches. The number of tumor archetypes identified is indicated at the top for each model. It is selected to maximize the silhouette score while maintaining a high batch-effect mitigation score (**Methods**). Bar height represents the mean (±95% CI) of pairwise quantised Wasserstein distance between clusters.
b. Bargraphs of the average quantized Wasserstein distances in the gene expression space between TNBC tumor archetype pairs identified from unsupervised clustering of invasive cancer patches in the UNI and T-UNI embedding spaces. Bar height represents the mean (±95% CI) of pairwise quantised Wasserstein distance between clusters.
c. Euclidean distance for each value of *k* between the point defined by the silhouette score and batch effect mitigation score, and the optimal point (1, 1). This point represents the situation of perfectly defined clusters (silhouette score = 1) that are also perfectly balanced across patients (batch effect mitigation score = 1). Unsupervised clustering is performed using UMAP-kmeans, selecting the UMAP parameters leading to the best silhouette score for each k for HER2 T-UNI models trained using ExPloRa (left) and using full retraining (right) on the HER2 model. The red arrow shows the model and number of clusters being the closest to the optimal unsupervised clustering.
d. Same as **c** but for the TNBC dataset.

   Source data are provided as a Source Data file.

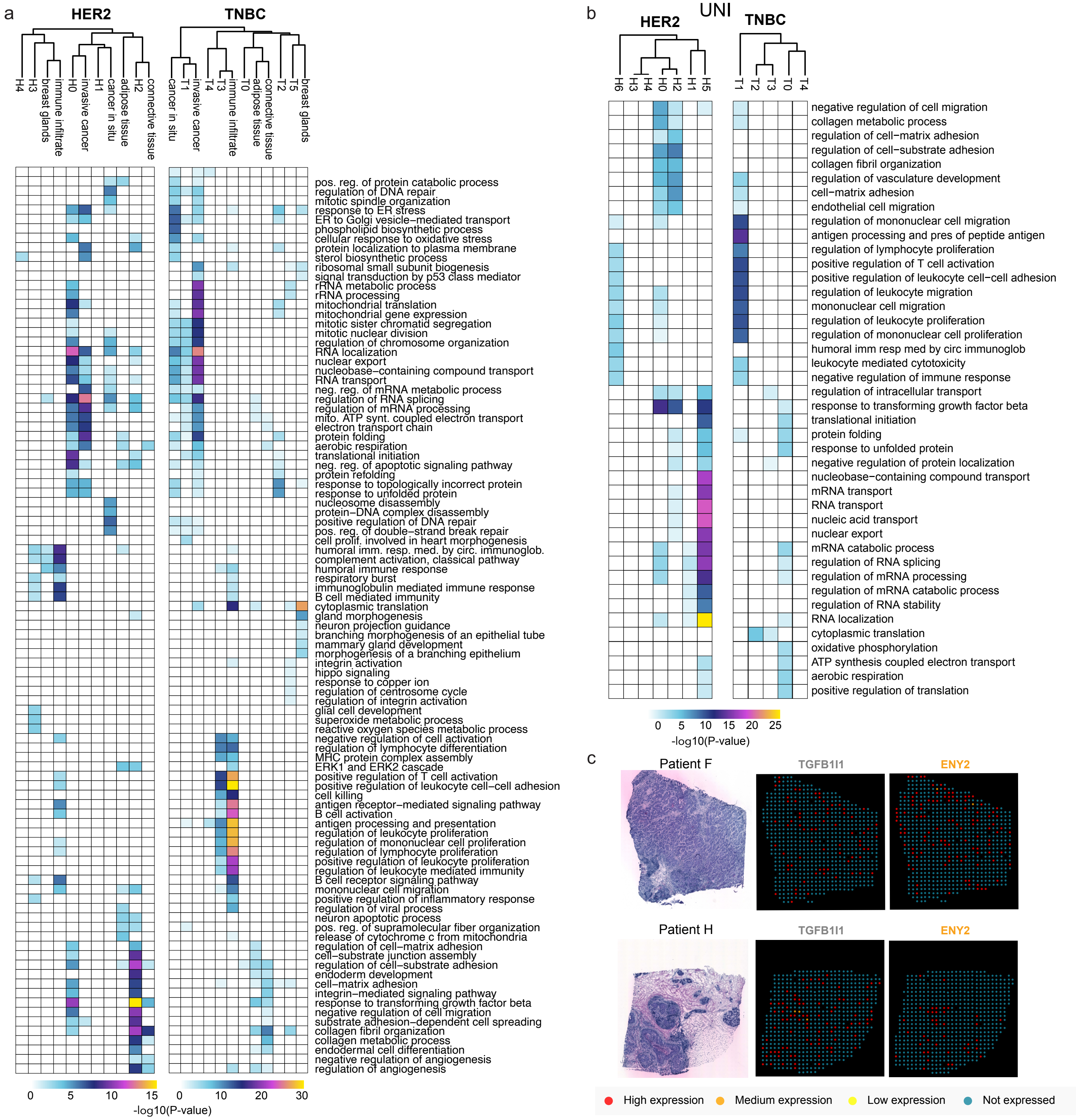

a
HER2
TNBC
H4
H3
breast glands
immune infiltrate
H0
invasive cancer
H1
cancer in situ
adipose tissue
H2
connective tissue
cancer in situ
T1
invasive cancer
T4
T3
immune infiltrate
T0
adipose tissue
connective tissue
T2
T5
breast glands
pos. reg. of protein catabolic process
regulation of DNA repair
mitotic spindle organization
response to ER stress
ER to Golgi vesicle−mediated transport
phospholipid biosynthetic process
cellular response to oxidative stress
protein localization to plasma membrane
sterol biosynthetic process
ribosomal small subunit biogenesis
signal transduction by p53 class mediator
rRNA metabolic process
rRNA processing
mitochondrial translation
mitochondrial gene expression
mitotic sister chromatid segregation
mitotic nuclear division
regulation of chromosome organization
RNA localization
nuclear export
nucleobase−containing compound transport
RNA transport
neg. reg. of mRNA metabolic process
regulation of RNA splicing
regulation of mRNA processing
mito. ATP synt. coupled electron transport
electron transport chain
protein folding
aerobic respiration
translational initiation
neg. reg. of apoptotic signaling pathway
protein refolding
response to topologically incorrect protein
response to unfolded protein
nucleosome disassembly
protein−DNA complex disassembly
positive regulation of DNA repair
pos. reg. of double−strand break repair
cell prolif. involved in heart morphogenesis
humoral imm. resp. med. by circ. immunoglob.
complement activation, classical pathway
humoral immune response
respiratory burst
immunoglobulin mediated immune response
B cell mediated immunity
cytoplasmic translation
gland morphogenesis
neuron projection guidance
branching morphogenesis of an epithelial tube
mammary gland development
morphogenesis of a branching epithelium
integrin activation
hippo signaling
response to copper ion
regulation of centrosome cycle
regulation of integrin activation
glial cell development
superoxide metabolic process
reactive oxygen species metabolic process
negative regulation of cell activation
regulation of lymphocyte differentiation
MHC protein complex assembly
ERK1 and ERK2 cascade
positive regulation of T cell activation
positive regulation of leukocyte cell−cell adhesion
cell killing
antigen receptor−mediated signaling pathway
B cell activation
antigen processing and presentation
regulation of leukocyte proliferation
regulation of mononuclear cell proliferation
regulation of lymphocyte proliferation
positive regulation of leukocyte proliferation
regulation of leukocyte mediated immunity
B cell receptor signaling pathway
mononuclear cell migration
positive regulation of inflammatory response
regulation of viral process
neuron apoptotic process
pos. reg. of supramolecular fiber organization
release of cytochrome c from mitochondria
regulation of cell−matrix adhesion
cell−substrate junction assembly
regulation of cell−substrate adhesion
endoderm development
cell−matrix adhesion
integrin−mediated signaling pathway
response to transforming growth factor beta
negative regulation of cell migration
substrate adhesion−dependent cell spreading
collagen fibril organization
collagen metabolic process
endodermal cell differentiation
negative regulation of angiogenesis
regulation of angiogenesis
0 5 10 15
0 10 20 30
-log10(P-value)
b
UNI
HER2
TNBC
H6
H3
H4
H0
H2
H1
H5
T1
T2
T3
T0
T4
negative regulation of cell migration
collagen metabolic process
regulation of cell−matrix adhesion
regulation of cell−substrate adhesion
collagen fibril organization
regulation of vasculature development
cell−matrix adhesion
endothelial cell migration
regulation of mononuclear cell migration
antigen processing and pres of peptide antigen
regulation of lymphocyte proliferation
positive regulation of T cell activation
positive regulation of leukocyte cell−cell adhesion
regulation of leukocyte migration
mononuclear cell migration
regulation of leukocyte proliferation
regulation of mononuclear cell proliferation
humoral imm resp med by circ immunoglob
leukocyte mediated cytotoxicity
negative regulation of immune response
regulation of intracellular transport
response to transforming growth factor beta
translational initiation
protein folding
response to unfolded protein
negative regulation of protein localization
nucleobase−containing compound transport
mRNA transport
RNA transport
nucleic acid transport
nuclear export
mRNA catabolic process
regulation of RNA splicing
regulation of mRNA processing
regulation of mRNA catabolic process
regulation of RNA stability
RNA localization
cytoplasmic translation
oxidative phosphorylation
ATP synthesis coupled electron transport
aerobic respiration
positive regulation of translation
0 5 10 15 20 25
-log10(P-value)
c
Patient F
TGFB1I1
ENY2
Patient H
TGFB1I1
ENY2
High expression
Medium expression
Low expression
Not expressed

Supplementary Figure 5 | Histopathology-derived tumour archetypes reveal distinct molecular pathways in invasive breast cancer

a. Heatmap of the biological pathway enrichment analysis of differentially expressed genes (using MAST[84]; log2FC>0.25 and P-value<0.01) between the different tissue types annotated by pathologists, as well as tumor archetypes identified with the best T-UNI for each dataset (full training strategy, KoLeo loss, 16K prototypes for HER2 and 4096 prototypes for TNBC). The -log10 pvalue is reported for the top 10 significantly enriched pathways in each tumor archetype or tissue type, provided they contain at least 5 of the identified marker genes. Redundant pathways have been curated manually. Hierarchical clustering is performed using 1-pearson correlation as the distance metric, and the complete method from *hclust* function in the *stats*[91] R package. Pathway enrichment was assessed using g:Profiler (gprofiler2) with a one-sided hypergeometric test against the GO Biological Process database, with family-wise error rate controlled via a permutation-based threshold.

b. Heatmap of the biological pathway enrichment analysis of differentially expressed genes (using MAST[84]; log2FC>0.25 and P-value<0.01) between the tumor archetypes identified with UNI. The -log10 p-value is reported for the top 10 significantly enriched pathways in each tumor archetype, provided that each pathway contains at least 5 of the identified marker genes. Redundant pathways have been curated manually. Hierarchical clustering is performed using 1-pearson correlation as the distance metric, and the complete method from *hclust* function in the *stats*[91] R package.

c. Whole slide image of patients F (upper) and H (lower) from HER2 dataset with their spot expression of TGB1I1 and ENY2 involved in TGF-β and RNA splicing pathways, respectively. The distribution of the given gene's expression was analyzed in log2 scale. The mean and standard deviation (std) were calculated. The expression levels of each spot were then categorized as follows: Not expressed if $log_2(count + 1) = 0$; Low expression if $log_2(count + 1) < mean - std$; Medium expression if $mean - std < log_2(count + 1) < mean + std$; High expression if $log_2(count + 1) > mean + std$, with $count$ being the raw count of the spot for the given gene.

Source data are provided as a Source Data file.

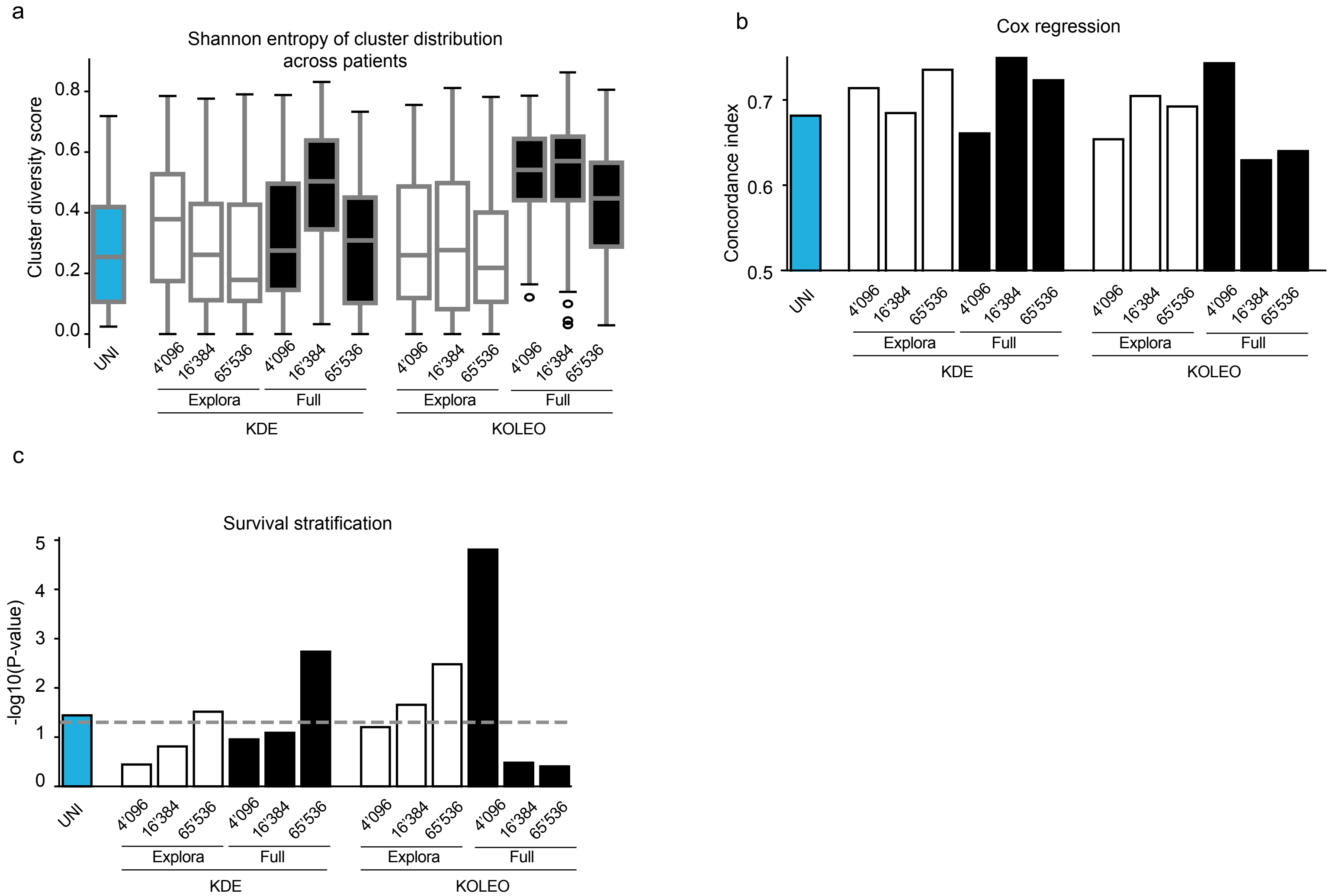
a
Shannon entropy of cluster distribution across patients
Cluster diversity score
0.0
0.2
0.4
0.6
0.8
UNI
4'096
16'384
65'536
4'096
16'384
65'536
Explora
Full
KDE
4'096
16'384
65'536
4'096
16'384
65'536
Explora
Full
KOLEO
b
Cox regression
Concordance index
0.5
0.6
0.7
UNI
4'096
16'384
65'536
4'096
16'384
65'536
Explora
Full
KDE
4'096
16'384
65'536
4'096
16'384
65'536
Explora
Full
KOLEO
c
Survival stratification
-log10(P-value)
0
1
2
3
4
5
UNI
4'096
16'384
65'536
4'096
16'384
65'536
Explora
Full
KDE
4'096
16'384
65'536
4'096
16'384
65'536
Explora
Full
KOLEO

Supplementary Figure 6 | RNA splicing as a prognostic biomarker in breast cancer with potential to generalize across epithelial tumors.

a. Boxplots showing the distribution of archetype diversity scores across patients for UNI and all T-UNI models on the TNBC dataset. For each patient, diversity was quantified using normalized Shannon entropy of the cluster-proportion distribution (range 0–1), where 1 indicates equal representation of all archetypes and 0 indicates dominance by a single archetype.

b. Bar plot showing the concordance index (C-index) for Cox models fitted with archetype proportions derived from UNI and all T-UNI models. The C-index reflects the model's ability to correctly rank patients by survival risk (1.0 = perfect concordance; 0.5 = no better than random).

c. Bar plots showing the $-\log_{10}$ P-values from log-rank tests comparing high- and low-risk patient groups stratified by Cox-model risk scores. Risk scores were derived from Cox models fitted with cluster proportions for each model (UNI and all T-UNI models). The high-risk group includes the 16 patients with the highest predicted risk scores, matching the number of observed deaths in the dataset. Higher bars indicate stronger evidence for differences in survival between risk groups.

Source data are provided as a Source Data file.

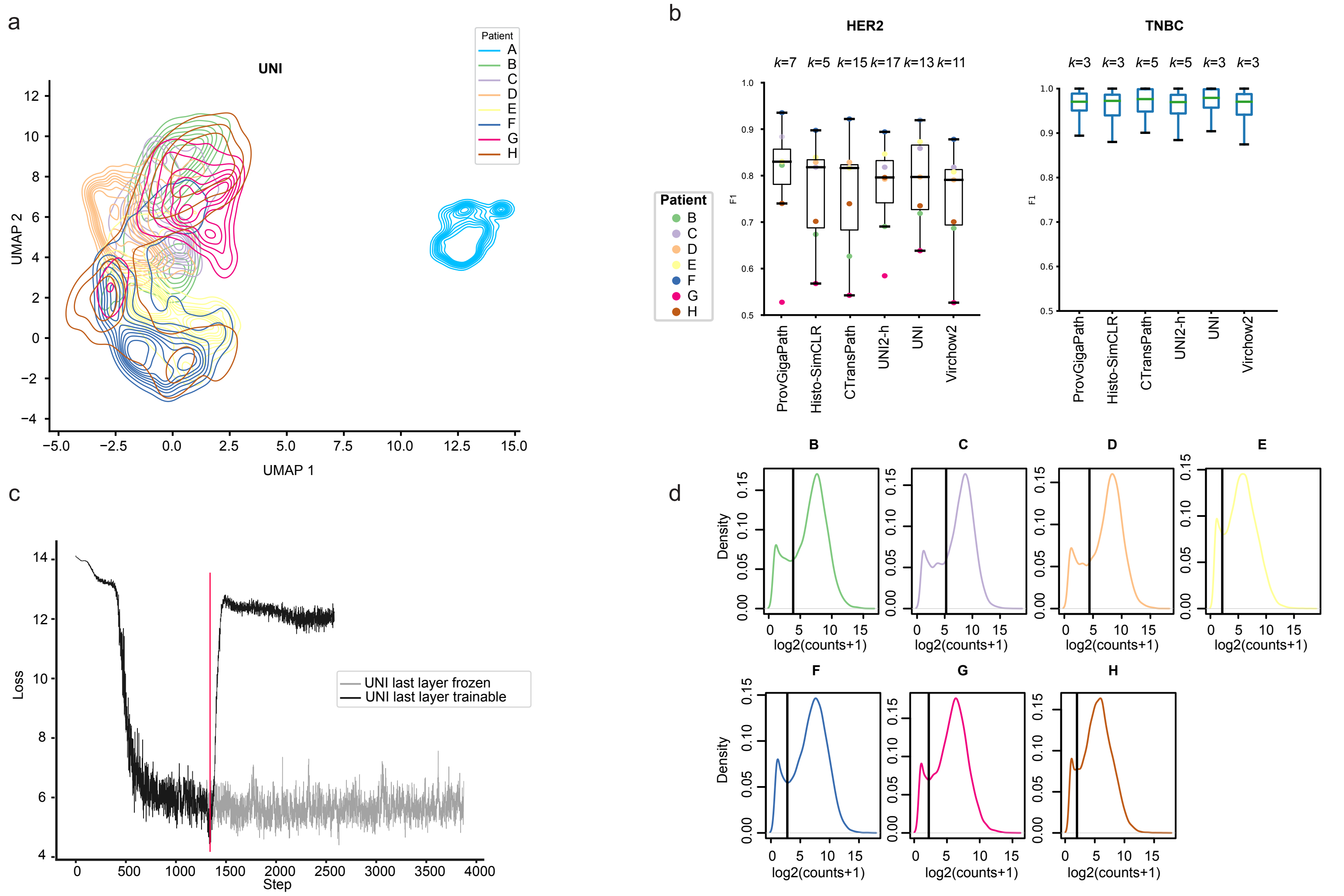
a
UNI
Patient
A
B
C
D
E
F
G
H
UMAP 1
UMAP 2
b
HER2
TNBC
k=7 k=5 k=15 k=17 k=13 k=11
k=3 k=3 k=5 k=5 k=3 k=3
Patient
B
C
D
E
F
G
H
F1
ProvGigaPath
Histo-SimCLR
CTransPath
UNI2-h
UNI
Virchow2
c
Loss
Step
UNI last layer frozen
UNI last layer trainable
d
B
C
D
E
F
G
H
Density
log2(counts+1)

Supplementary Figure 7 | Quality control and methodological assessments for histopathology patch embedding, k-NN relabeling, extended pre-training, and spatial transcriptomics gene filtering

- **a.** **a** UMAP projections[38] of all HER2 patches ($n_{patches} = 103023$) embedding extracted from UNI, showing patient A as an outlier in the data.
- b. F1-score per patient after k-NN relabeling of patches per patient, performed on the different hFMs. The optimal k for each model has been selected based on f1-score optimization and is reported at the top of the boxplots (values ranging from 3 to 21 with a step of 2 have been tested). (Left) HER2 dataset, n=7 patients. (Right) TNBC dataset, n=96 patients.
- **c.** DinoV2 total loss when performing extended pre-training with invasive cancer patches. Two different set-ups are shown: the last layer is trainable (black) and the last layer is frozen (gray).
- **d.** Identification of reliably expressed genes per patient, involving creating a pseudo-bulk expression vector for each patient from the ST expression data, fitting a bimodal distribution to the log2-transformed gene counts, and excluding 99% of background noise (threshold indicated by the black vertical line). This is shown for the 7 patients of the HER2 datasets.

  Source data are provided as a Source Data file.
