# Supplementary material for: Prognostic RNA-splicing archetypes in breast cancer identified by extended pre-training of histopathology foundation models
Source: Nat Commun. 2026 Jul 7;17:8470. doi: 10.1038/s41467-026-75217-z (PMC13478289; doi:10.1038/s41467-026-75217-z)
Supplement: Supplementary file 2 [file 41467_2026_75217_MOESM2_ESM.pdf]

Supplementary Data 1: Histopathology foundation models (hFMs) descriptions (benchmark tasks, training data, model architecture).

Supplementary Data 2: List and description of handcrafted features computed after CellViT[26] segmentation.

Supplementary Data 3: List and parameters of the extended pre-trained UNI (T-UNI) models. All HER2 T-UNI models were pre-trained on the same HER2 invasive cancer patches dataset, and all TNBC T-UNI models on the same TNBC invasive cancer patches dataset (see *Materials and Methods*).

Supplementary Data 4: Model performances on the HER2[36] and TNBC[37] datasets for the 6 baseline hFMs and the 12 T-UNI models.

Supplementary Data 5: Description of the patch dataset created from the HER2 and TNBC datasets[36,37], with number of patches and labeled spots per tissue type and per patient.

Supplementary Data 6: k-NN and UMAP–k-means parameters used for tumor archetype clustering, for the 12 T-UNI models of each dataset (HER2 and TNBC). The optimal k for labeling invasive patches using k-NN was selected to maximize the median F1-score across patients, testing values from 3 to 21 in increments of 2. UMAP parameters (minimum distance and number of neighbors) were first optimized for each cluster number *k* ranging from 4 to 9 to maximize the silhouette score. The final *k* for k-means clustering was then selected to jointly maximize the silhouette score while minimizing batch effects, based on the previously identified optimal UMAP configurations for each *k*.

Supplementary Data 7: Biological pathway enrichment analysis of differentially expressed genes (using MAST[84]; log2FC > 0.25 and P-value < 0.01) between pathologist-annotated tissue types and tumor archetypes identified by the best T-UNI model for each dataset (full training strategy, KoLeo loss, 16K prototypes for HER2, and 4,096 prototypes for TNBC). The –log10 P-value is reported for all significantly enriched pathways containing at least five of the identified marker genes. This table shows the full, uncurated list.

Supplementary Data 8: Same as Table S7 for the tumor archetypes identified using UNI.
