# Supplementary material for: Prognostic RNA-splicing archetypes in breast cancer identified by extended pre-training of histopathology foundation models
Source: Nat Commun. 2026 Jul 7;17:8470. doi: 10.1038/s41467-026-75217-z (PMC13478289; doi:10.1038/s41467-026-75217-z)
Supplement: Supplementary file 3 [file 41467_2026_75217_MOESM4_ESM.pdf]

nature portfolio

Corresponding author(s): Cédric Vincent-Cuaz & Raphaëlle Luisier

Last updated by author(s): May 18, 2026

Reporting Summary

Nature Portfolio wishes to improve the reproducibility of the work that we publish. This form provides structure for consistency and transparency in reporting. For further information on Nature Portfolio policies, see our Editorial Policies and the Editorial Policy Checklist.

Statistics

For all statistical analyses, confirm that the following items are present in the figure legend, table legend, main text, or Methods section.

n/a	Confirmed	
☐	☒	The exact sample size (*n*) for each experimental group/condition, given as a discrete number and unit of measurement
☐	☒	A statement on whether measurements were taken from distinct samples or whether the same sample was measured repeatedly
☐	☒	The statistical test(s) used AND whether they are one- or two-sided<br>*Only common tests should be described solely by name; describe more complex techniques in the Methods section.*
☒	☐	A description of all covariates tested
☐	☒	A description of any assumptions or corrections, such as tests of normality and adjustment for multiple comparisons
☐	☒	A full description of the statistical parameters including central tendency (e.g. means) or other basic estimates (e.g. regression coefficient) AND variation (e.g. standard deviation) or associated estimates of uncertainty (e.g. confidence intervals)
☐	☒	For null hypothesis testing, the test statistic (e.g. *F*, *t*, *r*) with confidence intervals, effect sizes, degrees of freedom and *P* value noted<br>*Give P values as exact values whenever suitable.*
☒	☐	For Bayesian analysis, information on the choice of priors and Markov chain Monte Carlo settings
☐	☒	For hierarchical and complex designs, identification of the appropriate level for tests and full reporting of outcomes
☐	☒	Estimates of effect sizes (e.g. Cohen's *d*, Pearson's *r*), indicating how they were calculated

Our web collection on statistics for biologists contains articles on many of the points above.

Software and code

Policy information about availability of computer code

Data collection	SpaceRanger was used to process the Visium HD 3' sample.
Data analysis	10.5281/zenodo.20162010

For manuscripts utilizing custom algorithms or software that are central to the research but not yet described in published literature, software must be made available to editors and reviewers. We strongly encourage code deposition in a community repository (e.g. GitHub). See the Nature Portfolio guidelines for submitting code & software for further information.

Data

Policy information about availability of data

All manuscripts must include a data availability statement. This statement should provide the following information, where applicable:

- Accession codes, unique identifiers, or web links for publicly available datasets
- A description of any restrictions on data availability
- For clinical datasets or third party data, please ensure that the statement adheres to our policy

Processed breast cancer count matrices derived from raw spatial transcriptomics data, along with the corresponding H&E brightfield images, were obtained from Zenodo for the HER2-positive (https://zenodo.org/records/4751624) and TNBC (https://doi.org/10.5281/zenodo.8135721) cohorts. Raw Visium HD 3’ spatial transcriptomics data for the HGSOC sample are not deposited, as the patient's informed consent did not cover long-term deposition of genomic data in a repository, in accordance with Swiss data protection requirements. The processed gene counts and H&E slide, together with the weights of T-UNI models generated in this study have been deposited on Zenodo under accession code 20209534 (10.5281/zenodo.20209534). Source data are provided with this paper.

Research involving human participants, their data, or biological material

Policy information about studies with human participants or human data. See also policy information about sex, gender (identity/presentation), and sexual orientation and race, ethnicity and racism.

Reporting on sex and gender	Sex and gender were not considered as variables in the study design, as the primary datasets consist exclusively of female patients (breast and ovarian cancer cohorts), precluding sex-based comparisons.
Reporting on race, ethnicity, or other socially relevant groupings	The HGSOC dataset contains only one sample so we cannot report any relevant groupings.
Population characteristics	The HGSOC dataset contains only one sample so we cannot report any characteristics at the population level.
Recruitment	The sample was collected from a patient enrolled in the Nab-PIPAC trial (NCT04000906). The sample was collected after the patient was enrolled in the trial. The patient was diagnosed with heavily pretreated platinum-resistant ovarian cancer prior to the enrolment in the trial.
Ethics oversight	The ovarian cancer sample was collected from a patient enrolled in the Nab-PIPAC trial (NCT04000906). The study was approved by local ethical committee (CCER 2024-01382) and was conducted in accordance with the Principles of Good Clinical Practice and the Declaration of Helsinki. The patient provided written informed consent.

Note that full information on the approval of the study protocol must also be provided in the manuscript.

Field-specific reporting

Please select the one below that is the best fit for your research. If you are not sure, read the appropriate sections before making your selection.

[x] Life sciences [ ] Behavioural & social sciences [ ] Ecological, evolutionary & environmental sciences

For a reference copy of the document with all sections, see nature.com/documents/nr-reporting-summary-flat.pdf

Life sciences study design

All studies must disclose on these points even when the disclosure is negative.

Sample size	For the HER2+ and TNBC datasets, please refer to the original dataset. For the HGSOC dataset, only one sample has been selected due to the cost of sequencing and for the purpose of preliminary analyses only.
Data exclusions	For the HER2+ dataset, patient A has been excluded from the analysis because the corresponding images showed suspected technical artefacts. For the other datasets, all samples were included.
Replication	To ensure reproducibility of the findings, originally derived from the HER2+ dataset, all experiments have been replicated on the TNBC datasets, validating the results. On the computational point on view, multiple people from the team have re-run the code individually to ensure code reproducibility.
Randomization	Randomisation was not applicable to this study. All analyses consist of unsupervised computational methods applied to entire patient cohorts without prior group allocation. Clinical and demographic covariates were not used to assign patients to groups; cluster assignments and risk stratifications emerged entirely from the data.
Blinding	Blinding was not applicable to this study. All analyses are fully computational and unsupervised, with no experimental intervention or outcome assessment performed by investigators that could be subject to allocation bias. Cluster labels and risk group assignments were derived directly from the data without investigator input.

Reporting for specific materials, systems and methods

We require information from authors about some types of materials, experimental systems and methods used in many studies. Here, indicate whether each material, system or method listed is relevant to your study. If you are not sure if a list item applies to your research, read the appropriate section before selecting a response.

Materials & experimental systems

n/a	Involved in the study	
☒	☐	Antibodies
☒	☐	Eukaryotic cell lines
☒	☐	Palaeontology and archaeology
☒	☐	Animals and other organisms
☐	☒	Clinical data
☒	☐	Dual use research of concern
☒	☐	Plants

Methods

n/a	Involved in the study	
☒	☐	ChIP-seq
☒	☐	Flow cytometry
☒	☐	MRI-based neuroimaging

Clinical data

Policy information about clinical studies

All manuscripts should comply with the ICMJE guidelines for publication of clinical research and a completed CONSORT checklist must be included with all submissions.

Clinical trial registration	The ovarian cancer sample was collected from a patient enrolled in the Nab-PIPAC trial (NCT04000906)
Study protocol	The study protocol has been published in BMJ open: https://bmjopen.bmj.com/content/13/1/e067691
Data collection	the data were collected on securetrial.com, in accordance with GCP regulations. Patients were enrolled between September 2021 and November 2023.
Outcomes	The primary endpoint was to assess safety and tolerability of cisplatin and nab-paclitaxel administered by PIPAC and to determine the maximum tolerated dose (MTD) of nab-paclitaxel. Secondary endpoints included i) pharmacokinetic analyses of free plasmatic concentrations of nab-paclitaxel ; ii) evaluate histological regression assessed according to PRGS ; iii) assess the objective response rate (ORR) and the clinical benefit rate (CBR), defined by revised RECIST version 1.1 criteria ; iv) to evaluate any benefit in quality of life assessed by EORTC QLQ-C30 v3.0 and visual analogic scale for pain questionnaires.

Plants

Seed stocks	*Report on the source of all seed stocks or other plant material used. If applicable, state the seed stock centre and catalogue number. If plant specimens were collected from the field, describe the collection location, date and sampling procedures.*
Novel plant genotypes	*Describe the methods by which all novel plant genotypes were produced. This includes those generated by transgenic approaches, gene editing, chemical/radiation-based mutagenesis and hybridization. For transgenic lines, describe the transformation method, the number of independent lines analyzed and the generation upon which experiments were performed. For gene-edited lines, describe the editor used, the endogenous sequence targeted for editing, the targeting guide RNA sequence (if applicable) and how the editor was applied.*
Authentication	*Describe any authentication procedures for each seed stock used or novel genotype generated. Describe any experiments used to assess the effect of a mutation and, where applicable, how potential secondary effects (e.g. second site T-DNA insertions, mosiacism, off-target gene editing) were examined.*
