# Supplementary material for: Prognostic RNA-splicing archetypes in breast cancer identified by extended pre-training of histopathology foundation models
Source: Nat Commun. 2026 Jul 7;17:8470. doi: 10.1038/s41467-026-75217-z (PMC13478289; doi:10.1038/s41467-026-75217-z)
Supplement: Supplementary file 4 [file 41467_2026_75217_MOESM5_ESM.pdf]

nature portfolio

Peer Review File

Prognostic RNA-splicing archetypes in breast cancer identified by extended pre-training of histopathology foundation models

Corresponding Author: Professor Raphaelle Luisier

This file contains all reviewer reports in order by version, followed by all author rebuttals in order by version.

Version 0:

Reviewer comments:

Reviewer #1

(Remarks to the Author)
The aim of the work is to characterize intratumor heterogeneity in breast cancer. To achieve this, the authors propose specializing foundation models by extending pre-training. They then use molecular data (spatial transcriptomics) to evaluate the clustering learned via imaging-only. The primary components of the analysis are: (1) systematic evaluations of biological concepts in foundation models (2) evaluation of extended pre-training approaches and (3) characterization of the molecular signatures.

Overall, I think an investigation of how well hFMs capture biology is of interest to the community. I thought the description of investigations into extended pre-training and the biological analysis were nicely done. However I have some questions before recommending this work for publication.

1. The dataset is described as using 100 x 100 pixel patches from the whole slide image, however most hFMs don't allow for inputs of this size. Could the authors describe the pre-processing performed? Given that the center-to-center distance is 200 microns, did the authors consider generating image patches at a resolution closer to the training data distribution (i.e. 256 x 256 px)?

2. My understanding is that Shannon entropy is calculated for all samples and the ARI is calculated for each patient. Is this correct? Could the authors explain this choice if so?

3. I'd like to understand better the choice to only do extended training on invasive cancer, as well as the choice to use the same dataset for extended pertaining and analysis. In the discussion, the authors note that they provide a data-driven alternative to manual annotation, however, it seemed to me that the approach required the invasive cancer annotation to be successful. Could the model have been specialized without these annotations? And could a different cohort of breast cancer data have been used to achieve the specialization?

4. Did the authors consider defining archetypes at a level greater than the tile? The strong patient effects seems somewhat surprising given generalization results in the literature. Could it be that at small resolution patient effects dominate over TME characterization?

5. Did the authors consider applying stain normalizing techniques to pre-process the data and observe whether or not patient differences still dominate the clustering?

The core open question I have is whether or not the authors are arguing that hFMs must be specialized to particular cohorts, i.e. extend pre-training using the cohort of interest, or if extended pre-training is more general, e.g. specializing for breast cancer.

Small comments:
1. I found the authors overused the word "comprehensive". Please considering removing or revising.
2. I found referring to the Ciga et al. model as "SimCLR" confusing as I thought at first it was a natural image model. Perhaps

the authors could call it something else?
3. I don’t think it’s fair to say that hFMs have architectural diversity when almost all use transformers. Please consider revising (line 126)
4. Could Figure3f be edited to have a consistent y-axis? I think that would help demonstrate the result.
5. The authors commented that they could consider multi-modal models with IHC in future work, but Virchow2 is already multi-modal with respect to IHC.

Reviewer #3

(Remarks to the Author)
The paper presents a compelling and thorough investigation into the potential of histopathology foundation models (hFMs) for identifying molecularly distinct tumor regions within invasive breast cancer using routine H&E-stained slides. The study successfully extends the use of these models through advanced pretraining techniques and highlights their ability to uncover tumor heterogeneity that would otherwise be undetected by conventional methods. The integration of molecular data from spatial transcriptomics with AI-driven histopathology analysis is a significant contribution to the field and could pave the way for more effective, precision-based cancer diagnostics. Here are some concerns.

1. Details of the ExPLoRa Method: The description of the training strategy and hyperparameter settings is not sufficiently clear. It is recommended to include a table of hyperparameters in the Supplementary section and briefly explain the optimization strategy. In the Methods section, please briefly discuss the relationship between model capacity and data complexity, and provide the rationale for the selection of hyperparameters.

2. The results are based solely on HER2+ breast cancer data, with limited external validation. It is recommended to supplement the study with external datasets to validate the model’s adaptability across different subtypes. Alternatively, please indicate plans to include such external data for future validation.

3. The comparison of prediction differences between UNI and T-UNI in specific regions is not sufficiently intuitive. It is recommended to include a comparative figure that clearly demonstrates the differences in the recognition capabilities of the two models in typical tissue regions.

4. The value of tumor subtypes has not been evaluated in relation to prognosis or treatment response. If the data is available, it is recommended to include Kaplan-Meier curves or other survival analyses. If not, please consider discussing potential research directions in the discussion section.

5. The paper has not explored whether the method can be extended to other diseases or tissues. It is recommended to add a discussion in the Discussion section regarding the model's generalizability and potential applications across different diseases.

6. Although dysregulation of RNA splicing and TGF-β pathways has been identified, the paper does not delve into how these pathways drive tumor heterogeneity or affect treatment response. It is recommended to further elaborate on these pathways in the context of known breast cancer mechanisms. While several relevant studies are cited, the Results section does not explicitly compare the tumor archetypes identified in this study with existing breast cancer subtypes. It is suggested to reference current molecular subtypes when discussing patient-specific archetypes to strengthen the contextual support for the results.

7. Page 8 mentions that full training leads to a decrease in ARI scores, possibly due to the loss of previously encoded biological concepts. It is recommended to more clearly explain the mechanism of this "catastrophic forgetting" and discuss how future training strategies can be optimized to balance new and old knowledge.

8. Page 18 mentions that the UMAP-k-means clustering parameters are optimized for the best ARI score, but the optimization process (e.g., parameter range or algorithm) is not specified. It is recommended to provide a detailed hyperparameter selection process in the supplementary materials.

9. Pages 15-16 describe 153 handcrafted features (such as nuclear morphology, texture, color, etc.), but do not discuss whether these features comprehensively cover the biological information in H&E images. It is recommended to analyze the representativeness of these handcrafted features or perform a more in-depth comparison with deep learning features.

10. Although Combat correction is mentioned, its impact on the gene expression space is not clearly explained. It is recommended to show the UMAP projection differences of the gene expression space before and after correction in the Results section to validate the correction effect.

11 . If possible, it is recommended to include more patients.

Reviewer #4

(Remarks to the Author)
In this manuscript, Fournier et al. systematically evaluated the biological features extracted by several histopathology foundation models and proposed an extended pre-training approach on invasive tumor patches to specialize the foundation model to enhance the model's ability to capture tumor-specific biological information. Using this approach, they reported two co-existing tumor archetypes linked to RNA splicing and TGF-β dysregulation, respectively.

While the study is timely and potential impactful, several limitations should be addressed:

Major comments:
1. External validation and cohort size: The core analyses are performed on the HER2-positive breast cancer dataset (36 WSIs, 8 patients; patient A excluded; downstream analyses use 7 patients), without an external validation cohort. This raises concerns about overfitting and generalizability.

3. Robustness: Quantitative measures such as Shannon entropy (SE) and Adjusted Rand Index (ARI) were reported at patient-level. Given there are only 7 patients, it's hard to judge the results, for example the correlation between SE and ARI across the 7 points. This could be partially improved by demonstrated this results at slide-level as they have approximately 30 slides available.

3. Color variation and stain normalization: The authors found that features extracted from foundation models are very sensitive to color variations. They also concluded that pure transformers are inherently more effective at capturing textures and colors than complex morphological features. This finding could be of interest, but it needs a more carefully evaluation, because the author did not use a color normalization technique, such as Macenko method, before feeding the image tiles into the foundation models.

4. Baseline alternatives: The study's central contribution is extended pre-training of UNI and demonstrated the superior of this model with the original UNI. Given the complexity and high computational demand of this method, it would be appreciated if the authors could also build a simple supervised approach to directly predict tissue types or archetypes from the features of original foundation models (e.g., UNI2-h as the strongest baseline), and ideally test on external cohorts (or via a cross-validation at least), and compare the performance of these models.

Minor comments:

1. Metho clarity: The extraction pipeline to obtain handcrafted features was outlined, but readers would benefit from more implementation detail and pointers to exact scripts.

2. Reproducibility: The exact values of k used in k-NN approaches for every case should be reported in the methods section. It's also similar for other hyperparameters such as min_dist, etc. Additionally, the code for data pre-processing should be provided.

3. Patient-count consistencies: Early text mentions “6 patients” (line 139), whereas Methods start 7 patients (8 patients total with patient A excluded, resulting 7 remaining).

Reviewer #5

(Remarks to the Author)
The work aims to boost the ability of histopathological foundation models to discriminate visually similar but molecularly distinct tumor regions that are a priori indistinguishable by baseline models, focusing on breast cancer molecular intratumor heterogeneity. To achieve such an objective, the authors has implemented the following works:
(1) Evaluation of existing hFMs: Six mainstream histological Foundation Models (e.g. UNI, Prov-GigaPath, etc.) were systematically assessed (hFMs) by comparing their ability to discriminate between different tissue types (e.g., fat, gland, carcinoma) and encode biological information.
(2) Introduction of a semantic richness metric: Shannon entropy (SE) was introduced as a metric to quantify the uniformity of information distribution in the models' representation space, which positively correlated with a model's capability to distinguish tissue types.
(3) Extended pretraining strategy: Extended pretraining of the UNI model was performed on invasive carcinoma tissues through using different regularization methods (KoLeo vs. KDE) and varying numbers of prototypes, resulting in a series of tumor-specialist models (T-UNI).
(4) Identification of tumor prototypes: Using the T-UNI models, the author successfully identified two major tumor prototypes:
i. c0: Enriched in RNA splicing and aberrant energy metabolism pathways.
ii. c2: Enriched in TGF-β signaling and associated with epithelial-mesenchymal transition (EMT).
(5) Molecular validation: The work validated the molecular distinctions of these prototypes using spatial transcriptomics data, demonstrating their recurrence across multiple patients and confirming their biological consistency and clinical relevance.

Major comments:
1. The study is limited by its reliance on a small cohort of only 8 HER2-positive breast cancer patients from a single public dataset. Therefore, the findings should be considered preliminary and require validation in larger, independent cohorts before they can be regarded as generalizable conclusions.
2. The study's exclusive focus on HER2-positive breast cancer represents a significant limitation, as it remains unclear

whether the identified prototypes and pathways are applicable to other molecular subtypes (e.g., Luminal A/B). This narrow scope limits the generalizability of the findings across the broader breast cancer population.
3. While the focus on the UNI model is understandable due to computational constraints, the study would be strengthened by a discussion on the potential generalizability of the proposed pre-training strategy. It would be valuable to speculate on whether applying the same approach to models like CTransPath would yield consistent or divergent prototypes.
4. The study's central finding, the identification of two distinct tumor prototypes, warrants further validation in clinical practices. Specifically, the clinical significance of these prototypes remains unclear, as their association with critical outcomes such as patient survival and treatment response has not been investigated.

Overall, this work identifies intriguing prototypes but remains at a preliminary stage. The key limitations regarding cohort size and lack of clinical outcome association need to be addressed. Significant strengthening through validation on larger, multi-centric cohorts and demonstration of clinical relevance is necessary to support the conclusions. I regret to conclude that the paper is not yet suitable for publication in its current form.

Version 1:

Reviewer comments:

Reviewer #1

(Remarks to the Author)
I'd like to thank the authors for their very thorough response to my comments. I have no additional questions or concerns.

(Remarks on code availability)
I have no detailed comments or concerns regarding the available code. The authors have provided a git repository which I believe is sufficient to address reproducibility questions.

Reviewer #3

(Remarks to the Author)
The authors have carefully and comprehensively addressed all of my previous concerns. The revised manuscript is substantially improved in terms of methodological clarity, validation, and biological and clinical relevance. I have some minor comments.

1. Several technical terms are introduced without clear definitions at first mention. For a multidisciplinary readership, brief explanations would be helpful.

2. The authors have provided a much clearer description of the ExPLoRa extended pre-training strategy, including the role of LoRA adapters, optimization objectives, and hyperparameter choices. Some sensitivity analysis are recommended.

3. The addition of survival analyses, including Cox regression and Kaplan–Meier curves, substantially enhances the translational relevance of the work. The number of event samples in the survival analysis is highly imbalanced, which may lead to statistical bias.

(Remarks on code availability)

Reviewer #4

(Remarks to the Author)
My previous comments have been addressed comprehensively. I have no further concerns.

(Remarks on code availability)
The code contains detailed instructions.

Reviewer #5

(Remarks to the Author)
I think the authors have adequately addressed all of my concerns.

(Remarks on code availability)

Version 2:

Reviewer comments:

Reviewer #1

(Remarks to the Author)
Thank you for addressing the outstanding items. I have no further clarifying questions or concerns.

(Remarks on code availability)

Reviewer #5

(Remarks to the Author)
All reviewers' comments have been addressed by the authors. I approve the current version of the manuscript.

(Remarks on code availability)
The code seems complete. I have not ran the code. The readme shows every step necessary to reproduce the paper.

Open Access This Peer Review File is licensed under a Creative Commons Attribution 4.0 International License, which permits use, sharing, adaptation, distribution and reproduction in any medium or format, as long as you give appropriate credit to the original author(s) and the source, provide a link to the Creative Commons license, and indicate if changes were made.
In cases where reviewers are anonymous, credit should be given to 'Anonymous Referee' and the source.
The images or other third party material in this Peer Review File are included in the article's Creative Commons license, unless indicated otherwise in a credit line to the material. If material is not included in the article's Creative Commons license and your intended use is not permitted by statutory regulation or exceeds the permitted use, you will need to obtain permission directly from the copyright holder.
To view a copy of this license, visit https://creativecommons.org/licenses/by/4.0/

RESPONSE TO REVIEWERS

We are grateful to the referees for the constructive feedback and helpful comments that have helped improve our study considerably and led to key new findings, thereby significantly increasing the impact of our work. We acknowledge that some aspects of the previous manuscript could have been clearer and that additional analyses and experiments were required. Overlapping concerns of the referees boil down to two major points:

A. Validation on independent and expanded cohorts

The referees requested validation of the proposed methodology on an independent dataset. We incorporated a publicly available cohort of 92 triple-negative breast cancer (TNBC) patients with matched H&E and spatial transcriptomics data. These analyses confirm that our extended pre-training framework is robust, reproducible, and generalizable. Extended pre-training consistently improves the ability of histopathology foundation models to mitigate patient-specific effects and to resolve molecularly defined tumor archetypes that recur across patients. These results validate the generalizability of the proposed approach and confirm that the benefits of extended pre-training are not dataset-specific. Using this cohort, we further demonstrate that RNA splicing-associated archetypes identified in HER2 breast cancer are also characteristic of TNBC. In addition, we generated new spatial transcriptomics data and show that RNA splicing-associated archetypes are also present in high-grade ovarian carcinoma, a cancer type well known for its pronounced heterogeneity. Although preliminary, these analyses support the potential extension of our findings beyond breast cancer. Altogether, these expanded datasets and increased cohort sizes demonstrate both the robustness of the proposed computational framework and the generalizability of RNA splicing signatures as defining features of tumor sub-compartments across multiple heterogeneous epithelial cancers.

B. The demonstration of the clinical relevance of our work

The referees also emphasized the need to establish the clinical relevance of the identified tumor archetypes. Leveraging survival information available in the TNBC cohort, we demonstrate that the identified tumor archetypes are significantly associated with overall survival. While immune-enriched archetypes are linked to favorable outcomes, RNA splicing-associated archetypes consistently predict poorer prognosis. While recent studies have highlighted the importance of RNA splicing in cancer, our work reveals that these signatures are spatially confined to specific tumor domains and, importantly, can be detected directly from standard H&E histopathology. This enables the construction of strong prognostic models using routinely acquired clinical data. These results establish the clinical relevance of the discovered archetypes and highlight their potential utility for patient stratification. In the future, such computationally efficient approaches may facilitate patient stratification and support the development of RNA-based therapeutic strategies by enabling in-depth molecular characterization of clinically relevant tumor archetypes.

In summary, we have conducted extensive additional experiments and analyses to address the reviewers' most critical concerns. These revisions substantially extend the scope and impact of the original manuscript, improve the clarity and coherence of the presentation, and place stronger emphasis on the biological and clinical significance of our findings. We hope that these changes satisfactorily address all reviewer comments. Below, we respond in detail to each referee's point.

Reviewer #1

The aim of the work is to characterize intratumor heterogeneity in breast cancer. To achieve this, the authors propose specializing foundation models by extending pre-training. They then use molecular data (spatial transcriptomics) to evaluate the clustering learned via imaging-only. The primary components of the analysis are: (1) systematic evaluations of biological concepts in foundation models (2) evaluation of extended pre-training approaches and (3) characterization of the molecular signatures.

Overall, I think an investigation of how well hFMs capture biology is of interest to the community. I thought the description of investigations into extended pre-training and the biological analysis were nicely done. However I have some questions before recommending this work for publication.

1. The dataset is described as using 100 x 100 pixel patches from the whole slide image, however most hFMs don't allow for inputs of this size. Could the authors describe the pre-processing performed? Given that the center-to-center distance is 200 microns, did the authors consider generating image patches at a resolution closer to the training data distribution (i.e. 256 x 256 px)?

Author's response

We thank the reviewer for pointing out this omission, which has been added to the Methods section. Our choice of **100 × 100 pixel patches** was motivated by the **diameter of the ST capture spots**, which is 100 µm at a resolution of 1 µm/pixel. This ensures that each image patch corresponds spatially to a single spot, thereby providing a direct and consistent alignment between the histological context and the associated molecular measurement. In other words, the patch size was chosen not primarily to match the typical input resolution of histopathology foundation models (hFMs), but rather to ensure a biologically meaningful correspondence between image and transcriptomic data.

Regarding preprocessing: although the raw image patches were extracted at 100 × 100 px, these were subsequently **resized to 224 × 224 px** (using bilinear interpolation) before being inputted to the models. This size matches the default used for pretraining hFM, even though its transformer backbones can operate on any patch size that can be tokenized into 14×14 px images. Recent models such as UNI already demonstrated the robustness of their representations w.r.t magnification, hence our analysis is expected to be robust to this resizing hyperparameter. Finally, note that before feeding the patches into the network, pixel intensities were normalized using the ImageNet normalizer as required by these models.

Author's action

We added the details in Methods (Section "**Full patches datasets**", subsection *"Patches preprocessing")* mentioned above for the HER2 dataset, as well as those for the TNBC and HGSOC datasets, given that the datasets have different H&E image magnification and spot sizes.

2. My understanding is that Shannon entropy is calculated for all samples and the ARI is calculated for each patient. Is this correct? Could the authors explain this choice if so?

Authors' response

We thank the reviewer for raising this point. Our decision to compute the ARI score on a per-patient basis followed established conventions in the literature (e.g., Long et al., 2023[1]). However, we agree that using

different levels of aggregation for Shannon entropy and ARI may introduce unnecessary asymmetry and potential confusion, particularly in the correlation analyses.

Authors' action

To address this issue, we have revised the figures so that Shannon entropy is now computed per sample and then averaged across patients, rather than being calculated globally across all samples (**Fig. 2c, Fig. 3f, Supplementary Fig. 3g**). This ensures that both metrics are computed at the per-sample level, and their mean values across patients are reported consistently. We note that it didn't affect the models ranking nor the observed correlation between Shannon entropy and ARI scores.

3. I'd like to understand better the choice to only do extended training on invasive cancer, as well as the choice to use the same dataset for extended pertaining and analysis. In the discussion, the authors note that they provide a data-driven alternative to manual annotation, however, it seemed to me that the approach required the invasive cancer annotation to be successful. Could the model have been specialized without these annotations? And could a different cohort of breast cancer data have been used to achieve the specialization?

Author's response

We thank the reviewer for these constructive comments. Below, we clarify the motivation and methodological considerations driving these choices.

(1) Motivation for extending training only on invasive cancer tissue

Our primary goal was to specialize the model for capturing intra-tumor heterogeneity in invasive breast cancer. Existing hFMs are trained on highly diverse datasets spanning multiple tissue types and disease states, which provides strong general recognition but can reduce sensitivity to subtle tumor-specific morphological patterns. Indeed, unsupervised clustering of tumor patches using embeddings from UNI, UNI2-h, and Prov-GigaPath showed poor cross-patient distribution, with many individuals dominated by a single cluster (**Fig. 3a; Supplementary Figs. 3a–d)**. By performing extended pretraining exclusively on invasive cancer patches, we encourage the model to focus on shared tumor phenotypes rather than patient-specific artifacts. This deliberate choice enhances the model's expertise in invasive cancer biology, rather than serving broad cancer-versus-non-cancer discrimination.

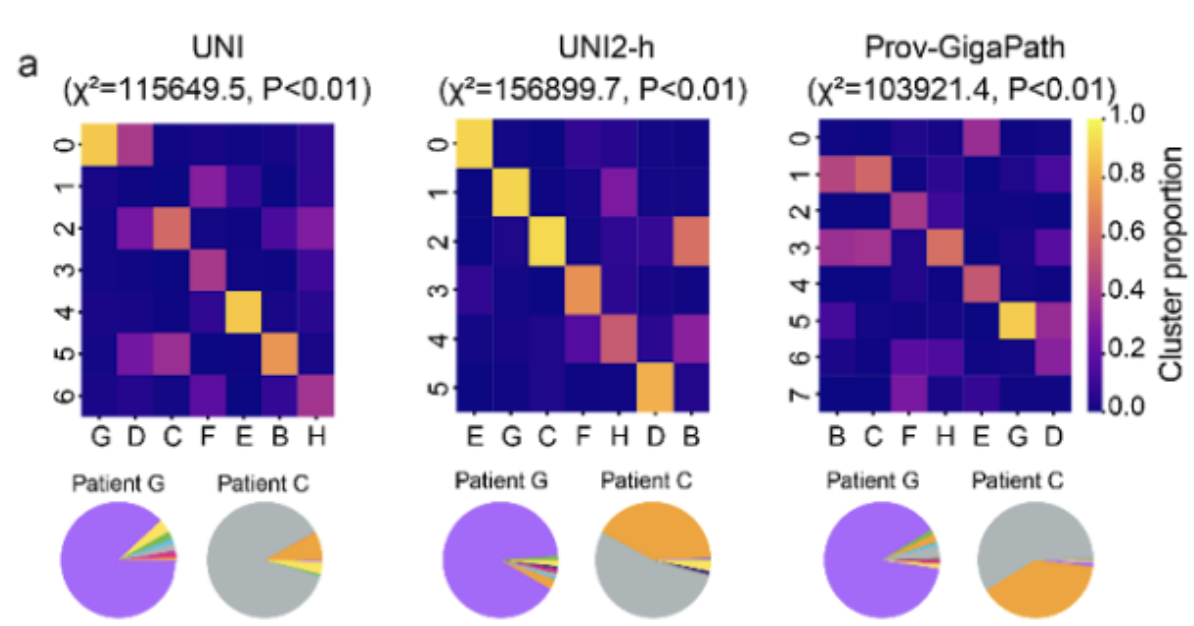

Figure 3a: Heatmap showing the distribution of patient's spots across predicted HER2 tumor archetypes, identified by clustering of invasive cancer patches using embeddings from UNI, UNI2-h and Prov-GigaPath models. Each column represents one patient, and row color intensity indicates the proportion of each patient's spots assigned to a given cluster. Hypergeometric tests were performed on the contingency tables and chi-square statistics and P-values are reported.

(2) Role of manual annotation and feasibility without it

While our pipeline requires invasive cancer patches as input for the extended pretraining phase, the amount of manual annotation needed to achieve this is intentionally kept minimal. In the HER2 dataset, only a small number of whole-slide images were manually annotated by experts, and in the TNBC dataset many patches remained unannotated after pixel-wise aggregation due to high intra-patch diversity. To address this, we substantially expanded the annotated dataset using a k-NN propagation strategy within the pretrained feature space, reducing reliance on manual effort. Moreover, the approach could be compatible with fully automated tumor-region identification tools. Future implementations could leverage automated segmentation or cell-based annotation methods, such as CellViT[2] to identify tumor patches, to entirely remove the need for manual annotations.

(3) Using different cohorts for training and evaluation

We appreciate the reviewer raising the important question regarding generalizability. To address it, we performed additional experiments following the same methodology used in the main analysis. Specifically, we conducted extended pre-training of UNI on an independent breast cancer cohort comprising 92 TNBC patients and then evaluated the resulting model on the HER2 dataset. As shown in **Figure R1** below, the model trained on the TNBC cohort (T-UNI-TNBC) continued to outperform the original UNI representations, exhibiting higher Shannon entropy and improved clustering characterized by clearer separation in the embedding space and reduced batch effects. However, its performance did not exceed that of the model directly trained on the HER2 dataset (T-UNI-HER2). Similarly, for molecular characterization, the T-UNI-TNBC model performed between UNI and T-UNI-HER2. This suggests that while the approach does generalize, its strongest benefit comes from **specializing representations to the specific dataset under study**. Our intention is not to propose a universal breast cancer model, but rather a **computationally lightweight and scalable pipeline** that can be rapidly adapted to new studies and cohorts, even in data-constrained settings.

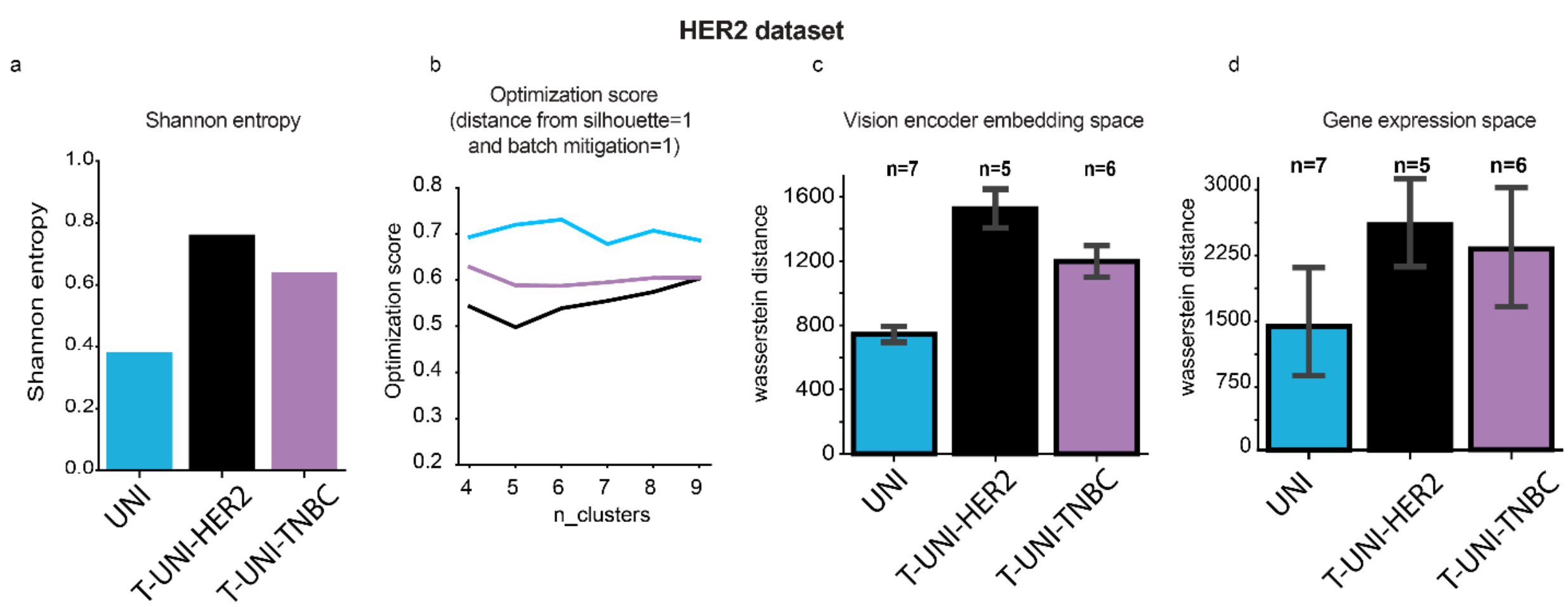

Figure R1: Model generalization experiment. a) Shannon entropy of the variance explained by principal components computed from HER2 embeddings using UNI, T-UNI-HER2, and T-UNI-TNBC representations. b) Optimization scores for UNI, T-UNI-HER2, and T-UNI-TNBC for cluster numbers 4–9. For each k, UMAP-k-means clustering is performed using the UMAP parameter set (minimal distance and number of neighbors), yielding the highest silhouette score. The optimization score is defined as the Euclidean distance between the observed (silhouette score, batch-effect mitigation score) and the ideal point (1,1), corresponding to perfectly compact clusters and complete mitigation of patient-specific batch effects. For each model, the optimal number of clusters is the one

that minimizes the optimization score. c) Pairwise distances in the image embedding space between clusters identified using UNI, T-UNI-HER2, and T-UNI-TNBC embedding spaces. d) Median per-patient pairwise distances in the gene expression space between clusters identified using UNI, T-UNI-HER2, and T-UNI-TNBC embeddings.

Author's action

(1) In response to the reviewer's comment, we have better motivated the choice to perform extended pretraining exclusively on invasive cancer patches in the main text. Accordingly, the section "**Extended pre-training enhances tumor-specific representations while reducing patient-specific bias"** now begins with the results of unsupervised cancer clustering of tumor patches using embeddings derived from UNI, UNI2-h and Prov-GigaPath.

(2) We also clarified the description of k-NN reannotation in the Methods section. The beginning of the paragraph has been modified as follows: **"***To further investigate intratumor heterogeneity, we first extracted invasive cancer patches from the full datasets by selecting all patches falling within the corresponding annotated invasive cancer regions. Because only a small number of slides were annotated in the HER2 dataset, and many patches remained unlabeled in the TNBC dataset, we expanded the subset of invasive cancer patches using a k-nearest neighbors (k-NN) classifier trained on the labeled spot patches. We applied k-NN to all unlabeled patches in the full dataset and retained those classified as invasive cancer for downstream analysis.***"**

(3) Finally, when introducing extended pretraining in the section "**Extended pre-training enhances tumor-specific representations while reducing patient-specific bias"**, we are adding a word on this experiment that justifies training and testing on the same dataset", we added a justification for training and testing on the same datasets: "*Notably, we performed extended pre-training separately for the HER2 and TNBC datasets, as their associated tumour types exhibit distinct biological and morphological characteristics. Therefore, we experimentally found it most appropriate to specialize models separately for each subtype, to minimize technical confounding and ensure that any learned features are truly cancer-specific.*"

4. Did the authors consider defining archetypes at a level greater than the tile? The strong patient effects seems somewhat surprising given generalization results in the literature. Could it be that at small resolution patient effects dominate over TME characterization?

Author's response

We did not consider defining archetypes at a level larger than the tile, as tiles were selected to coincide with spatial transcriptomics spots, allowing us to map morphological features directly to gene expression. In addition, the available spots are not necessarily perfectly adjacent, which prevents straightforward aggregation into larger units. Nonetheless, we agree that developing methods to determine optimal archetype sizes is a promising direction for future work.

The strong patient effects we observe are consistent with recent studies[3], which show that batch-related variability affects multiple tissue representation approaches beyond those evaluated in our work. Notably, this study also reports that classifiers trained on hFM embeddings generalize poorly to data collected at other clinical centers, highlighting potential limitations in hFM's evaluation protocols for assessing representation quality. These findings further underscore the relevance of our methodology, including our alternative evaluation protocols and proposed extended pretraining strategies, which mitigate batch effects and enhance molecular characterization.

Regarding the last point, although the intuition is appealing, we do not expect current DINOv2-based models to capture such patterns reliably. Because these models rely on enforcing consistency between

teacher and student embeddings, they may inadvertently exploit batch effects as an easy source of consistency, rather than learning the intended biological variability.

5. Did the authors consider applying stain normalizing techniques to pre-process the data and observe whether or not patient differences still dominate the clustering?

Author's response

We thank the reviewer for this valuable suggestion regarding stain normalization. We evaluated the impact of applying Macenko normalization [4] prior to extracting UNI embeddings. As shown in **Figure R2**, stain normalization resulted in a slight reduction of batch effects; however, this came at the cost of reduced biological signal. Specifically, ARI scores for tissue-type discrimination decreased for 5 out of 7 patients, and Shannon entropy scores were lower. Given this trade-off, we decided not to include stain normalization in the final pipeline. Overall, batch effect mitigation with Macenko normalization did not reach the performance achieved by our models retrained in the "full" setup, and some biological information was lost.

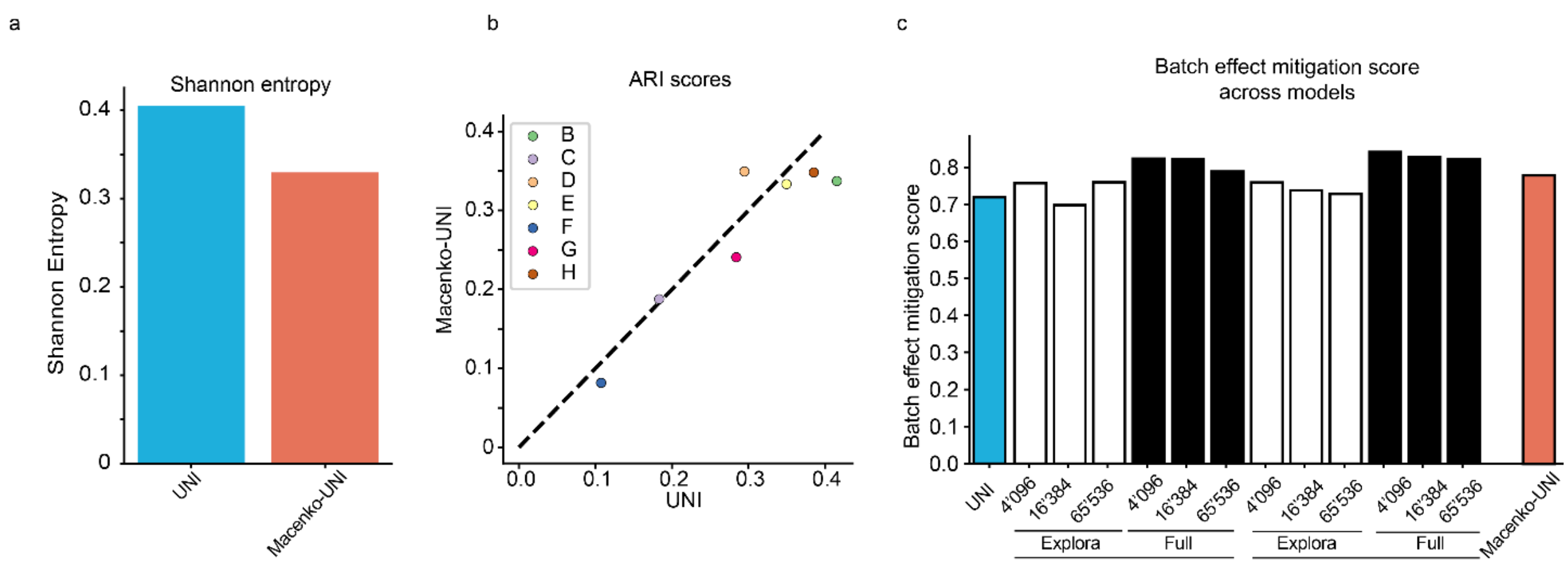

Figure R2: Stain-normalization effects on batch effect and captured biological signal on the HER2 dataset. a) Shannon entropy of the variance explained by principal components computed from HER2 embeddings. Embeddings were generated from the same patches under two setups: (1) UNI - patches input directly to the UNI model, and (2) Macenko-UNI - patches first normalized using Macenko stain normalization before input to UNI.
b) Adjusted Rand Index (ARI) scores for tissue-type discrimination across 7 patients, comparing embeddings from the two setups (UNI vs. Macenko-UNI).
c) Batch effect mitigation score (1 − ARI computed using patient labels) for UNI, all T-UNI models presented in the preprint, and Macenko-UNI embeddings.

Author's action

We added a sentence to mention that we tested the Macenko stain normalization technique in the Method section: *"We also evaluated the impact of applying stain normalization prior to feature extraction, using the Macenko[4] method. While this procedure modestly reduced batch effects, it also led to a loss of biological signal, reflected by decreases in both the Shannon entropy of explained variance and the ARI scores. Consequently, stain normalization was omitted from the final analysis."*

The core open question I have is whether or not the authors are arguing that hFMs must be specialized to particular cohorts, i.e. extend pre-training using the cohort of interest, or if extended pre-training is more general, e.g. specializing for breast cancer.

Author's response

We thank the reviewer for raising this key point. We are indeed arguing that current hFMs must be specialized to particular cohorts, i.e. extend pre-training using the cohort of interest when interested in resolving subtles tissue types. Our generalization experiments have shown that the approach does generalize, but its strongest benefit indeed comes from specializing representations to the specific dataset under study. Please refer to your comment #3 for more details.

Small comments:
1. I found the authors overused the word "comprehensive". Please considering removing or revising.

Authors' response

We thank the reviewer for pointing out this repetition.

Authors' action

We revised the manuscript to reduce redundancy by removing or replacing repeated instances of the word *"comprehensive"*, retaining only one occurrence throughout the text.

2. I found referring to the Ciga et al. model as "SimCLR" confusing as I thought at first it was a natural image model. Perhaps the authors could call it something else?

Authors' response

We thank the reviewer for pointing out this confusion.

Authors' action

We have renamed the model to "Histo-SimCLR" to clarify that we are referring to the Ciga et al. [5] model trained on histopathology images using the SimCLR framework. It has been updated in the text and figures accordingly.

3. I don't think it's fair to say that hFMs have architectural diversity when almost all use transformers. Please consider revising (line 126)

Authors' response

We thank the reviewer for this comment and agree that our sentence wasn't clear enough.

Author's action

This sentence has been removed from the main text. We now highlight the differences between the hFMs as follows: *"In particular, hFMs produce high-dimensional representations, yet it remains unclear which*

semantic signals they encode or how choices in pretraining, regularization, and model or data scale influence this information."

4. Could Figure3f be edited to have a consistent y-axis? I think that would help demonstrate the result.

Authors' response

We thank the reviewer for this suggestion and agree the different scales were not helping demonstrating the results.

Authors' action

We have modified the y-axis to ensure consistency and improve readability. The updated figure is now **Figure 3h** for the HER2 dataset, and the corresponding figure for the TNBC dataset is shown in **Supplementary Figure 3i**.

5. The authors commented that they could consider multi-modal models with IHC in future work, but Virchow2 is already multi-modal with respect to IHC.

Authors' response

We thank the reviewer for this comment. What we meant is that we could consider multi-modal models and study extended pre-training strategies while leveraging multi-modal inputs too, instead of only operating on H&E images.

Authors' action

We clarified the text as follows *"Increasing cohort size and applying extended pre-training to multimodal pathology models, including immunohistochemistry or multiplexed imaging, may uncover richer archetypes."*

Reviewer #2, Withdrawn

Reviewer #3

The paper presents a compelling and thorough investigation into the potential of histopathology foundation models (hFMs) for identifying molecularly distinct tumor regions within invasive breast cancer using routine H&E-stained slides. The study successfully extends the use of these models through advanced pretraining techniques and highlights their ability to uncover tumor heterogeneity that would otherwise be undetected by conventional methods. The integration of molecular data from spatial transcriptomics with AI-driven histopathology analysis is a significant contribution to the field and could pave the way for more effective, precision-based cancer diagnostics. Here are some concerns.

1. Details of the ExPLoRa Method: The description of the training strategy and hyperparameter settings is not sufficiently clear. It is recommended to include a table of hyperparameters in the Supplementary section and briefly explain the optimization strategy. In the Methods section, please briefly discuss the relationship between model capacity and data complexity, and provide the rationale for the selection of hyperparameters.

Authors' response

We thank the reviewer for these remarks. In a vision transformer with L layers, ExPLoRa leverages the popular LoRA adapters[6], permitting "small" updates of the first L-1 layers, and a full update of the L-th layer, to perform extended pre-training. LoRA, short for "low-rank adapters", involves adding a small number of new parameters to the model and training them while keeping the original model parameters frozen. LoRA defines these new parameters as a low-rank representation of projection layers. In the ExPLoRA framework, these adapters are only created for the query and value matrices in the self-attention mechanism. LoRA depends on a rank hyperparameter with a default value of 64, used in ExPLoRA and kept fixed in our study.

Regarding the other hyperparameters relating to the DinoV2 model, for the Koleo regularization we fixed its coefficient in the loss to 0.1 being the selected default in the literature (original DinoV2 paper[7], UNI[8] etc), and for KDE regularization we fixed its coefficient in the loss to 0.05 and its bandwidth parameter to 5 as validated in Virchow2[9]. The other hyperparameters, such as batch sizes and training epochs, are detailed in the paragraph "Hyperparameters selection". Any parameters not specified follow the DinoV2 defaults. Instead of a supplementary table, we provide configuration files for all 12 T-UNI models for each dataset (HER2 and TNBC) here: Github link.

Author's action

We added the details mentioned above on the Explora method as well as hyperparameters in the Methods Section, completing the paragraph "**Extended pre-training of the UNI foundation model**".

2. The results are based solely on HER2+ breast cancer data, with limited external validation. It is recommended to supplement the study with external datasets to validate the model's adaptability across different subtypes. Alternatively, please indicate plans to include such external data for future validation.

Authors' response:

We thank the reviewer for this comment and appreciate the suggestion to validate the method on external datasets. We note that the reviewer's request for external validation could be understood in three possible ways, as it could refer to:

1. Validation of the method for identifying tumor archetypes
2. Validation of specific results (e.g., the RNA splicing and TGF-β archetypes found in HER2)
3. Transferability of models trained on HER2 to other breast cancer subtypes.

Author's action:

- To address points 1 and 2, we have supplemented the study with an additional dataset of 92 TNBC patients[10]. The manuscript now integrates this dataset alongside the HER2 dataset, showing that our method for uncovering spatially and molecularly defined tumor archetypes via extended pretraining is robust and reproducible. Importantly, we also confirm the presence of a recurrent RNA splicing archetype with prognostic relevance in TNBC, validating the biological findings from the HER2 dataset.

- Regarding point 3, as discussed in comment #3 from Reviewer #1, our approach is transductive. We perform extended pretraining separately on each dataset, as different breast cancer subtypes have distinct biological and morphological characteristics. Our experiments demonstrate that this approach yields optimal performance and tumor-specific representations for each subtype, rather than building a single generalist model.

In summary, the method is validated on an independent dataset, the key biological findings are reproducible, and the model's design emphasizes specialization per cancer subtype.

3. The comparison of prediction differences between UNI and T-UNI in specific regions is not sufficiently intuitive. It is recommended to include a comparative figure that clearly demonstrates the differences in the recognition capabilities of the two models in typical tissue regions.

Author's response

We thank the reviewer for this comment. As the notion of "recognition capabilities in typical tissue regions" can be understood in different ways, we addressed this point from two complementary perspectives: (i) the capability to discriminate between major tissue types (invasive cancer, adipose tissue, immune infiltrate, etc.) and (ii) the capability of the models to identify tumor archetypes within invasive cancer regions.

Author's action:

1. **Tissue-type discrimination**
   Differences in tissue-type recognition between UNI and T-UNI models are quantitatively shown in **Fig. 3f** and **Supplementary Fig. 3g**, which reports ARI scores across models. In addition, **Fig. 3g and Supplementary Fig. 3h** visualize the change of T-UNI models in ARI relative to UNI, making clear that ExPLoRa-based models improve tissue-type discrimination, whereas full retraining leads to reduced performance compared to UNI. These plots provide an intuitive comparison of how the T-UNI models differ in their ability to recognize canonical tissue regions compared to UNI.

2. **Tumor archetype identification within invasive cancer**
   To illustrate differences in recognizing tumor heterogeneity, we report quantized Wasserstein distances between clusters in the vision encoder embedding spaces (**Fig. 4a and Supplementary Fig. 4a**), which shows that our T-UNI model yields more clearly separated tumor

archetypes than UNI. For direct spatial visualization, **Fig. 4g** presents HER2 slides with invasive cancer regions colored by T-UNI archetypes, while the corresponding UNI-based clustering is shown in Supplementary **Fig. 3a**. Together, these figures highlight differences in how the two models resolve intra-tumor structure.

Overall, we believe these visual and quantitative analyses address the request by clearly demonstrating how UNI and T-UNI differ in their recognition of both tissue regions and tumor-specific archetypes.

4. The value of tumor subtypes has not been evaluated in relation to prognosis or treatment response. If the data is available, it is recommended to include Kaplan-Meier curves or other survival analyses. If not, please consider discussing potential research directions in the discussion section.

Authors' response

We thank the reviewer for this valuable suggestion. We agree that evaluating the clinical relevance of the identified tumor archetypes is important to strengthen our analysis.

Author's action

To address this comment, we used the TNBC dataset[10], with available survival data, and applied our pipeline (extended pre-training on invasive cancer patches followed by archetype discovery) to derive patient-level archetype proportions, which were used as features in a Cox regression model (**Fig. 6, Supplementary Fig. 6**, section **"RNA splicing as a prognostic biomarker in breast cancer with potential to generalize across epithelial tumors"**). In brief, our T-UNI model achieved a concordance index of 0.74, outperforming UNI (0.68). Cox hazard ratios indicated that the RNA-splicing-associated archetype predicts worse prognosis, while the immune-associated archetype is protective. Patients were stratified into high- and low-risk groups based on their Cox risk scores and Kaplan–Meier analysis revealed a significant survival difference between the groups ($p = 1.56 \times 10^{-5}$).

5. The paper has not explored whether the method can be extended to other diseases or tissues. It is recommended to add a discussion in the Discussion section regarding the model's generalizability and potential applications across different diseases.

Author's response

We thank the reviewer for this comment. To explore generalizability, we extended our analysis to another breast cancer subtype (TNBC), demonstrating that the method can be applied across breast cancer subtypes. Preliminary analysis of a high-grade serous ovarian carcinoma (HGSOC) sample further suggests potential applicability to other epithelial tumors (**Fig. 6f**). These findings highlight the broader utility of our approach for studying diverse diseases and tissues.

Authors' action

We have added the TNBC analysis and preliminary ovarian cancer results to the main text and figures, therefore illustrating the generalizability potential of our method.

6. Although dysregulation of RNA splicing and TGF-β pathways has been identified, the paper does not delve into how these pathways drive tumor heterogeneity or affect treatment response. It is recommended

to further elaborate on these pathways in the context of known breast cancer mechanisms. While several relevant studies are cited, the Results section does not explicitly compare the tumor archetypes identified in this study with existing breast cancer subtypes. It is suggested to reference current molecular subtypes when discussing patient-specific archetypes to strengthen the contextual support for the results.

Author's response

We thank the reviewer for this insightful comment and agree that a deeper biological contextualization of the identified archetypes was warranted. While part of this concern is addressed through survival analyses and Cox regression modeling (see response to comment #4), we further strengthened the biological interpretation of the archetypes by explicitly linking them to known breast cancer mechanisms and previously described molecular programs.

Author's action

With the addition of the TNBC dataset, we revised the manuscript to place greater emphasis on the RNA splicing archetype rather than the TGF-β–associated archetype, as this signal was consistently observed across subtypes and showed strong prognostic relevance. To better contextualize our findings within existing literature, we also added the following sentences to the Results section:

"*In contrast, the archetype characterized by alternative-splicing activity (cluster 1) contributed the second-largest effect in the model and was associated with poorer prognosis. Prior studies have demonstrated that alternative-splicing signatures can stratify breast cancer patients into distinct risk groups, with high-risk splicing programmes predicting reduced survival* [11,12]"

"*Moreover, consistent with previous reports showing that immune-rich or TIL-high TNBC tumors exhibit improved survival and treatment response* [50–52]*, our immune-associated archetype (T3) was linked to better prognosis (**Fig. 6d**). In contrast, the archetype characterized by alternative-splicing activity (T1) contributed the second-largest effect in the model and was associated with poorer prognosis. Prior studies have demonstrated that alternative-splicing signatures can stratify breast cancer patients into distinct risk groups, with high-risk splicing programs predicting reduced survival* [53,54]*.*"

7. Page 8 mentions that full training leads to a decrease in ARI scores, possibly due to the loss of previously encoded biological concepts. It is recommended to more clearly explain the mechanism of this "catastrophic forgetting" and discuss how future training strategies can be optimized to balance new and old knowledge.

Author's response

We thank the reviewer for this comment. Catastrophic forgetting is the phenomenon where Neural Networks lose previously learned information when training on new data. It is widely studied[16], notably in the context of continuous learning for LLMs, but the exact cause of this forgetting remains elusive. Nevertheless, mitigation techniques such as regularisation or dynamic model architectures have been proposed and shown promise. We use ExPLoRA to mitigate the risk of catastrophic forgetting, as detailed in our answer to question 1. Our experiments show that ExPloRa succeeds in mitigating forgetting concepts critical for tissue type discrimination. Future training strategies could combine LoRA adapters with regularisation techniques such as Elastic Weight Consolidation[17].

Author's action
In the revised manuscript, we place less emphasis on the notion of catastrophic forgetting and no longer explicitly use this term in order to avoid potential confusion for the reader. Nevertheless, our experimental results indicate that forgetting is not an issue in the context of model specialization. Specifically, T-UNI *full* (in which all model weights are updated) exhibits an increased ability to learn tumor-specific concepts. Below, we report the sentences from the main text that support this interpretation:

- "*In contrast, the full training setup decreases ARI scores while increasing SE, suggesting that it causes the model to lose previously encoded biological concepts essential for accurate tissue classification (**Fig. 3e-g; Supplementary Fig. 3f-h**).*"
- "*Notably, this trend reversed when restricting the analysis to invasive cancer patches, indicating that model specialization enhances encoding of cancer-specific features while deprioritizing features primarily relevant to other tissue types.*"
- "*Crucially, we demonstrate that while full extended pre-training on invasive cancer patches reduces the model's ability to distinguish general tissue types (e.g., breast glands, immune cells) and broad handcrafted features, it significantly enhances the model's capacity to learn tumor-specific concepts and effectively mitigates patient-related batch effects.*"

8. Page 18 mentions that the UMAP-k-means clustering parameters are optimized for the best ARI score, but the optimization process (e.g., parameter range or algorithm) is not specified. It is recommended to provide a detailed hyperparameter selection process in the supplementary materials.

Author's response

We thank the reviewer for pointing this out. Note that in the case of UMAP-k-means for tissue type identification, only UMAP parameters are optimized, while k for k-means is fixed to 6, which corresponds to the number of tissue categories. We clarify the UMAP parameters optimization pipeline in the method section.

Author's action

We clarified the methods section as follows:

"***Clustering of different tissues** [...] Clustering was performed on the image representations obtained from various hFMs or from our selected handcrafted features taken as a whole. Performance was measured using Adjusted Rand Index (ARI) scores computed using the original tissue types as true labels ("ARI with tissue type"). Before applying k-means clustering (with k fixed to the known number of classes), we computed two-dimensional UMAP embeddings to capture non-linear relationships in the image representations. Following Van Assel et al.[18], we evaluated a range of UMAP hyperparameters, testing n_neighbors values of {10, 30, 50, 100, 150, 200, 250, 300, 350, 400} and min_dist values of {0.001, 0.1}. For each combination of hyperparameters, we selected the configuration that maximized the Adjusted Rand Index (ARI) with respect to tissue type. Clustering was performed either on the full dataset or on each patient individually, with the UMAP parameter optimization repeated for each analysis (i.e., for each patient and each model).*"

9. Pages 15-16 describe 153 handcrafted features (such as nuclear morphology, texture, color, etc.), but do not discuss whether these features comprehensively cover the biological information in H&E images. It is recommended to analyze the representativeness of these handcrafted features or perform a more in-depth comparison with deep learning features.

Author's response

Thank you for this insightful comment. First, note that the set of 153 handcrafted features was reduced to 131 by removing cell morphological Zernike moment descriptors. This choice was motivated by two factors: (i) results on the HER2 dataset were unchanged with or without these descriptors, and (ii) Zernike moment computation per cell is computationally intensive, making it impractical for the 94 TNBC slides. Therefore, removing these features ensured comparability between HER2 and TNBC datasets.

Regarding the representativeness of the handcrafted features: as detailed in the *Methods* section (paragraph "Handcrafted feature extraction"), our selection of handcrafted features is grounded in the scMTOP methodology, which has already demonstrated strong relevance for characterizing a wide range of tumor ecosystems. We further complemented these features with additional descriptors of morphology, texture and cell types inferred by CellVIT. While there is broad consensus in the literature that the underlying concepts represented by these features (e.g., morphology, texture) are sufficient to characterize tissue images, the challenge lies in embedding these concepts into a compact and discriminative representation. For example, one could extend our handcrafted feature set—which often relies on low-order statistics over continuous distributions (e.g., mean, standard deviation)—by incorporating additional statistics such as quantiles or percentiles. However, this expansion is effectively unbounded and quickly becomes inefficient compared to representations learned through deep models.

Author's action

Although this was already suggested by Shannon entropy and ARI scores on handcrafted features, we performed a new supervised analysis comparing tissue type classification using handcrafted features versus deep features from hFMs to further assess their biological relevance. As shown in **Supplementary Fig. 2f**, handcrafted features already achieve strong performance (macro F1 ≈ 0.5) across both datasets using logistic regression, 1-NN, and 5-NN classifiers. Considering that there are six tissue types to predict, this score is well above random. Importantly, the performance of handcrafted features is comparable from that of hFMs, further confirming the biological relevance of our chosen handcrafted feature set.

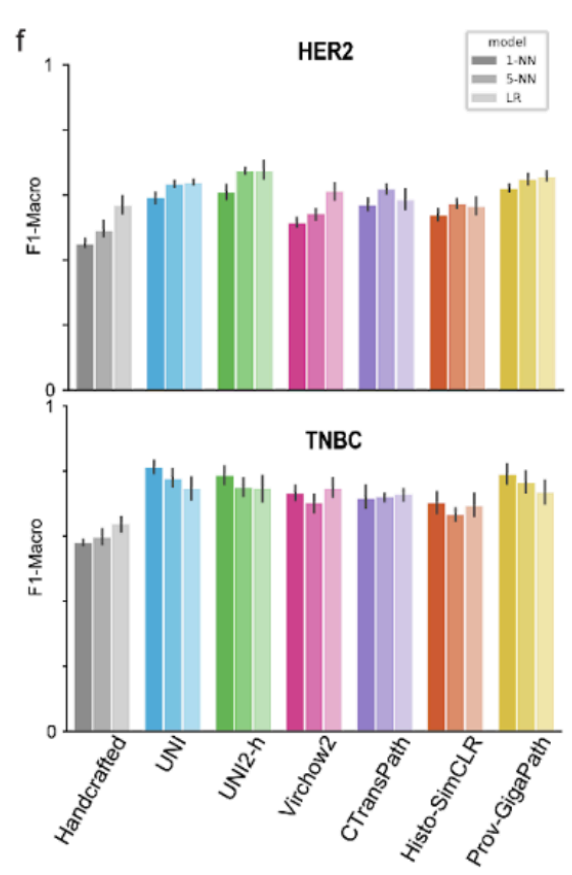

Figure. 2f: Macro F1 scores from 1-NN, 5-NN and logistic-regression classifiers trained on embeddings from all models of the HER2 and TNBC datasets. Tissue-type prediction was evaluated using 5-fold stratified cross-validation with scikit-learn default hyperparameters.

10. Although Combat correction is mentioned, its impact on the gene expression space is not clearly explained. It is recommended to show the UMAP projection differences of the gene expression space before and after correction in the Results section to validate the correction effect.

Author’s response

We thank the reviewer for this important comment. While initial UMAP projections suggested that Combat correction helped align patients (previously Supplementary Fig. 4c), we chose to now use the raw gene embedding space for our analyses for two main reasons:

1. **HER2 dataset:** Wasserstein distances between tumor archetype clusters were computed **within each patient**. Because batch effects occur **between patients**, they do not influence these within-patient comparisons. Applying batch correction could introduce unnecessary distortions, so raw embeddings are more appropriate.
2. **TNBC dataset:** Due to fewer invasive cancer patches per patient, Wasserstein distances were computed **across all patients**. However, with a relatively large patient cohort (n = 92) compared to the small number of archetypes (4–9), gene expression batch effects are expected to have a negligible impact on overall Wasserstein distances between archetypes.

Author’s action

The reported Wasserstein distances in the molecular embedding space have been updated accordingly to reflect distances in the uncorrected gene expression space and the text has been updated to remove Combat-correction description. Figure legends have been updated as well.

11. If possible, it is recommended to include more patients.

Author’s response

We thank the reviewer for this suggestion.

Author’s action

We included the TNBC dataset[10] composed of 92 patients to the manuscript.

Reviewer #4

In this manuscript, Fournier et al. systematically evaluated the biological features extracted by several histopathology foundation models and proposed an extended pre-training approach on invasive tumor patches to specialize the foundation model to enhance the model's ability to capture tumor-specific biological information. Using this approach, they reported two co-existing tumor archetypes linked to RNA splicing and TGF-β dysregulation, respectively.

While the study is timely and potential impactful, several limitations should be addressed:

Major comments:
1. External validation and cohort size: The core analyses are performed on the HER2-positive breast cancer dataset (36 WSIs, 8 patients; patient A excluded; downstream analyses use 7 patients), without an external validation cohort. This raises concerns about overfitting and generalizability.

Authors' response

We thank the reviewer for this question. We kindly refer the reviewer to comment #2 from reviewer #3.

2. Robustness: Quantitative measures such as Shannon entropy (SE) and Adjusted Rand Index (ARI) were reported at patient-level. Given there are only 7 patients, it's hard to judge the results, for example the correlation between SE and ARI across the 7 points. This could be partially improved by demonstrated this results at slide-level as they have approximately 30 slides available.

Authors' response

We thank the reviewer for this insightful comment. We would first like to clarify that the SE barplots are reporting the SE computed on the whole dataset, not per patient (**Fig. 2b**). Then, the points shown in the SE-ARI correlation plots represent the different foundation models we evaluated, not individual patients. However, we fully agree that the original plot could be misleading, since the SE was computed over the entire dataset while the ARI values reflected an average across patients.

Authors' action

To ensure consistency and interpretability, we revised our analysis as follows:

1. We changed de SE-ARI correlation plots (**Fig 2c, Fig 3f, Supplementary Fig. 3g**) and now report **mean Shannon entropy vs. mean ARI**, both computed patient-wise and reporting the mean. Clarifications have also been added to the legends, specifying explicitly whether the SE is computed overall or patient-wise.
2. To address concerns about statistical power, we now include results from an additional cohort of 92 TNBC patients, which further confirm the robustness of our findings for Shannon entropy and ARI scores.

3. Color variation and stain normalization: The authors found that features extracted from foundation models are very sensitive to color variations. They also concluded that pure transformers are inherently more effective at capturing textures and colors than complex morphological features. This finding could be of interest, but it needs a more carefully evaluation, because the author did not use a color

normalization technique, such as Macenko method, before feeding the image tiles into the foundation models.

Authors' response

We thank the reviewer for this insightful comment and kindly refer to our answer to question #5 of reviewer #1. In a nutshell, we observed that applying Macenko before inferring patch representations indeed reduced batch effects, but it also decreased SE and tissue classification scores (ARI).

Author's action

We added a sentence to mention that we tested the Macenko stain normalization technique in the Method section: *"We also evaluated the impact of applying stain normalization prior to feature extraction, using the Macenko[4] method. While this procedure modestly reduced batch effects, it also led to a loss of biological signal, reflected by decreases in both the Shannon entropy of explained variance and the ARI scores. Consequently, stain normalization was omitted from the final analysis."*

4. Baseline alternatives: The study's central contribution is extended pre-training of UNI and demonstrated the superior of this model with the original UNI. Given the complexity and high computational demand of this method, it would be appreciated if the authors could also build a simple supervised approach to directly predict tissue types or archetypes from the features of original foundation models (e.g., UNI2-h as the strongest baseline), and ideally test on external cohorts (or via a cross-validation at least), and compare the performance of these models.

Author's response

Thank you for these suggestions. To provide supervised evaluations, commonly used in the literature, and to compare them with our unsupervised approaches, we evaluated the embeddings derived from all original hFMs using simple classifiers, including logistic regression as well as 1-NN and 5-NN classifiers using 5-fold stratified cross-validation. The results, reported in **Supplementary Figure 2f**, are summarized using macro-F1 scores to account for class imbalance appropriately. Overall, all three classification methods exhibit similar performance across models and datasets. Macro-F1 scores are generally higher on the TNBC dataset, which is expected given its finer-grained region annotations and reduced patch mislabeling. Consistent with the trends observed for ARI scores, more recent transformer-based models, ProvGigaPath, UNI, and UNI2-h, demonstrate superior performance.

However, these supervised techniques do not allow the embeddings themselves to be updated to better reflect the class structure; therefore, they cannot be used to define archetypes for downstream molecular analyses. In addition, using hidden representations from a standard MLP classifier drastically simplifies the embedding space, removing much of the molecular variability that is crucial for identifying meaningful archetypes.

Consequently, only more sophisticated strategies, such as combining self-supervised extended pre-training with auxiliary classification objectives (e.g., tissue type or cancer vs. non-cancer), are likely to yield embeddings and archetypes that are both biologically informative and class-aware. Given the methodological complexity of such an investigation, we consider it an interesting direction for future work, out of the scope of our paper.

Author's action

We updated **Supplementary Fig. 2** with the supervised evaluations (panel **f**).

Minor comments:

1. Metho clarity: The extraction pipeline to obtain handcrafted features was outlined, but readers would benefit from more implementation detail and pointers to exact scripts.

Author's response:

We thank the reviewer for this suggestion.

Author's action:

To avoid overloading the Methods section, we did not include full implementation details directly in the manuscript, as the pipelines using CellViT, scMTOP, and scikit-image are already described in the text. In addition, **Supplementary Table 2** provides a detailed description of each handcrafted feature. We have now added the following sentence to the Methods section to guide readers to the exact implementation:
"Implementation details are available on GitHub (https://github.com/AI-for-RNA-Biology/TumorArchetype_FM, section Handcrafted features extraction), including instructions and scripts for running the handcrafted feature extraction pipeline."
The repository README provides clear step-by-step instructions on which scripts to use and how to execute the handcrafted feature extraction workflow.

2. Reproducibility: The exact values of k used in k-NN approaches for every case should be reported in the methods section. It's also similar for other hyperparameters such as min_dist, etc. Additionally, the code for data pre-processing should be provided.

Author's response

We thank the reviewer for pointing out these ambiguities, which have been addressed by revising the method section. All code required for this study is available in the TumorArchetype-FM repository. The generation of patch datasets from raw WSIs is implemented in the script compute_patches.py (TumorArchetype-FM/scripts/compute_patches.py) and is detailed at the beginning of the README.

Authors' action

For the k-NN parameters: clarifications have been added in the two related sections: *"**Invasive cancer patches datasets** [...] for the HER2 dataset (median F1 score = 0.82; median accuracy = 0.84; k = 7) [...] For the TNBC dataset, we used predictions derived from UNI representations (median F1 score = 0.98; median accuracy = 0.98; k = 3) [...]"* and *"**Identification of invasive cancer archetypes [...]** Hence the deep features from these refined models are used to get a better curated subset of invasive cancer patches following the same k-NN strategy as above, selecting the optimal value of k by maximizing the median F1 score across patients, based on a grid search with k ranging from 3 to 21 **(Table S6)**."*

We also updated **Supplementary Figure 7b** and added the exact value of k leading to the best F1-score for all baseline hFMs and both datasets. Similarly, we added the optimal k values for all retrained models in the new supplementary table **Table S6**.

For the UMAP-kmeans parameters: we clarified the text as follows in the two related sections:
***"Clustering of different tissues** [...] Before applying k-means clustering (with k fixed to the known number of classes), we computed two-dimensional UMAP embeddings to capture non-linear relationships in the image representations. Following Van Assel et al.[18], we evaluated a range of UMAP hyperparameters, testing n_neighbors values of {10, 30, 50, 100, 150, 200, 250, 300, 350, 400} and min_dist values of {0.001, 0.1}."* and ***"Identification of invasive cancer archetypes [...]** evaluating the same range of hyperparameters as in previous experiments (n_neighbors ∈ {10, 30, 50, 100, 150, 200, 250, 300, 350, 400} and min_dist ∈ {0.001, 0.1})."*

Moreover, we added in **Table S6** the UMAP-kmeans parameters selected for the identification of invasive cancer archetypes.

3. Patient-count consistencies: Early text mentions "6 patients" (line 139), whereas Methods start 7 patients (8 patients total with patient A excluded, resulting 7 remaining).

Author's comment

We thank the reviewer for noticing this typo.

Authors' action

The typo has been corrected by replacing the mention "6 patients" by "7 patients".

Reviewer #5

The work aims to boost the ability of histopathological foundation models to discriminate visually similar but molecularly distinct tumor regions that are a priori indistinguishable by baseline models, focusing on breast cancer molecular intratumor heterogeneity. To achieve such an objective, the authors has implemented the following works:
(1) Evaluation of existing hFMs: Six mainstream histological Foundation Models (e.g. UNI, Prov-GigaPath, etc.) were systematically assessed (hFMs) by comparing their ability to discriminate between different tissue types (e.g., fat, gland, carcinoma) and encode biological information.
(2) Introduction of a semantic richness metric: Shannon entropy (SE) was introduced as a metric to quantify the uniformity of information distribution in the models' representation space, which positively correlated with a model's capability to distinguish tissue types.
(3) Extended pretraining strategy: Extended pretraining of the UNI model was performed on invasive carcinoma tissues through using different regularization methods (KoLeo vs. KDE) and varying numbers of prototypes, resulting in a series of tumor-specialist models (T-UNI).
(4) Identification of tumor prototypes: Using the T-UNI models, the author successfully identified two major tumor prototypes:
i. c0: Enriched in RNA splicing and aberrant energy metabolism pathways.
ii. c2: Enriched in TGF-β signaling and associated with epithelial-mesenchymal transition (EMT).
(5) Molecular validation: The work validated the molecular distinctions of these prototypes using spatial transcriptomics data, demonstrating their recurrence across multiple patients and confirming their biological consistency and clinical relevance.

Major comments:
1. The study is limited by its reliance on a small cohort of only 8 HER2-positive breast cancer patients from a single public dataset. Therefore, the findings should be considered preliminary and require validation in larger, independent cohorts before they can be regarded as generalizable conclusions.

Author's response

We thank the reviewer for this insightful comment and fully agree that adding a larger cohort was necessary to validate our study. We have addressed this point by incorporating an additional, independent dataset comprising 92 TNBC patients as well as one sample from high grade ovarian cancer. For a detailed discussion of the validation of our study, we kindly refer the reviewer to our response to Question #2 from Reviewer #3.

2. The study's exclusive focus on HER2-positive breast cancer represents a significant limitation, as it remains unclear whether the identified prototypes and pathways are applicable to other molecular subtypes (e.g., Luminal A/B). This narrow scope limits the generalizability of the findings across the broader breast cancer population.

Authors' response

We thank the reviewer for raising this important point regarding the generalizability of our findings across breast cancer subtypes. We agree that assessing whether the identified tumor archetypes and pathways extend beyond HER2+ breast cancer is essential.

Authors' action

To address this concern, we expanded the study to include an independent and substantially larger spatial transcriptomics dataset comprising 92 TNBC patients, which represents a molecular subtype with biological and morphological characteristics that are markedly distinct from HER2-positive tumors. We revised the manuscript throughout to analyze the HER2 and TNBC datasets in parallel.

Across both subtypes, we demonstrate that our method consistently identifies spatially coherent and biologically meaningful tumor archetypes, supporting the adaptability of the method beyond HER2-positive disease. Notably, we identify a recurrent archetype characterized by dysregulated RNA splicing in both HER2 and TNBC tumors, which is associated with poorer prognosis and aligns with prior reports linking alternative-splicing programs to aggressive breast cancer phenotypes. This convergence across molecularly distinct subtypes supports the biological relevance of the identified pathways.

However, we emphasize that our approach is not designed to train a single universal model transferable across subtypes, but rather to specialize representations to the dataset and tumor context of interest via extended pre-training. As such, the method is transductive in nature and intended to uncover subtype-specific and shared tumor programs, rather than to enforce cross-subtype generalization of learned weights.

While Luminal A/B tumors were not included in the present study due to the lack of comparable spatial transcriptomics datasets with matched histopathology, our results on TNBC provide strong evidence that the proposed framework is applicable beyond HER2-positive breast cancer. We now explicitly discuss this point in the revised manuscript.

3. While the focus on the UNI model is understandable due to computational constraints, the study would be strengthened by a discussion on the potential generalizability of the proposed pre-training strategy. It would be valuable to speculate on whether applying the same approach to models like CTransPath would yield consistent or divergent prototypes.

Author's response

Thank you for this insightful remark. It is well established in the broader machine learning literature that the stronger a pre-trained SSL model is at representing a given domain, the more effective it tends to be when fine-tuned on a related subdomain [7,19]. This understanding motivated our choice of UNI under our computational constraints. However, our sensitivity analysis regarding extended pre-training strategies is particularly relevant to DinoV2-like architectures that rely on teacher–student mechanisms, currently the predominant paradigm for self-supervised learning on images. We therefore believe that our findings are likely to generalize to other histology foundation models (hFMs) built on similar frameworks (e.g., Virchow2, Uni2-h), as well as to future models based on the recent state-of-the-art DinoV3 for natural images. In contrast, purely contrastive models such as CTransPath and Histo-SimCLR use a markedly different set of hyperparameters from those examined in our work and are built on convolutional neural networks rather than vision transformers. As a result, Explora is not directly applicable to these architectures without substantial methodological adaptation.

4. The study's central finding, the identification of two distinct tumor prototypes, warrants further validation in clinical practices. Specifically, the clinical significance of these prototypes remains unclear, as their association with critical outcomes such as patient survival and treatment response has not been investigated.

Authors' response

We thank the reviewer for this valuable suggestion. We fully agree that evaluating the clinical relevance of the identified tumor archetypes is important. To address this, we incorporated an additional dataset of 92 TNBC patients with associated survival data. For a detailed description of the analyses and results, we kindly refer the reviewer to our response to Question #4 from Reviewer #3.

Overall, this work identifies intriguing prototypes but remains at a preliminary stage. The key limitations regarding cohort size and lack of clinical outcome association need to be addressed. Significant strengthening through validation on larger, multi-centric cohorts and demonstration of clinical relevance is necessary to support the conclusions. I regret to conclude that the paper is not yet suitable for publication in its current form.

Authors' response

We thank the reviewer for his valuable review. We hope that the updated significantly revise version is now addressing the concerns.

References

1. Long, Y. *et al.* Spatially informed clustering, integration, and deconvolution of spatial transcriptomics with GraphST. *Nat. Commun.* **14**, 1155 (2023).
2. Hörst, F. *et al.* CellViT: Vision Transformers for precise cell segmentation and classification. *Med. Image Anal.* **94**, 103143 (2024).
3. Kömen, J., Marienwald, H., Dippel, J. & Hense, J. Do Histopathological Foundation Models Eliminate Batch Effects? A Comparative Study. in *Advancements In Medical Foundation Models: Explainability, Robustness, Security, and Beyond* (2024).
4. Macenko, M. *et al.* A method for normalizing histology slides for quantitative analysis. in *2009 IEEE International Symposium on Biomedical Imaging: From Nano to Macro* 1107–1110 (IEEE, 2009).
5. Ciga, O., Xu, T. & Martel, A. L. Self supervised contrastive learning for digital histopathology. *Machine Learning with Applications* **7**, 100198 (2022).
6. Hu, E. J. *et al.* LoRA: Low-Rank Adaptation of Large Language Models. (2021).
7. Oquab, M. *et al.* DINOv2: Learning robust visual features without supervision. *arXiv [cs.CV]* (2023).
8. Chen, R. J. *et al.* Towards a general-purpose foundation model for computational pathology. *Nat. Med.* **30**, 850–862 (2024).
9. Zimmermann, E. *et al.* Virchow2: Scaling self-supervised mixed magnification models in pathology. *arXiv [cs.CV]* (2024).
10. Wang, X. *et al.* Spatial transcriptomics reveals substantial heterogeneity in triple-negative breast cancer with potential clinical implications. *Nat. Commun.* **15**, 10232 (2024).
11. Han, P., Zhu, J., Feng, G., Wang, Z. & Ding, Y. Characterization of alternative splicing events and prognostic signatures in breast cancer. *BMC Cancer* **21**, 587 (2021).
12. Deng, Y. *et al.* Correlations between the characteristics of alternative splicing events, prognosis, and the immune microenvironment in Breast Cancer. *Front. Genet.* **12**, 686298 (2021).
13. Loi, S. *et al.* Tumor infiltrating lymphocytes are prognostic in triple negative breast cancer and predictive for trastuzumab benefit in early breast cancer: results from the FinHER trial. *Ann. Oncol.* **25**, 1544–1550 (2014).
14. Leon-Ferre, R. A. *et al.* Tumor-infiltrating lymphocytes in triple-negative breast cancer. *JAMA* **331**, 1135–1144 (2024).
15. Park, J. H. *et al.* Prognostic value of tumor-infiltrating lymphocytes in patients with early-stage triple-negative breast cancers (TNBC) who did not receive adjuvant chemotherapy. *Ann. Oncol.* **30**, 1941–1949 (2019).
16. Aleixo, E. L., Colonna, J. G., Cristo, M. & Fernandes, E. Catastrophic Forgetting in Deep Learning: A comprehensive taxonomy. *arXiv [cs.LG]* (2023) doi:10.48550/arXiv.2312.10549.
17. Kirkpatrick, J. *et al.* Overcoming catastrophic forgetting in neural networks. *Proc. Natl. Acad. Sci. U. S. A.* **114**, 3521–3526 (2017).
18. Van Assel, H., Vayer, T., Flamary, R. & Courty, N. SNEkhorn: Dimension reduction with symmetric entropic affinities. *arXiv [cs.LG]* 44470–44487 (2023) doi:10.5555/3666122.3668047.
19. He, K. *et al.* Masked Autoencoders Are Scalable Vision Learners. *arXiv [cs.CV]* (2021).

RESPONSE TO REVIEWERS

Reviewer #3

The authors have carefully and comprehensively addressed all of my previous concerns. The revised manuscript is substantially improved in terms of methodological clarity, validation, and biological and clinical relevance. I have some minor comments.

1. Several technical terms are introduced without clear definitions at first mention. For a multidisciplinary readership, brief explanations would be helpful.

Author's response

We appreciate the reviewer's comment and have read carefully the manuscript to ensure readability to the largest audience.

Author's action

In response, we have revised the manuscript to introduce brief definitions of specialized technical terms at their first occurrence, with the goal of improving readability for a multidisciplinary audience. Specifically, we now provide concise explanations of self-supervised learning, vector representations, embeddings and prototypes, extending the list of previously explained terms. In addition, we have added explicit references to the Methods section for more technical concepts such as the Wasserstein distance and Shannon entropy. We believe these revisions improve clarity while preserving the overall flow of the manuscript.

2. The authors have provided a much clearer description of the ExPLoRa extended pre-training strategy, including the role of LoRA adapters, optimization objectives, and hyperparameter choices. Some sensitivity analysis are recommended.

Author's response

As described in the manuscript, a broad set of hyperparameters, including the LoRA rank, regularization coefficients, and optimization parameters, was selected using the default values recommended in the original publications, which have been shown to perform robustly across a wide range of experimental settings. We therefore consider that re-evaluating or re-optimizing these established configurations falls outside the scope of the present work. Instead, our sensitivity analyses focus on aspects most relevant to the study objectives. In particular, analyses of pre-training strategies (full vs ExPLoRa), regularization losses (KDE vs KoLeo), and the number of prototypes are sufficient to critically examine the relevance of key mechanistic behaviors induced by these hyperparameters, such as preserving prior generic knowledge and promoting clustering structure.

Author's action

The "Hyperparameter Selection" paragraph has been updated to clarify that, except for those for which we performed sensitivity analyses, all hyperparameters were adopted as default values already validated in previous studies.

3. The addition of survival analyses, including Cox regression and Kaplan–Meier curves, substantially enhances the translational relevance of the work. The number of event samples in the survival analysis is highly imbalanced, which may lead to statistical bias.

Author's response

We thank the reviewer for this important comment. We acknowledge that the number of events in the survival analysis is relatively limited (16 deaths versus 44 censored observations), which may reduce statistical power and limit generalizability. While the primary emphasis of this manuscript is methodological and technological, the survival-associated patterns observed in this study provide biologically meaningful insights and support the potential translational relevance of the proposed approach. Survival analyses were therefore included to contextualize these findings using publicly available clinical data, without the intention of establishing definitive prognostic conclusions.

To address potential bias from the limited and imbalanced number of events, Cox proportional hazards models were fitted with ridge penalization, following common practice. Penalized Cox regression improves coefficient stability and reduces overfitting in scenarios with few events, thereby offering greater robustness than standard Cox regression. Model performance was evaluated using the concordance index (C-index), which measures the model's ability to correctly rank individuals by relative risk rather than to estimate absolute survival probabilities. Because the C-index relies on pairwise risk ordering and is less sensitive to event imbalance than statistical significance testing or the magnitude of hazard ratios, it remains an appropriate and reliable metric in penalized Cox regression with limited event counts.

We also acknowledge that confidence intervals were initially omitted from the survival analyses. As confidence intervals are essential for interpreting uncertainty and statistical power, they have now been added to the revised Kaplan–Meier figures.

Author's action

We have clarified in the Methods section that Cox regression analyses were performed using ridge penalization which helps mitigate the effects of event imbalance: "we applied ridge penalization with a penalty parameter of 0.1 [...]. *This approach also improves robustness to imbalances in the data, ensuring that the estimated effects are reliable across varying sample distributions.*"

We have also added 95% confidence intervals to the Kaplan-Meier curves (**Fig. 6e**) and adapted the legend accordingly.

The manuscript has been updated to emphasize that further confirmation of clinical relevance will require validation in larger, independent cohorts with the following sentence: "*Although validation in larger, independent cohorts will be required before clinical translation, the present results demonstrate that the identified archetypes achieve high-precision prognostic performance.*"
